# AlloDriver: a method for the identification and analysis of cancer driver targets

**DOI:** 10.1093/nar/gkz350

**Published:** 2019-05-09

**Authors:** Kun Song, Qian Li, Wei Gao, Shaoyong Lu, Qiancheng Shen, Xinyi Liu, Yongyan Wu, Binquan Wang, Houwen Lin, Guoqiang Chen, Jian Zhang

**Affiliations:** 1 Key Laboratory of Cell Differentiation and Apoptosis of Chinese Ministry of Education, Clinical and Fundamental Research Center, Department of Pharmacy, Renji Hospital, Shanghai Jiao-Tong University School of Medicine (SJTU-SM), Shanghai 200127, China; 2 Research Center for Marine Drugs, State Key Laboratory of Oncogenes and Related Genes, Department of Pharmacy, Renji Hospital, Shanghai Jiao-Tong University School of Medicine (SJTU-SM), Shanghai 200127, China; 3 Medicinal Bioinformatics Center, Shanghai Jiao-Tong University School of Medicine (SJTU-SM), Shanghai 200025, China; 4 Shanxi Key Laboratory of Otorhinolaryngology Head and Neck Cancer, Department of Otolaryngology Head & Neck Surgery, the First Hospital, Shanxi Medical University, Taiyuan, Shanxi 030001, China; 5 Department of Pathophysiology, Shanghai Jiao-Tong University School of Medicine (SJTU-SM), Shanghai 200025, China

## Abstract

Identifying the variants that alter protein function is a promising strategy for deciphering the biological consequences of somatic mutations during tumorigenesis, which could provide novel targets for the development of cancer therapies. Here, based on our previously developed method, we present a strategy called AlloDriver that identifies cancer driver genes/proteins as possible targets from mutations. AlloDriver utilizes structural and dynamic features to prioritize potentially functional genes/proteins in individual cancers via mapping mutations generated from clinical cancer samples to allosteric/orthosteric sites derived from three-dimensional protein structures. This strategy exhibits desirable performance in the reemergence of known cancer driver mutations and genes/proteins from clinical samples. Significantly, the practicability of AlloDriver to discover novel cancer driver proteins in head and neck squamous cell carcinoma (HNSC) was tested in a real case of human protein tyrosine phosphatase, receptor type K (PTPRK) through a L1143F driver mutation located at the allosteric site of PTPRK, which was experimentally validated by cell proliferation assay. AlloDriver is expected to help to uncover innovative molecular mechanisms of tumorigenesis by perturbing proteins and to discover novel targets based on cancer driver mutations. The AlloDriver is freely available to all users at http://mdl.shsmu.edu.cn/ALD.

## INTRODUCTION

Cancer is a disease of genetic alterations (1). Advances in DNA sequencing have revealed a broad spectrum of somatic mutations within cancer genomes (2). Somatic mutations include driver and passenger mutations. Compared to passenger mutations, driver mutations confer selective growth advantages towards cancer cells. It is established that cancer driver mutations are involved in 12 major intracellular signaling pathways and regulate three core cellular processes during carcinogenesis, namely, cell survival, cell fate, and genome maintenance (3,4). They are implicated in the acquisition of carcinogenic properties through mediating uncontrolled proliferation, abnormal angiogenesis, metastasis, and drug resistance (1). Considering their central role in tumorigenesis, knowledge of cancer driver mutations cannot only unveil the underlying mechanisms for cancer pathogenesis but can also expand the repertoire of cancer drug targets, which can be further exploited to develop targeted medicine to improve the diagnosis and therapy of cancer.

From a structural standpoint, driver mutations are usually positioned in functional areas, such as allosteric sites (5) and orthosteric sites (6). Allosteric sites are known as regions in a protein that are topologically and spatially distinct from the orthosteric site (7–11). Unlike the well-characterized mutations at orthosteric sites, the landscape of driver mutations at allosteric sites, also referred to as allosteric driver mutations, has been less explored (12,13), because of the complexity of the underlying mechanisms of protein allosteric mutations. It is generally accepted that allosteric driver mutations initiate local conformational disturbances at an allosteric site that propagate to and subsequently alter the conformational state at an orthosteric site (14–18). The resulting effect leads to trapping the protein in either an active or inactive conformation. The abnormal regulation of protein communication caused by allosteric driver mutations leads to tumorigenesis (7,19). Recently, we have developed a statistical approach to identify and prioritize potential allosteric driver mutations in cancer based on systematic analyses of somatic mutations in ∼7000 cancer genomes across 33 cancer types (20). As a result, the identification of cancer-associated allosteric driver mutations and the phenotypes that they alter during tumor initiation and progression could effectively unravel new cancer driver genes/proteins and pathways, decipher their functional consequences and nominate novel druggable targets.

Despite improvements in the understanding of allosteric driver mutations, there is still no efficient and convenient platform for the identification of allosteric driver mutations for cancer therapeutic targets. Based on our previous allosteric data (21) and method (20), here we present an easy-to-use platform called AlloDriver that identifies allosteric driver mutations and assesses their biologically relevant effects on tumor fitness and progression from clinical cancer samples. In addition to allosteric driver mutations, AlloDriver can also recognize orthosteric driver mutations to enable researchers to evaluate cancer-driven targets from mutations as a whole. AlloDriver utilizes both structural and dynamic features to prioritize potentially functional genes/proteins in individual cancers by mapping mutations generated directly from clinical cancer samples to allosteric/orthosteric sites derived from 3D protein structures. Testing on two benchmarking datasets, AlloDriver can reemerge >83% driver mutations at allosteric/orthosteric sites. Furthermore, AlloDriver successfully preferred SHP2 in lung squamous cell carcinomas to be a potential target based on driver mutations from cancer samples. Importantly, we employed AlloDriver to discover an unreported target—human protein tyrosine phosphatase, receptor type K (PTPRK)—in head and neck squamous cell carcinoma (HNSC). It predicted a L1143F driver mutation located at the allosteric site of PTPRK, which was experimentally validated by cell proliferation assay. Collectively, AlloDriver may not only uncover innovative molecular mechanisms of tumorigenesis by the perturbation of protein functions, but may also aid in the identification of novel drug targets based on cancer driver mutations.

## MATERIALS AND METHODS

### Workflow of AlloDriver

AlloDriver is deployed as a computational workflow that identifies therapeutic targets in cancer samples by assessing how mutations at allosteric (and orthosteric) sites perturb protein functions during the proliferation and development of individual cancers, followed by an analysis of the predicted driver mutations among current clinical samples. The web server is free and open to all users with no login requirement.

**Figure 1.**
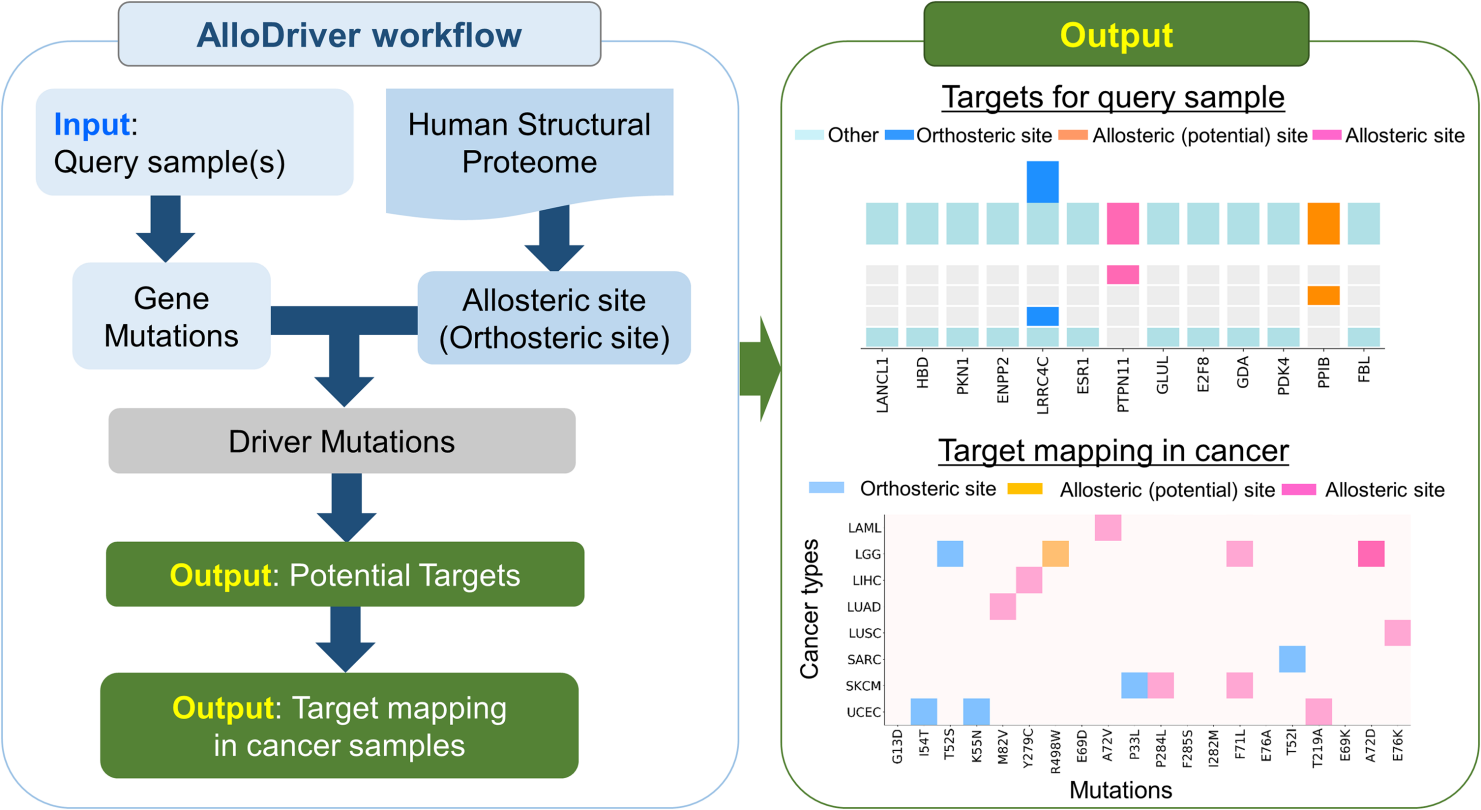
The workflow and output of AlloDriver.

The workflow of AlloDriver is schematically described in Figure 1. First, users can submit cancer samples to AlloDriver, and missense mutations are detected and mapped to 3D structures of 1650 human proteins originating from PDB (https://www.rcsb.org/). Mutations occurring at allosteric or orthosteric sites are further evaluated for driver estimation by the structural and dynamic features. Potential driver proteins in query samples as targets are prioritized on the basis of an evaluation of mutations by the AlloDriver score. In addition, for each query sample, the profiling of predicted driver mutations on the human structural proteome is analyzed. Additionally, for each potential driver protein, clinical mutations in 33 TCGA cancer types at allosteric (orthosteric) sites, their locations of functional domains (22), and known modulators are also provided.

### AlloDriver Input

Clinical sample mutations are required for the identification of potential driver genes/proteins as targets. For users’ convenience, the web server can accept input in three different formats:

1. Upload cancer sample(s) in a Mutation Annotation Format (MAF) file.
2. Upload a tab-delimited output file generated by ANNOVAR software (23). All mutations in the file are considered to be derived from one sample.
3. Specify a valid point mutation list in the text area (e.g. sample_1;BRAF;V600E).

Users can specify the mapping region of interest either as ‘Allosteric site’ or ‘Functional Site’ under ‘Mapping Area’. Currently, AlloDriver provides 2650 allosteric sites (168 experimentally verified allosteric sites and 2482 potential allosteric sites detected by AlloSite (24)) in ‘Allosteric site’, as well as 2650 allosteric sites and 1672 known orthosteric sites in ‘Functional Site’ among 1650 human proteins with 3D structures. Considering the run time, the server does not presently accept samples with >2000 mutations. A ‘Job Name’ is compulsory, which allows users to find their queries in the ‘Job Queue’. Input options in the main page are shown in Supplementary Figure S1 and details of the site collection are provided in the Supplementary Materials and Methods.

### AlloDriver Output

The prediction result is provided to users in the ‘Job Queue’ page when the job is completed. Typically, the web server produces a summary table called the ‘Target Result’, including potential targets ranked by the ‘Score’ of the predicted driver mutations and general information such as ‘Gene/Protein’, ‘UniProt ID’, ‘Driver mutation’, ‘Location’ and ‘Area’ (Supplementary Figure S1). Interactively, clicking the ‘Show’ button in each entry links to details of the driver mutation and its gene/protein to enable users to navigate different analyses for delineating the mutation features for the gene/protein in clinical samples.

Under the summary table, two profiles called ‘Frequency of mutations at different areas’ and ‘Score of potential driver mutations’ are shown for each query sample if available. Users may select one of all samples in the ‘Choose Sample’ menu in the top-left corner of the profiles. In the ‘Frequency of mutations at different areas’ profile, a waterfall subplot shows the distribution of mutations in a query sample mapped at four different areas (allosteric sites, potential allosteric sites, orthosteric sites and other regions) of the human structural proteome and a stacked bar subplot displays the mutation frequency of each mapped protein. Meanwhile, the ‘Score of potential driver mutations’ profile exhibits probability scores for potential driver mutations located at allosteric/orthosteric sites on each predicted human driver protein. Users can download the two profile results via the ‘Download predicted targets’ and ‘Download target analysis’ buttons in this page.

Under the entry for each driver mutation and its protein, a comprehensive analysis and annotation for the mutation and its protein are summarized. In the top, a 3D representation of the predicted driver mutation at the protein is shown in the left panel, together with a table showing the predicted driver mutation's information including ‘Driver Mutation’, ‘Location’, ‘Area’ and ‘Score’ as well as its protein properties such as ‘Gene Symbol’, ‘NCBI Gene ID’, ‘Function’ and ‘PDB ID’ in the right panel. In the middle of the page, a heatmap plot is shown for known mutations of the protein, in which the frequencies of all clinical mutations from TCGA at the allosteric/orthosteric sites of the protein across 33 individual cancer types are illustrated. In addition, there is a lollipop-style diagram that highlights the domain location of potential driver mutations on the protein annotated in PFAM (http://pfam.xfam.org/) in the query samples and the frequencies of the mutations via mapping them into TCGA pan-cancer samples (25). In the bottom, known modulators for the protein are offered with cross-annotated links to two external chemical repositories, DrugBank (https://www.drugbank.ca/) and CHEMBL (https://www.ebi.ac.uk/chembl/) (26,27), and a table summarizing the general information for each modulator, such as the name, molecular weight, 2D structure, original ID in Drugbank or CHEMBL, and its usage for clinical indications. The analysis of a driver mutation and its protein can be downloaded from the server by clicking the ‘Download’ button at the end of the summary table.

It is noted that the runtime of a submitted job can vary from a few minutes to nearly an hour, according to the scale of cohort samples. Conveniently, users can consult the ‘Help’ menu for a step-by-step tutorial.

### Model construction

In the training dataset, the ratio of positive driver mutations to negative passenger mutations is ∼0.12, which could greatly affect the evaluation process and performance of the minority class. To address this problem, the SMOTE (synthetic minority oversampling technology) method was introduced to obtain sufficient data to build a robust model using an imbalanced-learn toolkit (28). The algorithm alters the class distribution by generating new synthetic points from the existing minority samples. Based on our previous study (20), the evaluation model of driver mutations in AlloDriver was employed by combining random forest and multi-layer perceptron methods. All of features are equally weighted in estimator of individual tree and single node of perceptron. Both models were fine-tuned using 10-fold cross-validation to get optimal hyper-parameters. An output score for AlloDriver higher than 0.5 indicates the potential to be a driver mutation. The model construction procedure was executed using the scikit-learn toolkit (29). Random forest is an ensemble method by aggregating decision trees, where each tree is grown using bootstrapped samples by randomly selecting feature subsets and further searching the best split according to the objective function. Here, grid search with default ranges was used to optimize the parameters for random forest as follows: the maximum depth is from 2 to 10, the number of trees is from 10 to 300, and the size of feature subset considered when splitting nodes is from 0.3 to 0.5. After exhaustive searching over the parameter space, these three parameters for the best random forest were determined to be 4, 130 and 0.4. Multi-layer perceptron is a feed-forward artificial neural network, which consists of three layer types: one input layer, one or more hidden layers, and one output layer. The input vector is fed into the neural network architecture, where each layer serves as the input for the next layer by weighted connections. The patterns of input data propagate to the activation function in the output layer to produce a predicted label. The architecture of our multi-layer perceptron consists of the input layer, two hidden layers with 20 and 15 nodes, and the output layer. Similar grid search was used to optimize the parameters and functions for multi-layer perceptron. Finally, the parameters of learning rate and momentum were set to be 0.1 and 0.8, rectified linear unit (ReLU) was used in the hidden layers as activation function, and sigmoid function was applied in the output layer as activation function.

### Benchmarking test dataset

To test the performance of AlloDriver, we built two benchmarking datasets: an allosteric dataset and a functional dataset. The allosteric dataset was setup by collecting driver and passenger mutations at allosteric sites including potential allosteric sites predicted by AlloSite (24), and the functional dataset is composed of driver and passenger mutations at both allosteric and orthosteric sites on human proteins. Driver mutations in the datasets are confirmed from two expert-curated cancer variant knowledge bases: OncoKB (http://oncokb.org/) and CIViC (https://civicdb.org) (30,31). Passenger mutations are aggregated from TCGA cancer samples without known cancer-related functions. To this end, the allosteric dataset is composed of 24 driver mutations and 197 passenger mutations (Supplementary Tables S1 and S2), and the functional dataset contains 73 driver mutations and 582 passenger mutations (Supplementary Tables S3 and S4). The class distributions of the two datasets are shown in Supplementary Figure S2.

## PERFORMANCE OF ALLODRIVER

**Figure 2.**
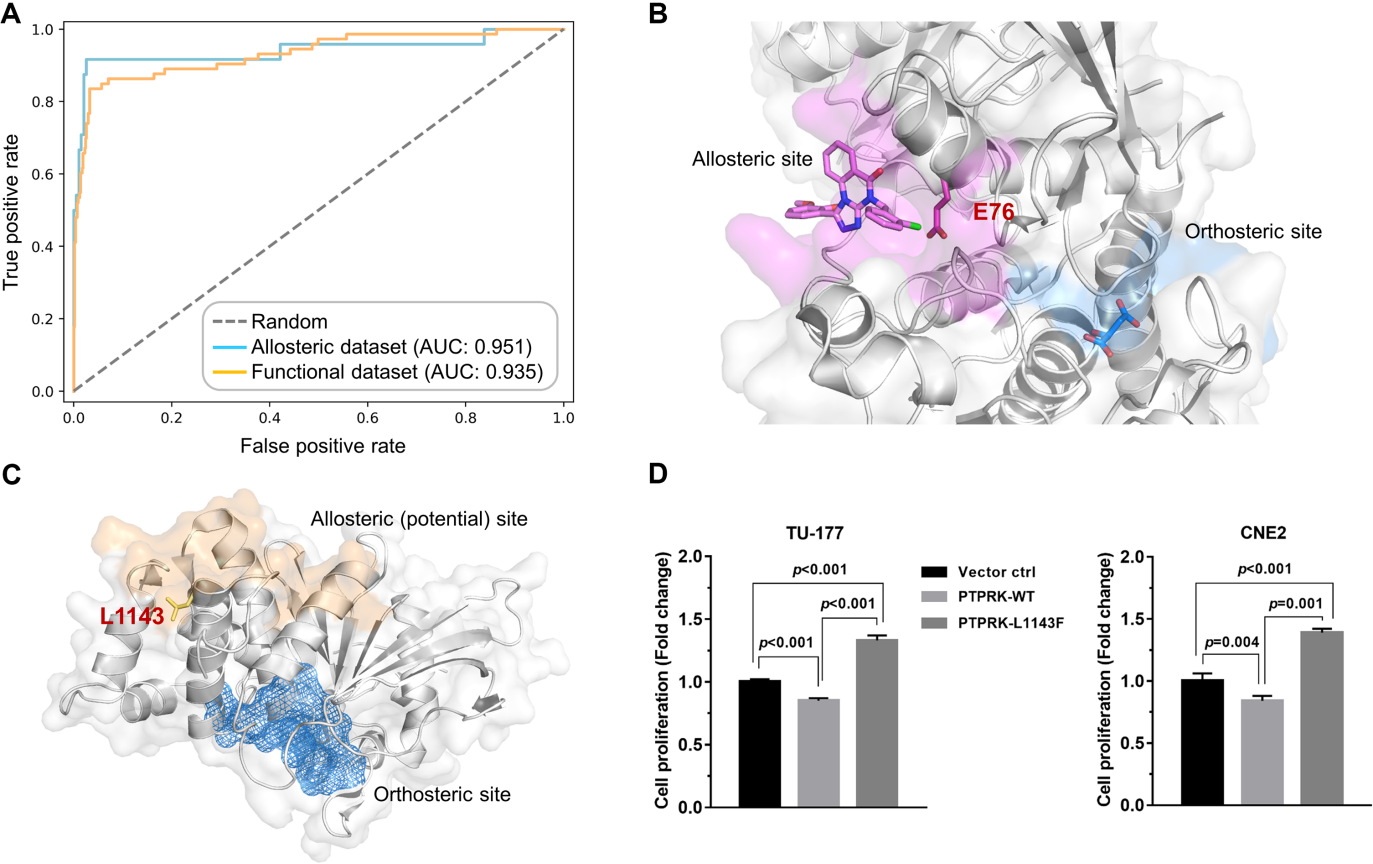
Performance and applications of AlloDriver. (**A**) The Receiver operating characteristic (ROC) curves for the two benchmarking test datasets. (**B**) The driver mutation E76K located at the allosteric site on SHP2 (PTPN11). The protein structure is shown in both cartoon and surface modes. Allosteric and orthosteric sites are colored in magenta and marine, respectively. Residue E76 is highlighted in stick mode. (**C**) The driver mutation L1143F located at the potential allosteric site on PTPRK. The allosteric site is highlighted in wheat on the protein surface, while the orthosteric site is deeply buried and shown as a blue mesh. (**D**) The relative proliferation levels of TU-177 and CNE2 cells overexpressing PTPRK-WT and PTPRK-L1143F protein. Data were normalized to the vector control group. Error bars represent the SD of three to six independent experiments.

To validate the implementation of AlloDriver, we assessed the performance of AlloDriver to identify driver mutations on the two benchmarking datasets: the allosteric dataset and the functional dataset. The result revealed that AlloDriver is capable of detecting 22 out of 24 driver mutations in the allosteric dataset and 61 out of 73 in the functional dataset (Supplementary Table S5), which shows the capability of AlloDriver to distinguish allosteric and orthosteric driver mutations from passenger mutations. Furthermore, we analyzed the receiver operating characteristic (ROC) curves for AlloDriver on the allosteric and functional datasets (Figure 2A). This curve describes the tradeoff between sensitivity and specificity of AlloDriver prediction on the two benchmarking datasets. The further the ROC curve is from the diagonal, the better the AlloDriver prediction is. As another supporting measure, the area under an ROC curve, i.e. the AUC value, was also calculated to reveal the quality of the prediction (32). AlloDriver exhibited excellent performance at any given percentage in the ROC curve and a >0.9 of AUC value for each dataset (allosteric dataset: 0.951 and functional dataset: 0.935), revealing a significantly predictive power for the server based on the well trained model (Supplementary Tables S6–S8).

## EXAMPLES

### Evaluation of driver protein SHP2 in lung squamous cell carcinoma (LUSC)

PTPN11, which encodes the protein tyrosine phosphatase SHP2 with two tandem Src homology 2 (SH2) domains, a PTP domain, and a C-terminal tail, is positively engaged in a variety of intracellular cell signaling cascades and is well-characterized as an oncogene in hematologic malignancies and other solid tumors (33). Using a clinical sample with PTPN11 (SHP2) mutations from LUSC of TGCA and ‘Functional Site’ as inputs, we investigated the potency of AlloDriver to identify the driver protein SHP2. By mapping mutations in the query sample to human structural proteome, three driver proteins were presented according to the potential of predicted driver mutations occurring at allosteric or orthosteric sites. Remarkably, SHP2 was successfully ranked as first of the three driver proteins due to the highest score of 0.647 on E76K as a driver mutation. Figure 2B shows the predicted driver mutation E76K at the allosteric site of SHP2 between the interface of the SH2 domain and the PTP domain, and the result is in consistent with the previous report about the driven effect of SHP2-E76K in tumor progression (34). Furthermore, the pan-cancer analysis of the mutation shows that E76K located at the allosteric site of SHP2 is implicated in not only lung cancer but colon adenocarcinoma, which suggests potentially extensive involvement of SHP2 in tumorigenesis and cancer progression for these individual types. In addition, AlloDriver also provides known inhibitors (e.g. estramustine and estradiol) of SHP2 to promote the rational design of therapeutic agents against these individual cancer types. Detailed information of the example is further provided in the online Tutorial under the Help of the AlloDriver server.

### Discovery of novel driver target PTPRK in HNSC

Head and neck squamous cell carcinoma (HNSC) is the sixth most common cancer worldwide and ∼600 000 new cases are diagnosed each year (35). Despite advances in surgery to treat this disease, the five-year survival rate of HNSC has remained stagnant at ∼50% for the past few decades (36). Thus, it is urgent to identify novel driven targets for HNSC, which will facilitate the development of novel therapeutic approaches for the disease. Using AlloDriver, we found that the protein tyrosine phosphatase, receptor type K (PTPRK) (37) has emerged as a potential driver protein from a patient sample of HNSC. As shown in Figure 2C, PTPRK may play a crucial role in the progression of HNSC through the perturbation of an allosteric driver mutation L1143F as suggested by AlloDriver. To validate the effects of L1143F on PTPRK in HNSC, we transfected wild type PTPRK (PTPRK-wt) or mutant L1143F (PTPRK-L1143F) expression plasmids into two HNSC cell lines, TU-177 and CNE2, respectively (Supplementary Figure S3). In good agreement with the prediction of AlloDriver, both TU-177 and CNE2 cells overexpressing PTPRK-L1143F exhibit a significant increase in proliferation compared with the empty vector control cells or cells expressing PTPRK-wt (Figure 2D). The result indicates that L1143F on PTPRK could potentially be an oncogenic driver mutation in HNSC and then PTPRK could become a target for the treatment of HNSC. Taken together, these data support the feasibility of AlloDriver to discover novel driver proteins as targets via identifying allosteric driver mutations from cancer samples.

## DISCUSSION

Allostery is currently regarded as a unifying mechanism for receptor function and regulation (8), and it is also a novel tactic for target identification in cancer (Supplemental Figures S4 and S5). The AlloDriver platform aims to provide the scientific and industrial community with a free and user-friendly web server to identify allosteric driver mutations for cancer-associated targets. Subsequently, AlloDriver performs clinical profile analyses of predicted allosteric driver mutations. This platform will be continuously updated in the future to make it a useful community resource. One improvement includes future advances in computational methods for the identification of more allosteric sites (38–48). Another improvement stems from the ever-increasing number of protein structures determined by spectroscopy methods (49). These improvements will strengthen the performance of AlloDriver to identify cancer driver mutations (50–52) from clinical cancer samples.

## Supplementary Material

gkz350_Supplemental_Files
