# Supplementary material for: AlloDriver: a method for the identification and analysis of cancer driver targets
Source: Nucleic Acids Res. 2019 May 9;47(W1):W315–21. doi: 10.1093/nar/gkz350 (PMC6602569; doi:10.1093/nar/gkz350)
Supplement: gkz350_Supplemental_Files [file gkz350_supplemental_files.zip › NAR manuscript references with links.docx]

(1-48)

1. Stratton, M.R. (2011) Exploring the genomes of cancer cells: progress and promise. *Science (New York, N.Y.)*, **331**, 1553-1558.

<http://www.ncbi.nlm.nih.gov/pubmed/21436442>

<http://dx.doi.org/10.1126/science.1204040>

2. Bailey, M.H., Tokheim, C., Porta-Pardo, E., Sengupta, S., Bertrand, D., Weerasinghe, A., Colaprico, A., Wendl, M.C., Kim, J., Reardon, B. *et al.* (2018) Comprehensive Characterization of Cancer Driver Genes and Mutations. *Cell*, **173**, 371-385 e318.

<http://www.ncbi.nlm.nih.gov/pubmed/29625053>

<http://dx.doi.org/10.1016/j.cell.2018.02.060>

<http://www.ncbi.nlm.nih.gov/pmc/articles/PMC6029450>

3. Sanchez-Vega, F., Mina, M., Armenia, J., Chatila, W.K., Luna, A., La, K.C., Dimitriadoy, S., Liu, D.L., Kantheti, H.S., Saghafinia, S. *et al.* (2018) Oncogenic Signaling Pathways in The Cancer Genome Atlas. *Cell*, **173**, 321-337 e310.

<http://www.ncbi.nlm.nih.gov/pubmed/29625050>

<http://dx.doi.org/10.1016/j.cell.2018.03.035>

<http://www.ncbi.nlm.nih.gov/pmc/articles/PMC6070353>

4. Nussinov, R. and Tsai, C.J. (2015) 'Latent drivers' expand the cancer mutational landscape. *Current opinion in structural biology*, **32**, 25-32.

<http://www.ncbi.nlm.nih.gov/pubmed/25661093>

<http://dx.doi.org/10.1016/j.sbi.2015.01.004>

5. Kim, P., Zhao, J., Lu, P. and Zhao, Z. (2017) mutLBSgeneDB: mutated ligand binding site gene DataBase. *Nucleic acids research*, **45**, D256-D263.

<http://www.ncbi.nlm.nih.gov/pubmed/27907895>

<http://dx.doi.org/10.1093/nar/gkw905>

<http://www.ncbi.nlm.nih.gov/pmc/articles/PMC5210621>

6. Sedova, M., Iyer, M., Li, Z., Jaroszewski, L., Post, K.W., Hrabe, T., Porta-Pardo, E. and Godzik, A. (2019) Cancer3D 2.0: interactive analysis of 3D patterns of cancer mutations in cancer subsets. *Nucleic acids research*, **47**, D895-D899.

<http://www.ncbi.nlm.nih.gov/pubmed/30407596>

<http://dx.doi.org/10.1093/nar/gky1098>

<http://www.ncbi.nlm.nih.gov/pmc/articles/PMC6324060>

7. Nussinov, R. and Tsai, C.J. (2013) Allostery in disease and in drug discovery. *Cell*, **153**, 293-305.

<http://www.ncbi.nlm.nih.gov/pubmed/23582321>

<http://dx.doi.org/10.1016/j.cell.2013.03.034>

8. Changeux, J.P. and Christopoulos, A. (2016) Allosteric Modulation as a Unifying Mechanism for Receptor Function and Regulation. *Cell*, **166**, 1084-1102.

<http://www.ncbi.nlm.nih.gov/pubmed/27565340>

<http://dx.doi.org/10.1016/j.cell.2016.08.015>

9. van Westen, G.J., Gaulton, A. and Overington, J.P. (2014) Chemical, target, and bioactive properties of allosteric modulation. *PLoS computational biology*, **10**, e1003559.

<http://www.ncbi.nlm.nih.gov/pubmed/24699297>

<http://dx.doi.org/10.1371/journal.pcbi.1003559>

<http://www.ncbi.nlm.nih.gov/pmc/articles/PMC3974644>

10. Huang, Z., Zhao, J., Deng, W., Chen, Y., Shang, J., Song, K., Zhang, L., Wang, C., Lu, S., Yang, X. *et al.* (2018) Identification of a cellularly active SIRT6 allosteric activator. *Nature chemical biology*, **14**, 1118-1126.

<http://www.ncbi.nlm.nih.gov/pubmed/30374165>

<http://dx.doi.org/10.1038/s41589-018-0150-0>

11. Lu, S. and Zhang, J. (2018) Small Molecule Allosteric Modulators of G-Protein-Coupled Receptors: Drug-Target Interactions. *Journal of medicinal chemistry*, aheadofprint.

<http://www.ncbi.nlm.nih.gov/pubmed/29457894>

<http://dx.doi.org/10.1021/acs.jmedchem.7b01844>

12. Tan, Z.W., Tee, W.V., Guarnera, E., Booth, L. and Berezovsky, I.N. (2019) AlloMAPS: allosteric mutation analysis and polymorphism of signaling database. *Nucleic acids research*, **47**, D265-D270.

<http://www.ncbi.nlm.nih.gov/pubmed/30365033>

<http://dx.doi.org/10.1093/nar/gky1028>

<http://www.ncbi.nlm.nih.gov/pmc/articles/PMC6323965>

13. Guarnera, E. and Berezovsky, I.N. (2018) On the perturbation nature of allostery: sites, mutations, and signal modulation. *Current opinion in structural biology*, **56**, 18-27.

<http://www.ncbi.nlm.nih.gov/pubmed/30439587>

<http://dx.doi.org/10.1016/j.sbi.2018.10.008>

14. Guarnera, E. and Berezovsky, I.N. (2016) Structure-Based Statistical Mechanical Model Accounts for the Causality and Energetics of Allosteric Communication. *PLoS computational biology*, **12**, e1004678.

<http://www.ncbi.nlm.nih.gov/pubmed/26939022>

<http://dx.doi.org/10.1371/journal.pcbi.1004678>

<http://www.ncbi.nlm.nih.gov/pmc/articles/PMC4777440>

15. Kurochkin, I.V., Guarnera, E., Wong, J.H., Eisenhaber, F. and Berezovsky, I.N. (2017) Toward Allosterically Increased Catalytic Activity of Insulin-Degrading Enzyme against Amyloid Peptides. *Biochemistry*, **56**, 228-239.

<http://www.ncbi.nlm.nih.gov/pubmed/27982586>

<http://dx.doi.org/10.1021/acs.biochem.6b00783>

16. Nussinov, R., Tsai, C.J. and Liu, J. (2014) Principles of allosteric interactions in cell signaling. *Journal of the American Chemical Society*, **136**, 17692-17701.

<http://www.ncbi.nlm.nih.gov/pubmed/25474128>

<http://dx.doi.org/10.1021/ja510028c>

<http://www.ncbi.nlm.nih.gov/pmc/articles/PMC4291754>

17. Guarnera, E., Tan, Z.W., Zheng, Z. and Berezovsky, I.N. (2017) AlloSigMA: allosteric signaling and mutation analysis server. *Bioinformatics (Oxford, England)*, **33**, 3996-3998.

<http://www.ncbi.nlm.nih.gov/pubmed/29106449>

<http://dx.doi.org/10.1093/bioinformatics/btx430>

18. Pei, J., Yin, N., Ma, X. and Lai, L. (2014) Systems biology brings new dimensions for structure-based drug design. *Journal of the American Chemical Society*, **136**, 11556-11565.

<http://www.ncbi.nlm.nih.gov/pubmed/25061983>

<http://dx.doi.org/10.1021/ja504810z>

19. Lu, S., Jang, H., Muratcioglu, S., Gursoy, A., Keskin, O., Nussinov, R. and Zhang, J. (2016) Ras Conformational Ensembles, Allostery, and Signaling. *Chemical reviews*, **116**, 6607-6665.

<http://www.ncbi.nlm.nih.gov/pubmed/26815308>

<http://dx.doi.org/10.1021/acs.chemrev.5b00542>

20. Shen, Q., Cheng, F., Song, H., Lu, W., Zhao, J., An, X., Liu, M., Chen, G., Zhao, Z. and Zhang, J. (2017) Proteome-Scale Investigation of Protein Allosteric Regulation Perturbed by Somatic Mutations in 7,000 Cancer Genomes. *American journal of human genetics*, **100**, 5-20.

<http://www.ncbi.nlm.nih.gov/pubmed/27939638>

<http://dx.doi.org/10.1016/j.ajhg.2016.09.020>

<http://www.ncbi.nlm.nih.gov/pmc/articles/PMC5223033>

21. Shen, Q., Wang, G., Li, S., Liu, X., Lu, S., Chen, Z., Song, K., Yan, J., Geng, L., Huang, Z. *et al.* (2016) ASD v3.0: unraveling allosteric regulation with structural mechanisms and biological networks. *Nucleic acids research*, **44**, D527-535.

<http://www.ncbi.nlm.nih.gov/pubmed/26365237>

<http://dx.doi.org/10.1093/nar/gkv902>

<http://www.ncbi.nlm.nih.gov/pmc/articles/PMC4702938>

22. Weinstein, J.N., Collisson, E.A., Mills, G.B., Shaw, K.R., Ozenberger, B.A., Ellrott, K., Shmulevich, I., Sander, C. and Stuart, J.M. (2013) The Cancer Genome Atlas Pan-Cancer analysis project. *Nature genetics*, **45**, 1113-1120.

<http://www.ncbi.nlm.nih.gov/pubmed/24071849>

<http://dx.doi.org/10.1038/ng.2764>

<http://www.ncbi.nlm.nih.gov/pmc/articles/PMC3919969>

23. Wang, K., Li, M. and Hakonarson, H. (2010) ANNOVAR: functional annotation of genetic variants from high-throughput sequencing data. *Nucleic acids research*, **38**, e164.

<http://www.ncbi.nlm.nih.gov/pubmed/20601685>

<http://dx.doi.org/10.1093/nar/gkq603>

<http://www.ncbi.nlm.nih.gov/pmc/articles/PMC2938201>

24. Huang, W., Lu, S., Huang, Z., Liu, X., Mou, L., Luo, Y., Zhao, Y., Liu, Y., Chen, Z., Hou, T. *et al.* (2013) Allosite: a method for predicting allosteric sites. *Bioinformatics (Oxford, England)*, **29**, 2357-2359.

<http://www.ncbi.nlm.nih.gov/pubmed/23842804>

<http://dx.doi.org/10.1093/bioinformatics/btt399>

25. Finn, R.D., Coggill, P., Eberhardt, R.Y., Eddy, S.R., Mistry, J., Mitchell, A.L., Potter, S.C., Punta, M., Qureshi, M., Sangrador-Vegas, A. *et al.* (2016) The Pfam protein families database: towards a more sustainable future. *Nucleic acids research*, **44**, D279-285.

<http://www.ncbi.nlm.nih.gov/pubmed/26673716>

<http://dx.doi.org/10.1093/nar/gkv1344>

<http://www.ncbi.nlm.nih.gov/pmc/articles/PMC4702930>

26. Wishart, D.S., Feunang, Y.D., Guo, A.C., Lo, E.J., Marcu, A., Grant, J.R., Sajed, T., Johnson, D., Li, C., Sayeeda, Z. *et al.* (2018) DrugBank 5.0: a major update to the DrugBank database for 2018. *Nucleic acids research*, **46**, D1074-D1082.

<http://www.ncbi.nlm.nih.gov/pubmed/29126136>

<http://dx.doi.org/10.1093/nar/gkx1037>

<http://www.ncbi.nlm.nih.gov/pmc/articles/PMC5753335>

27. Gaulton, A., Hersey, A., Nowotka, M., Bento, A.P., Chambers, J., Mendez, D., Mutowo, P., Atkinson, F., Bellis, L.J., Cibrian-Uhalte, E. *et al.* (2017) The ChEMBL database in 2017. *Nucleic acids research*, **45**, D945-D954.

<http://www.ncbi.nlm.nih.gov/pubmed/27899562>

<http://dx.doi.org/10.1093/nar/gkw1074>

<http://www.ncbi.nlm.nih.gov/pmc/articles/PMC5210557>

28. Chakravarty, D., Gao, J., Phillips, S.M., Kundra, R., Zhang, H., Wang, J., Rudolph, J.E., Yaeger, R., Soumerai, T., Nissan, M.H. *et al.* (2017) OncoKB: A Precision Oncology Knowledge Base. *JCO precision oncology*, **2017**, ppublish.

<http://www.ncbi.nlm.nih.gov/pubmed/28890946>

<http://dx.doi.org/10.1200/po.17.00011>

<http://www.ncbi.nlm.nih.gov/pmc/articles/PMC5586540> and have some equity interest in the company. All other authors have no pertinent conflicts for the purposes of this paper.

29. Griffith, M., Spies, N.C., Krysiak, K., McMichael, J.F., Coffman, A.C., Danos, A.M., Ainscough, B.J., Ramirez, C.A., Rieke, D.T., Kujan, L. *et al.* (2017) CIViC is a community knowledgebase for expert crowdsourcing the clinical interpretation of variants in cancer. *Nature genetics*, **49**, 170-174.

<http://www.ncbi.nlm.nih.gov/pubmed/28138153>

<http://dx.doi.org/10.1038/ng.3774>

<http://www.ncbi.nlm.nih.gov/pmc/articles/PMC5367263>

30. Sonego, P., Kocsor, A. and Pongor, S. (2008) ROC analysis: applications to the classification of biological sequences and 3D structures. *Briefings in bioinformatics*, **9**, 198-209.

<http://www.ncbi.nlm.nih.gov/pubmed/18192302>

<http://dx.doi.org/10.1093/bib/bbm064>

31. Bentires-Alj, M., Paez, J.G., David, F.S., Keilhack, H., Halmos, B., Naoki, K., Maris, J.M., Richardson, A., Bardelli, A., Sugarbaker, D.J. *et al.* (2004) Activating mutations of the noonan syndrome-associated SHP2/PTPN11 gene in human solid tumors and adult acute myelogenous leukemia. *Cancer research*, **64**, 8816-8820.

<http://www.ncbi.nlm.nih.gov/pubmed/15604238>

<http://dx.doi.org/10.1158/0008-5472.can-04-1923>

32. Schneeberger, V.E., Luetteke, N., Ren, Y., Berns, H., Chen, L., Foroutan, P., Martinez, G.V., Haura, E.B., Chen, J., Coppola, D. *et al.* (2014) SHP2E76K mutant promotes lung tumorigenesis in transgenic mice. *Carcinogenesis*, **35**, 1717-1725.

<http://www.ncbi.nlm.nih.gov/pubmed/24480804>

<http://dx.doi.org/10.1093/carcin/bgu025>

<http://www.ncbi.nlm.nih.gov/pmc/articles/PMC4123642>

33. Ferlay, J., Shin, H.R., Bray, F., Forman, D., Mathers, C. and Parkin, D.M. (2010) Estimates of worldwide burden of cancer in 2008: GLOBOCAN 2008. *International journal of cancer*, **127**, 2893-2917.

<http://www.ncbi.nlm.nih.gov/pubmed/21351269>

<http://dx.doi.org/10.1002/ijc.25516>

34. Argiris, A., Karamouzis, M.V., Raben, D. and Ferris, R.L. (2008) Head and neck cancer. *Lancet (London, England)*, **371**, 1695-1709.

<http://www.ncbi.nlm.nih.gov/pubmed/18486742>

<http://dx.doi.org/10.1016/s0140-6736(08)60728-x>

35. Sun, P.H., Ye, L., Mason, M.D. and Jiang, W.G. (2013) Protein tyrosine phosphatase kappa (PTPRK) is a negative regulator of adhesion and invasion of breast cancer cells, and associates with poor prognosis of breast cancer. *Journal of cancer research and clinical oncology*, **139**, 1129-1139.

<http://www.ncbi.nlm.nih.gov/pubmed/23552869>

<http://dx.doi.org/10.1007/s00432-013-1421-5>

36. Lu, S., Huang, W. and Zhang, J. (2014) Recent computational advances in the identification of allosteric sites in proteins. *Drug discovery today*, **19**, 1595-1600.

<http://www.ncbi.nlm.nih.gov/pubmed/25107670>

<http://dx.doi.org/10.1016/j.drudis.2014.07.012>

37. Panjkovich, A. and Daura, X. (2014) PARS: a web server for the prediction of Protein Allosteric and Regulatory Sites. *Bioinformatics (Oxford, England)*, **30**, 1314-1315.

<http://www.ncbi.nlm.nih.gov/pubmed/24413526>

<http://dx.doi.org/10.1093/bioinformatics/btu002>

38. Kaya, C., Armutlulu, A., Ekesan, S. and Haliloglu, T. (2013) MCPath: Monte Carlo path generation approach to predict likely allosteric pathways and functional residues. *Nucleic acids research*, **41**, W249-255.

<http://www.ncbi.nlm.nih.gov/pubmed/23742907>

<http://dx.doi.org/10.1093/nar/gkt284>

<http://www.ncbi.nlm.nih.gov/pmc/articles/PMC3692092>

39. Greener, J.G. and Sternberg, M.J. (2018) Structure-based prediction of protein allostery. *Current opinion in structural biology*, **50**, 1-8.

<http://www.ncbi.nlm.nih.gov/pubmed/29080471>

<http://dx.doi.org/10.1016/j.sbi.2017.10.002>

40. Huang, M., Song, K., Liu, X., Lu, S., Shen, Q., Wang, R., Gao, J., Hong, Y., Li, Q., Ni, D. *et al.* (2018) AlloFinder: a strategy for allosteric modulator discovery and allosterome analyses. *Nucleic acids research*, **46**, W451-W458.

<http://www.ncbi.nlm.nih.gov/pubmed/29757429>

<http://dx.doi.org/10.1093/nar/gky374>

<http://www.ncbi.nlm.nih.gov/pmc/articles/PMC6030990>

41. Tee, W.V., Guarnera, E. and Berezovsky, I.N. (2018) Reversing allosteric communication: From detecting allosteric sites to inducing and tuning targeted allosteric response. *PLoS computational biology*, **14**, e1006228.

<http://www.ncbi.nlm.nih.gov/pubmed/29912863>

<http://dx.doi.org/10.1371/journal.pcbi.1006228>

<http://www.ncbi.nlm.nih.gov/pmc/articles/PMC6023240>

42. Wagner, J.R., Lee, C.T., Durrant, J.D., Malmstrom, R.D., Feher, V.A. and Amaro, R.E. (2016) Emerging Computational Methods for the Rational Discovery of Allosteric Drugs. *Chemical reviews*, **116**, 6370-6390.

<http://www.ncbi.nlm.nih.gov/pubmed/27074285>

<http://dx.doi.org/10.1021/acs.chemrev.5b00631>

<http://www.ncbi.nlm.nih.gov/pmc/articles/PMC4901368>

43. Huang, W., Wang, G., Shen, Q., Liu, X., Lu, S., Geng, L., Huang, Z. and Zhang, J. (2015) ASBench: benchmarking sets for allosteric discovery. *Bioinformatics (Oxford, England)*, **31**, 2598-2600.

<http://www.ncbi.nlm.nih.gov/pubmed/25810427>

<http://dx.doi.org/10.1093/bioinformatics/btv169>

44. Goncearenco, A., Mitternacht, S., Yong, T., Eisenhaber, B., Eisenhaber, F. and Berezovsky, I.N. (2013) SPACER: Server for predicting allosteric communication and effects of regulation. *Nucleic acids research*, **41**, W266-272.

<http://www.ncbi.nlm.nih.gov/pubmed/23737445>

<http://dx.doi.org/10.1093/nar/gkt460>

<http://www.ncbi.nlm.nih.gov/pmc/articles/PMC3692057>

45. Lu, S., He, X., Ni, D. and Zhang, J. (2019) Allosteric Modulator Discovery: From Serendipity to Structure-Based Design. *Journal of medicinal chemistry*, aheadofprint.

<http://www.ncbi.nlm.nih.gov/pubmed/30817889>

<http://dx.doi.org/10.1021/acs.jmedchem.8b01749>

46. Lu, S., Shen, Q. and Zhang, J. (2019) Allosteric Methods and Their Applications: Facilitating the Discovery of Allosteric Drugs and the Investigation of Allosteric Mechanisms. *Accounts of chemical research*, **52**, 492-500.

<http://www.ncbi.nlm.nih.gov/pubmed/30688063>

<http://dx.doi.org/10.1021/acs.accounts.8b00570>

47. Shi, Y. (2014) A glimpse of structural biology through X-ray crystallography. *Cell*, **159**, 995-1014.

<http://www.ncbi.nlm.nih.gov/pubmed/25416941>

<http://dx.doi.org/10.1016/j.cell.2014.10.051>

48. Guo, J., Yu, W., Su, H. and Pang, X. (2017) Genomic landscape of gastric cancer: molecular classification and potential targets. *Science China. Life sciences*, **60**, 126-137.

<http://www.ncbi.nlm.nih.gov/pubmed/27460193>

<http://dx.doi.org/10.1007/s11427-016-0034-1>
