# Supplementary material for: AlloDriver: a method for the identification and analysis of cancer driver targets
Source: Nucleic Acids Res. 2019 May 9;47(W1):W315–21. doi: 10.1093/nar/gkz350 (PMC6602569; doi:10.1093/nar/gkz350)
Supplement: gkz350_Supplemental_Files [file gkz350_supplemental_files.zip › Supplementary file.docx]

***Supplementary Data***

**AlloDriver: a method for the identification and analysis of**

**cancer driver targets**

Kun Song^1,2,||^, Qian Li^1,3,||^, Wei Gao^4,||^, Shaoyong Lu^1,||^, Qiancheng Shen^1,3^, Xinyi Liu^1,2^, Yongyan Wu^4^, Binquan Wang^4^, Houwen Lin^1,2^, Guoqiang Chen^2^, and Jian Zhang^1,2,3,5,*^

^1^ Key Laboratory of Cell Differentiation and Apoptosis of Chinese Ministry of Education, Clinical and Fundamental Research Center, Department of Pharmacy, Renji Hospital, Shanghai Jiao-Tong University School of Medicine (SJTU-SM), Shanghai, 200127, China.

^2^ Research Center for Marine Drugs, State Key Laboratory of Oncogenes and Related Genes, Department of Pharmacy, Renji Hospital, Shanghai Jiao-Tong University School of Medicine (SJTU-SM), Shanghai, 200127, China.

^3^ Medicinal Bioinformatics Center, Shanghai Jiao-Tong University School of Medicine (SJTU-SM), Shanghai, 200025, China.

^4^ Shanxi Key Laboratory of Otorhinolaryngology Head and Neck Cancer, Department of Otolaryngology Head & Neck Surgery, the First Hospital, Shanxi Medical University, Taiyuan, Shanxi, 030001, China.

^5^ Department of Pathophysiology, Shanghai Jiao-Tong University School of Medicine (SJTU-SM), Shanghai, 200025, China.

**Key Words:** AlloDriver, Allostery, Allosteric driver mutation, Cancer target

**^||^**The authors wish it to be known that, in their opinion, the first four authors should be regarded as joint First Authors

***To whom correspondence should be addressed:**

Dr. Jian Zhang, PhD

Tel: +86-21-63846590-776922

Fax: +86-21-64154900

E-mail: jian.zhang@sjtu.edu.cn

**MATERIALS AND METHODS**

**Allosteric and orthosteric sites**

Clinical mutations in query samples can be mapped onto allosteric sites or orthosteric sites on human proteins, both derived from three-dimensional structures. We generated the two benchmark datasets as the following procedures. First, 3683 human proteins with solved structures were retrieved from PDB (<https://www.rcsb.org/>) and UniProt (<https://www.uniprot.org/>). Among them, allosteric sites were recognized from the crystal complex annotation with known allosteric modulators in AlloSteric Database (<http://mdl.shsmu.edu.cn/ASD>). Residues constituting allosteric sites were extracted from the complex structure within 8Å around the allosteric modulator using PyMoL software as described in previous literature (1). In addition to known allosteric sites, AlloSite was employed for detecting potential allosteric sites on the entire human structural proteome (2). Considering the volume for an allosteric site to fit in a “regular” compound, predicted pockets with less than 20 residues were particularly expanded by a distance of 2Å. Then, the allosteric dataset is compiled of a combination of 168 allosteric sites and 2482 potential allosteric sites across 1356 human proteins (Supplementary Figure S4). To construct the functional dataset, the orthosteric sites of the proteins were additionally collected according to the annotation of Pocketome (3), Catalytic Site Atlas (4), BioLiP (5), and literature. All residues constituting the orthosteric site were detected by the fpocket algorithm (6) around the annotated residues and manually checked. As a result, the functional site dataset contains 2650 allosteric sites and 1672 orthosteric sites on 1650 human proteins.

**Training mutation dataset**

Clinical somatic missense mutations at allosteric/orthosteric sites on the human proteins in training dataset were derived from two cancer mutation resources: The Cancer Genome Atlas (TCGA) and the Catalog of Somatic Mutations in Cancer (COSMIC) (7, 8). Allosteric and orthosteric mutations were divided into driver and passenger mutations in terms of cancer-associated functional consequences annotated in six databases: OncoKB (http://oncokb.org/), CIViC (https://civicdb.org/), DoCM (<http://docm.info>), CGI (<https://www.cancergenomeinterpreter.org/>), IntoGen (https://www.intogen.org/) and PMKB (<https://pmkb.weill.cornell.edu/>) (9-14). Finally, the dataset comprises 357 driver mutations and 2925 passenger mutations (Supplementary Table S6, S7). Supplementary Figure S5 illustrates the distribution of driver and passenger mutations across the human proteins in the training dataset.

**Feature representation**

According to our previous method (1), AlloDriver combines conventional sequence features with structural and dynamic features to account for the complexities of perturbation elicited by driver mutations at both atomic and molecular levels. Sequence features include conservation scores of the mutated position, scoring matrices for evolutionary distance, and change in physicochemical properties, computed by the KAlign software (15), the ProDy (16) and BioPython toolkit (17). Basic structural features are mainly regarded as secondary structure assignments, residue accessibility, and geometry characterization, which are all implemented by the BioPython (17) toolkit and DSSP software (18). Moreover, AlloDriver incorporates interaction features, generated from the RING software, by elucidating chemical contacts the mutated residue forms in the residue network (19). Importantly, dynamic features are further considered in AlloDriver to measure the residue perturbation in the context of local allosteric/orthosteric pockets as well as the global structure from normal mode analysis performed with the ProDy package (16). In addition, one energy term is evaluated for protein stability changes upon point mutations using the I-mutant software (20). A total of 42 features are summarized in Supplementary Table S8.

**Input MAF file**

A Mutation Annotation Format (MAF) file (.maf) is a human-readable and tab-delimited text file that lists mutations derived from sequencing cancer cohort samples. The format originates from The Cancer Genome Atlas (TCGA) project and has been widely accepted by researchers since the detected mutations in a MAF file can be easily viewed, edited or processed for custom mining. Each line in a MAF file represents a cancer mutation, which is annotated by many fields ranging from chromosome names to protein information. Column headers and ordering may sometimes vary between files of different sources. In AlloDriver, the MAF file is composed of four headers as follows: (1) "Hugo_Symbol" for gene symbol, e.g. BRAF; (2) "HGVSp_Short" for detected mutation, e.g. p.V600E; (3) "Variant_Classification" for translational effect of variant allele, e.g. Missense_Mutation; (4) "Tumor_Sample_Barcode" for cancer sample ID. More headers in MAF file are described in the GDC documentation of NIH (https://docs.gdc.cancer.gov/Data/File_Formats/MAF_Format/).

**Mutational landscape for the driver target**

For a driver target predicted by AlloDriver, MAF files of somatic mutation data across 33 cancer types using MuTect pipeline were obtained from the TCGA data portal. Then, missense mutations in pan-cancer samples were mapped onto allosteric/orthosteric sites on the driver target and frequencies were computed for individual cancer type. In addition, domain annotations were retrieved from the PFAM database for 1650 human proteins (21). Functional mutations in query samples with their frequencies in pan-cancer samples were mapped onto annotated domains for the driver target. The analyses are represented by a heatmap and a lollipop-style diagram implemented using the matplotlib toolkit (22) and trackviewer R package (23).

**Cell Culture and transfection**

The human laryngeal squamous cell carcinoma cell line TU-177 obtained from Bioleaf Biotech was cultured in MEM supplemented with a 10% fetal bovine serum (Biological Industries). The human nasopharyngeal carcinoma cell line CNE2 obtained from Bena Culture Collection was cultured in RPMI1640 supplemented with a 10% fetal bovine serum. Cells were maintained in a complete medium containing 100 U/mL penicillin and 100 μg/mL streptomycin in a humidified chamber at 37 °C supplied with 5% CO2. Transfection was conducted using the Lipofectamine 3000 reagent (Thermo Fisher Scientific) following the manufacturer’s instructions.

**Plasmid Construction**

The full-length coding sequence of the human protein tyrosine phosphatase, receptor type K (PTPRK, GenBank: NM_001291984.1) was generated by PCR using human cDNA as a template. Next, the full-length wild type (PTPRK-WT) and mutant PTPRK-L1143F (generated by overlapping extension PCR) were sub-cloned into the NotI and KpnI sites of the p3×Flag-CMV-10 vector (Sigma-Aldrich) respectively. The resulting plasmids p3×Flag-CMV-PTPRK-WT and p3×Flag-CMV-PTPRK-L1143F were verified by sequencing. The primer sequences used for the construction of plasmids are as follows: PTPRK-F: GCTTGCGGCCGCGATGGATACGACTGCGGCGGCGGCG, PTPRK-R: ATCGGTACCCTAAGATGATTCCAGGTACTCCAA. For generation of the L1143F sequence, the below primers containing mutations were used: L1143F-F: CCATTTTAGAAGCCTGCTTCTGTGGAGAAACTGCCATAC, L1143F-R: GTATGGCAGTTTCTCCACAGAAGCAGGCTTCTAAAATGG.

**Western Blotting**

Cells were lysed in an RIPA buffer (Thermo Fisher Scientific), and then boiled at 98 ℃ for 5 min with a 1×Protein Loading Buffer (Shanghai Yeasen Biotechnology Co., Ltd). Protein samples were separated by SDS-PAGE and then transferred onto PVDF membranes (Millipore). The membranes were blocked with 5% non-fat milk at room temperature for 2 hours and then incubated with an antibody against Flag (Sigma-Aldrich) or GAPDH (TransGen Biotech) at 4 ℃ overnight. The membranes were washed by TBST 3 times, followed by incubation with the corresponding HRP-conjugated secondary antibodies for 1 hour at room temperature. After 3 TBST washes, the membranes were detected using the WesternBright ECL HRP substrate (Advansta Inc.).

**Cell proliferation assay**

Cell proliferation was determined using the Cell Titer-Glo Luminescent Cell Viability Assay kit (Promega) following the manufacturer’s instructions. Briefly, 5000 cells were seeded on opaque-walled 96-well plates, and then transfected with the p3×Flag-CMV-10 empty vector, p3×Flag-CMV-PTPRK-WT or p3×Flag-CMV-PTPRK-L1143F plasmids. 48 hours after transfection, the plate was equilibrated at room temperature for 30 minutes. 100µl Cell Titer-Glo Reagent was added to the medium containing the cells, and the contents were mixed for two minutes on an orbital shaker to induce cell lysis. The plate was incubated at room temperature for 10 minutes, and then the luminescence was detected on a SpectraMax i3x Multi-Mode Microplate Reader (Molecular Devices).

**Statistical Analysis**

All experiments were repeated a minimum of three times. Statistical analysis was performed using the Student’s t test. A p value < 0.05 was considered to be statistically significant.

**REFERENCES**

1. Shen,Q., Cheng,F., Song,H., Lu,W., Zhao,J., An,X., Liu,M., Chen,G., Zhao,Z. and Zhang,J. (2017) Proteome-Scale Investigation of Protein Allosteric Regulation Perturbed by Somatic Mutations in 7,000 Cancer Genomes. *Am. J. Hum. Genet.*, **100**, 5–20. DOI:10.1016/j.ajhg.2016.09.020
2. Huang,W., Lu,S., Huang,Z., Liu,X., Mou,L., Luo,Y., Zhao,Y., Liu,Y., Chen,Z., Hou,T., *et al.* (2013) Allosite: A method for predicting allosteric sites. *Bioinformatics*, **29**, 2357–2359. DOI:10.1093/bioinformatics/btt399
3. Kufareva,I., Ilatovskiy,A.V. and Abagyan,R. (2012) Pocketome: an encyclopedia of small-molecule binding sites in 4D, *Nucleic Acids Res*., **40**, D535-D540. DOI:10.1093/nar/gkr825
4. Furnham,N., Holliday,G.L., DeBeer,T.A.P., Jacobsen,J.O.B., Pearson,W.R. and Thornton,J.M. (2014) The Catalytic Site Atlas 2.0: cataloging catalytic sites and residues identified in enzymes. *Nucleic Acids Res.*, **42**, D485–D489. DOI:10.1093/nar/gkt1243
5. Yang,J., Roy,A. and Zhang,Y. (2013) BioLiP: a semi-manually curated database for biologically relevant ligand-protein interactions. *Nucleic Acids Res.*, **41**, D1096–D1103. DOI:10.1093/nar/gks966
6. Le Guilloux,V., Schmidtke,P. and Tuffery,P. (2009) Fpocket: an open source platform for ligand pocket detection. *BMC Bioinformatics*, **10**, 168. DOI:10.1186/1471-2105-10-168
7. Weinstein,J.N., Collisson,E.A., Mills,G.B., Shaw,K.R., Ozenberger,B.A., Ellrott,K., Shmulevich,I., Sander,C. and Stuart,J.M. (2013) The Cancer Genome Atlas Pan-Cancer analysis project. *Nat. Genet.*, **45**, 1113–1120. DOI:10.1038/ng.2764
8. Tate,J.G., Bamford,S., Jubb,H.C., Sondka,Z., Beare,D.M., Bindal,N., Boutselakis,H., Cole,C.G., Creatore,C., Dawson,E., *et al.* (2019) COSMIC: the Catalogue Of Somatic Mutations In Cancer. *Nucleic Acids Res.*, **47**, D941–D947. DOI:10.1093/nar/gky1015
9. Chakravarty,D., Gao,J., Phillips,S.M., Kundra,R., Zhang,H., Wang,J., Rudolph,J.E., Yaeger,R., Soumerai,T., Nissan,M.H., *et al.* (2017) OncoKB: a precision oncology knowledge base. *JCO Precis. Oncol.*, doi:10.1200/PO.17.00011. DOI:10.1200/PO.17.00011
10. Griffith,M., Spies,N.C., Krysiak,K., McMichael,J.F., Coffman,A.C., Danos,A.M., Ainscough,B.J., Ramirez,C.A., Rieke,D.T., Kujan,L., *et al.* (2017) CIViC is a community knowledgebase for expert crowdsourcing the clinical interpretation of variants in cancer. *Nat. Genet.*, **49**, 170–174. DOI:10.1038/ng.3774
11. Ainscough,B.J., Griffith,M., Coffman,A.C., Wagner,A.H., Kunisaki,J., Choudhary,M.N., McMichael,J.F., Fulton,R.S., Wilson,R.K., Griffith,O.L., *et al.* (2016) DoCM: a Database of curated mutations in cancer. *Nat. Methods*, **13**, 806–807. DOI:10.1038/nmeth.4000
12. Tamborero,D., Rubio-Perez,C., Deu-Pons,J., Schroeder,M.P., Vivancos,A., Rovira,A., Tusquets,I., Albanell,J., Rodon,J., Tabernero,J., *et al.* (2018) Cancer Genome Interpreter annotates the biological and clinical relevance of tumor alterations. *Genome Med.*, **10**, 25–32. DOI:10.1186/s13073-018-0531-8
13. Gonzalez-Perez,A., Perez-Llamas,C., Deu-Pons,J., Tamborero,D., Schroeder,M.P., Jene-Sanz,A., Santos,A. and Lopez-Bigas,N. (2013) IntOGen-mutations identifies cancer drivers across tumor types. *Nat. Methods*, **10**, 1081–1082. DOI:10.1038/nmeth.2642
14. Huang,L., Fernandes,H., Zia,H., Tavassoli,P,, Rennert,H., Pisapia,D., Imielinski,M., Sboner,A., Rubin,M.A., *et al.* (2017) The cancer precision medicine knowledge base for structured clinical-grade mutations and interpretations. *J. Am. Med. Inform. Assoc.*, **24**, 513–519. DOI:10.1093/jamia/ocw148
15. Lassmann,T. and Sonnhammer,E.L. (2005) Kalign–an accurate and fast multiple sequence alignment algorithm. *BMC Bioinformatics*, **6**, 298. DOI:10.1186/1471-2105-6-298
16. Bakan,A., Meireles,L.M and Bahar,I. (2011) ProDy: protein dynamics inferred from theory and experiments. *Bioinformatics*, **27**, 1575-1577. DOI:10.1093/bioinformatics/btr168
17. Cock,P.J., Antao,T., Chang,J.T., Chapman,B.A., Cox,C.J., Dalke,A., Friedberg,I., Hamelryck,T., Kauff,F., Wilczynski,B., *et al.* (2009) Biopython: freely available Python tools for computational molecular biology and bioinformatics. *Bioinformatics*, **25**, 1422–1423. DOI:10.1093/bioinformatics/btp163
18. Kabsch,W. and Sander,C. (1983) Dictionary of protein secondary structure: pattern recognition of hydrogen-bonded and geometrical features. *Biopolymers*, **22**, 2577–2637. DOI:10.1002/bip.360221211
19. Piovesan,D., Minervini,G. and Tosatto,S.C.E. (2016) The RING 2.0 web server for high quality residue interaction networks. *Nucleic Acids Res.*, **44**, W367–W374. DOI:10.1093/nar/gkw315
20. Capriotti,E, Fariselli,P and Casadio,R. (2005) I-Mutant2.0: predicting stability changes upon mutation from the protein sequence or structure. *Nucleic Acids Res.*, **33**, W306-W310. DOI:10.1093/nar/gki375
21. Finn,R.D., Coggill,P., Eberhardt,R.Y., Eddy,S.R., Mistry,J., Mitchell,A.L., Potter,S.C., Punta,M., Qureshi,M., Sangrador-Vegas,A., *et al.* (2016) The Pfam protein families database: towards a more sustainable future. *Nucleic Acids Res.*, **44**, D279–D285. DOI:10.1093/nar/gkv1344
22. Hunter,J.D. (2007) Matplotlib: a 2D graphics environment. *Comput. Sci. Eng.*, **9**, 90–95. DOI: [10.1109/MCSE.2007.55](https://doi.org/10.1109/MCSE.2007.55)
23. Ou,J., Wang Y. and Zhu,L. (2018). trackViewer: A R/Bioconductor package for drawing elegant interactive tracks or lollipop plot to facilitate integrated analysis of multi-omics data. R package version 1.18.0.

**FIGURES**

**
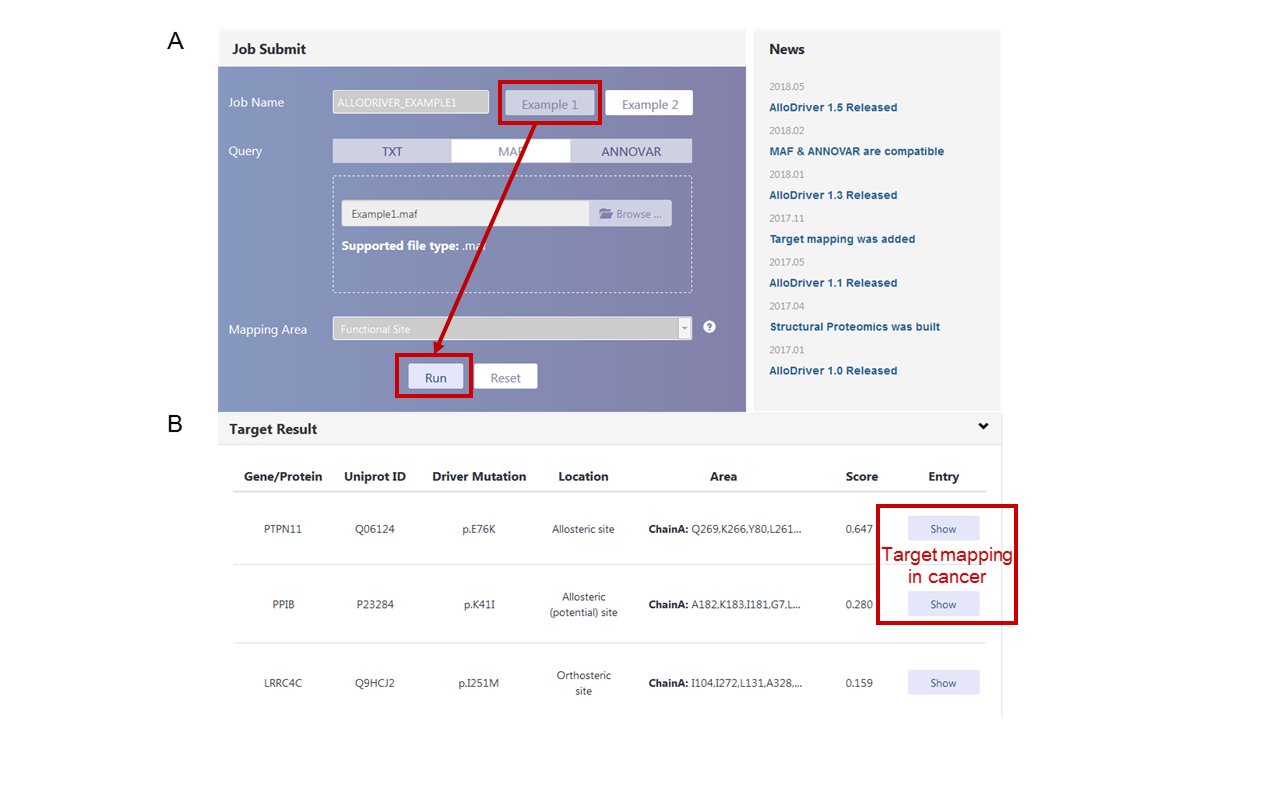
**

**Figure S1.** Screenshots of the procedure from Example 1 show an application of the AlloDriver webserver to discover a cancer-driven target in LUSC. (A) Input parameters for AlloDriver (B) Output table for the job.


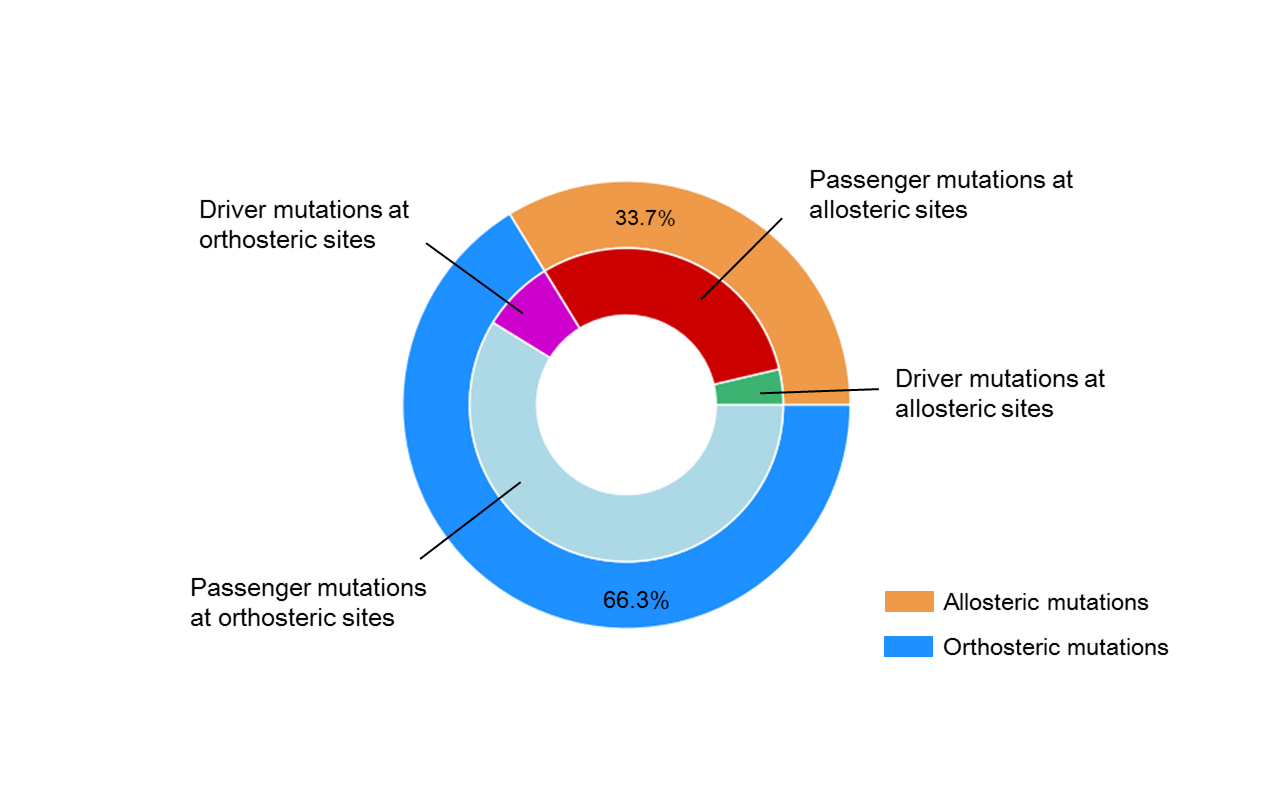


**Figure S2.** The class distribution in two benchmarking dataset. The allosteric dataset is shown in orange in the outer circle, while the functional dataset is the combination of allosteric and orthosteric mutations (blue). The inner circle exhibits the driver and passenger mutation distribution. Ratios of passenger mutations to driver mutations in the two datasets are similar to that in the training dataset.


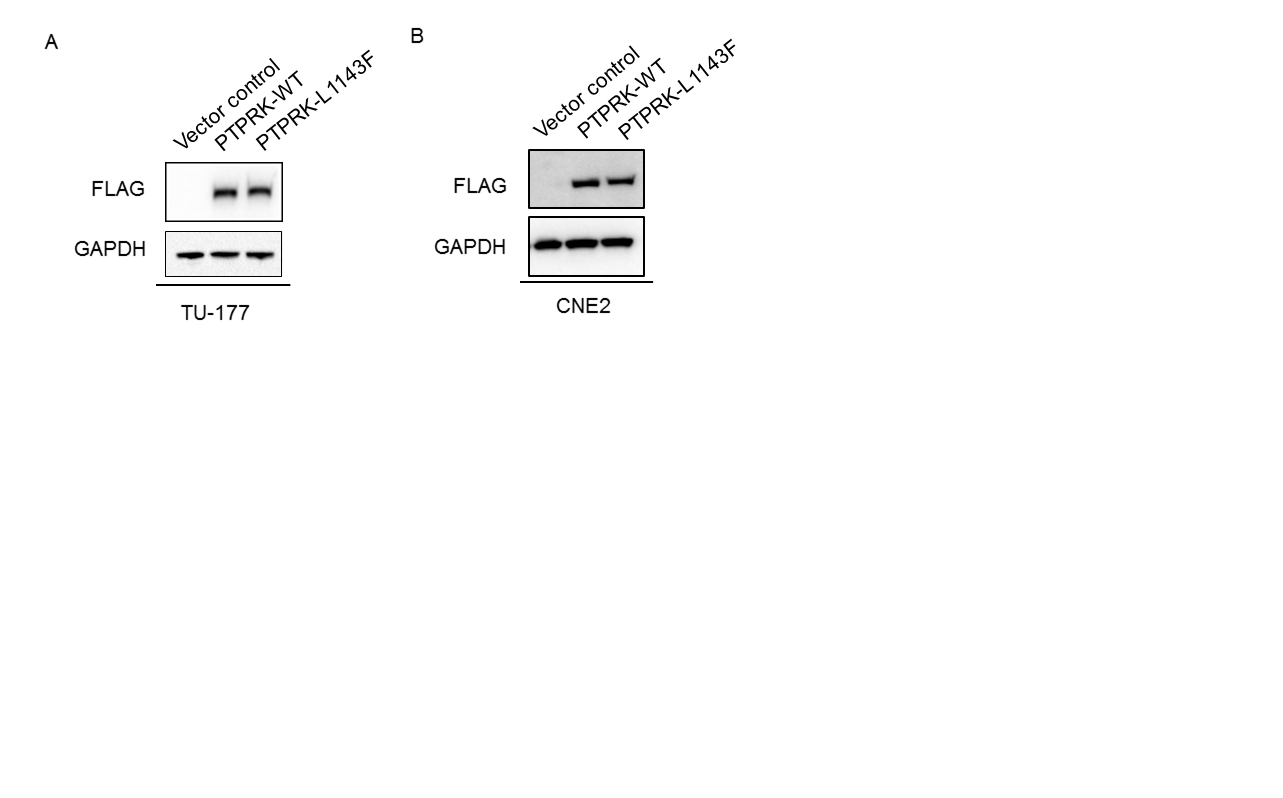


**Figure S3.** Expression of PTPRK-WT and PTPRK-L1143F in (A) TU-177 and (B) CNE2 cell lines was detected by western blotting. The TU-177 and CNE2 cells were transfected with an empty vector p3×FLAG-CMV-10 (Vector ctrl), p3×FLAG-CMV-PTPRK-WT (PTPRK-WT) or p3×FLAG-CMV-PTPRK-L1143F (PTPRK-L1143F) for 48 hours.


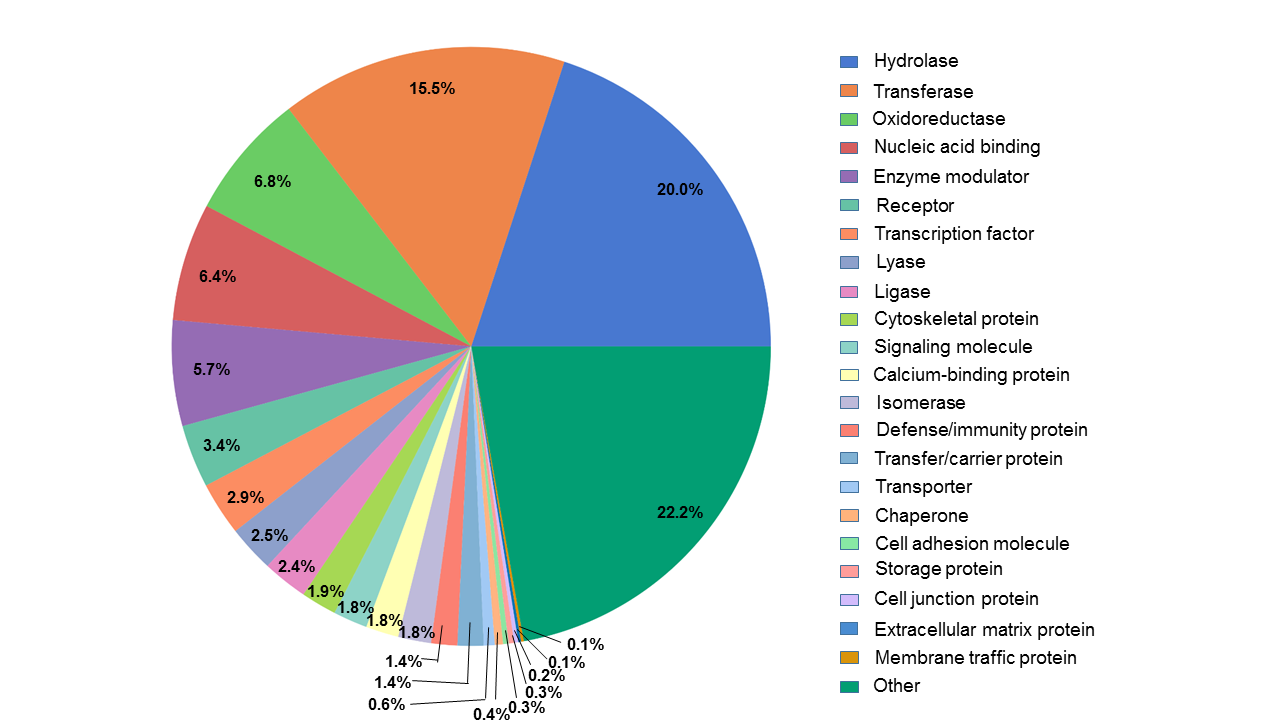


**Figure S4.** The class coverage of 1356 human proteins with allosteric sites.


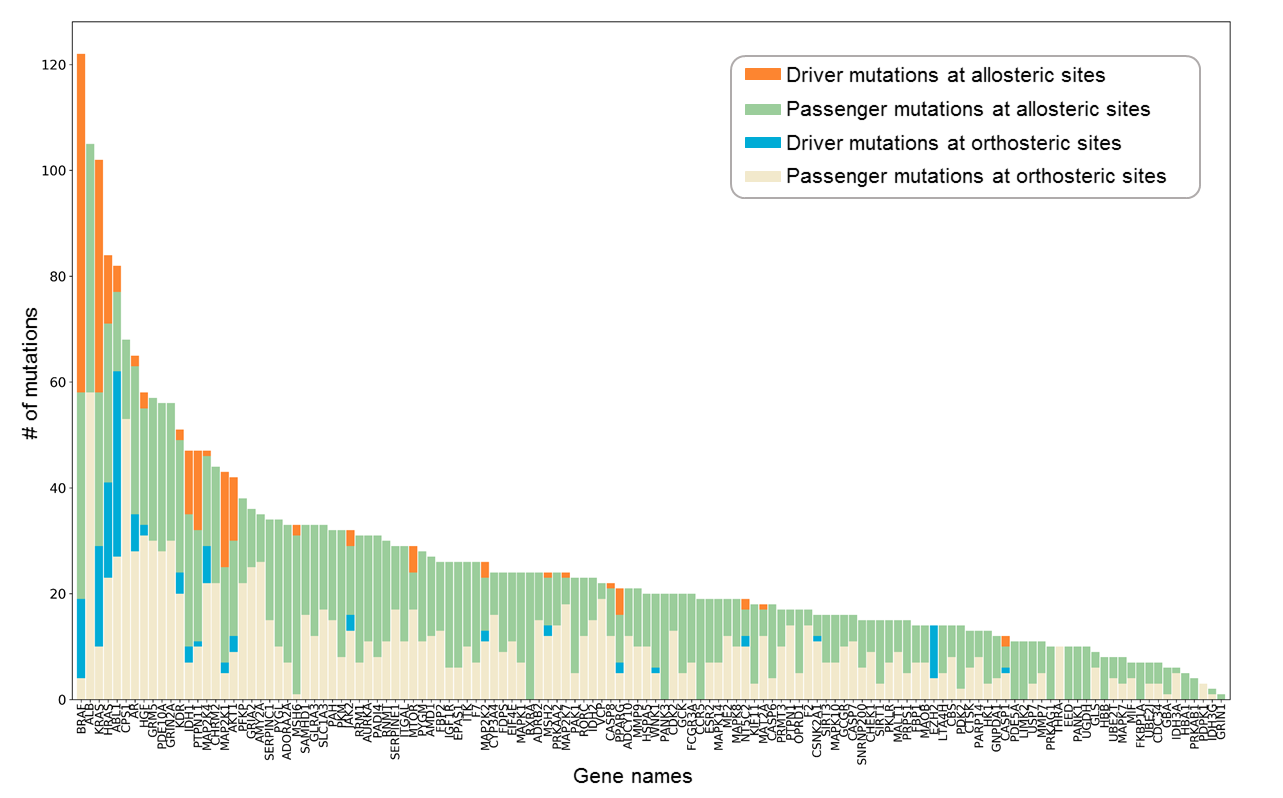


**Figure S5.** A stacked bar chart for allosteric and orthosteric mutations across proteins in the training dataset. Driver mutations are colored in orange and deep-sky blue, while passenger mutations are colored in pale green and corn silk.

**TABLES**

**Table S1.** Allosteric driver mutations in the allosteric dataset.

| Index | Gene name | Uniprot ID | Mutation | Index | Gene name | Uniprot ID | Mutation |
| --- | --- | --- | --- | --- | --- | --- | --- |
| 1 | ALK | Q9UM73 | M1328I | 13 | PTEN | P60484 | N276S |
| 2 | EGFR | P00533 | V769M | 14 | PTEN | P60484 | G251C |
| 3 | EP300 | Q09472 | P1502L | 15 | PTEN | P60484 | Y174N |
| 4 | FGFR2 | P21802 | R678G | 16 | ROS1 | P08922 | C2060G |
| 5 | FGFR2 | P21802 | K526E | 17 | SMAD3 | P84022 | R373Q |
| 6 | FGFR2 | P21802 | K659E | 18 | TP53 | P04637 | L145R |
| 7 | MAP2K1 | Q02750 | P306H | 19 | TP53 | P04637 | Y220C |
| 8 | MTOR | P42345 | L2230V | 20 | TP53 | P04637 | D186A |
| 9 | PTEN | P60484 | R173H | 21 | TP53 | P04637 | P153H |
| 10 | PTEN | P60484 | F347L | 22 | TP53 | P04637 | R110P |
| 11 | PTEN | P60484 | P96Q | 23 | TP53 | P04637 | T150I |
| 12 | PTEN | P60484 | L345Q | 24 | TP53 | P04637 | C229R |

**Table S2.** Allosteric passenger mutations in the allosteric dataset.

| Index | Gene name | Uniprot ID | Mutation | Index | Gene name | Uniprot ID | Mutation |
| --- | --- | --- | --- | --- | --- | --- | --- |
| 1 | A2M | P01023 | R117W | 100 | IL1RL1 | Q01638 | V316I |
| 2 | A2M | P01023 | P631T | 101 | IL22RA2 | Q969J5 | K189N |
| 3 | ACAA1 | P09110 | R249H | 102 | ILK | Q13418 | K448N |
| 4 | ACADS | P16219 | R330H | 103 | IMPA2 | O14732 | D255N |
| 5 | ACAT1 | P24752 | Q211R | 104 | KCNK1 | O00180 | L146V |
| 6 | ACHE | P22303 | T467M | 105 | KIF2C | Q99661 | I259T |
| 7 | ACHE | P22303 | P477S | 106 | KIF3B | O15066 | R336H |
| 8 | ACMSD | Q8TDX5 | D270E | 107 | LCK | P06239 | H208N |
| 9 | ACVRL1 | P37023 | V380G | 108 | LCK | P06239 | C217Y |
| 10 | ACYP1 | P07311 | S93L | 109 | LDHB | P07195 | S168Y |
| 11 | ADAM8 | P78325 | D263N | 110 | LILRB1 | Q8NHL6 | N203K |
| 12 | ADAT2 | Q7Z6V5 | E38K | 111 | LIPF | P07098 | S300Y |
| 13 | ADH1B | P00325 | K19N | 112 | LYPLA1 | O75608 | S7L |
| 14 | AGXT | P21549 | W332C | 113 | MAPK10 | P53779 | L393R |
| 15 | AHCY | P23526 | C421Y | 114 | MARK3 | P27448 | S215C |
| 16 | AHCY | P23526 | P427L | 115 | MASP2 | O00187 | Y410N |
| 17 | AHCYL2 | Q96HN2 | D582N | 116 | MCPH1 | Q8NEM0 | E681K |
| 18 | AK4 | P27144 | S187N | 117 | MCPH1 | Q8NEM0 | R791H |
| 19 | ALDH1A1 | P00352 | F425L | 118 | MDH2 | P40926 | P87S |
| 20 | ALDOB | P05062 | Y204H | 119 | MECR | Q9BV79 | I301M |
| 21 | ALDOB | P05062 | T260N | 120 | MEPCE | Q7L2J0 | R443W |
| 22 | ALOX12 | P18054 | G454E | 121 | MMAA | Q8IVH4 | V220L |
| 23 | ANPEP | P15144 | E636K | 122 | MMAA | Q8IVH4 | R316C |
| 24 | ANXA5 | P08758 | D20N | 123 | MME | P08473 | R748W |
| 25 | AOC1 | P19801 | N110K | 124 | MPG | P29372 | G189D |
| 26 | AOC1 | P19801 | Q353E | 125 | MPO | P05164 | L333F |
| 27 | AOC3 | Q16853 | A456V | 126 | MPO | P05164 | Q696H |
| 28 | AOC3 | Q16853 | E556Q | 127 | MPO | P05164 | G451W |
| 29 | AOX1 | Q06278 | R833C | 128 | MYOC | Q99972 | Y437C |
| 30 | ARHGDIB | P52566 | E190Q | 129 | NAMPT | P43490 | K229T |
| 31 | ASPH | Q12797 | K336T | 130 | NFYC | Q13952 | D116N |
| 32 | ASRGL1 | Q7L266 | G214E | 131 | NOTUM | Q6P988 | G230R |
| 33 | ASRGL1 | Q7L266 | A94T | 132 | OCRL | Q01968 | R552Q |
| 34 | ATXN3 | P54252 | E43Q | 133 | ODC1 | P11926 | D364N |
| 35 | BCHE | P06276 | L301I | 134 | OTUB2 | Q96DC9 | K73T |
| 36 | BHMT | Q93088 | D263N | 135 | P4HB | P07237 | A118G |
| 37 | CA12 | O43570 | P73H | 136 | PAICS | P22234 | K283T |
| 38 | CA13 | Q8N1Q1 | R81H | 137 | PASK | Q96RG2 | F1076L |
| 39 | CA4 | P22748 | P39A | 138 | PC | P11498 | P524H |
| 40 | CAMK2D | Q13557 | C127F | 139 | PDE3B | Q13370 | R694T |
| 41 | CAMKK2 | Q96RR4 | P431S | 140 | PDE3B | Q13370 | D846Y |
| 42 | CBS | P35520 | D234N | 141 | PDE3B | Q13370 | R881H |
| 43 | CD38 | P28907 | R66Q | 142 | PDHA1 | P08559 | Q84H |
| 44 | CD38 | P28907 | R66P | 143 | PDK4 | Q16654 | R161Q |
| 45 | CDC42 | P60953 | D57G | 144 | PGD | P52209 | E436Q |
| 46 | CISD3 | P0C7P0 | K48T | 145 | PGK1 | P00558 | N195S |
| 47 | CP | P00450 | A530S | 146 | PHGDH | O43175 | T125M |
| 48 | CP | P00450 | L666M | 147 | PHYH | O14832 | K59N |
| 49 | CPA2 | P48052 | R386H | 148 | PIK3CG | P48736 | V162I |
| 50 | CPS1 | P31327 | D1277Y | 149 | PIK3CG | P48736 | R690I |
| 51 | CSAD | Q9Y600 | E45Q | 150 | PIP4K2A | P48426 | S48W |
| 52 | CSAD | Q9Y600 | R380H | 151 | PIP4K2B | P78356 | M65V |
| 53 | CTH | P32929 | E345K | 152 | PIP4K2C | Q8TBX8 | P131L |
| 54 | CTPS1 | P17812 | S156G | 153 | PNLIPRP2 | P54317 | F149L |
| 55 | CTSL | P07711 | G252V | 154 | POFUT2 | Q9Y2G5 | R324W |
| 56 | CYP11B2 | P19099 | S183N | 155 | POLG | P54098 | R964C |
| 57 | CYP11B2 | P19099 | L184M | 156 | PRKCA | P17252 | D652Y |
| 58 | CYP11B2 | P19099 | I192M | 157 | PRL | P01236 | R71W |
| 59 | DECR1 | Q16698 | E304K | 158 | PTK2 | Q05397 | R349W |
| 60 | DECR1 | Q16698 | R315I | 159 | PTK2 | Q05397 | H79Y |
| 61 | DOCK2 | Q92608 | F1328L | 160 | PTPN1 | P18031 | R257Q |
| 62 | DOHH | Q9BU89 | A39T | 161 | PTPN22 | Q9Y2R2 | R141C |
| 63 | DPEP1 | P16444 | Q361H | 162 | PTPN7 | P35236 | R327W |
| 64 | DPP10 | Q8N608 | M264V | 163 | PTPRR | Q15256 | R624C |
| 65 | DPP10 | Q8N608 | K427M | 164 | RALB | P11234 | E106K |
| 66 | DPP10 | Q8N608 | I656N | 165 | RALB | P11234 | E175K |
| 67 | DPP4 | P27487 | A732S | 166 | RBKS | Q9H477 | R168P |
| 68 | DPP4 | P27487 | R358M | 167 | RNASE2 | P10153 | R95C |
| 69 | DPP4 | P27487 | H750N | 168 | RNASEH1 | O60930 | P166S |
| 70 | DYNC2H1 | Q8NCM8 | L3038R | 169 | RORA | P35398 | S283L |
| 71 | EIF4E2 | O60573 | F97L | 170 | RP2 | O75695 | I166M |
| 72 | EPHA7 | Q15375 | E879K | 171 | RPS6KA3 | P51812 | L506I |
| 73 | EPHB1 | P54762 | Q720H | 172 | RPS6KA5 | O75582 | V711M |
| 74 | EPHB1 | P54762 | R865Q | 173 | RRM2B | Q7LG56 | R110C |
| 75 | EPHB1 | P54762 | R883Q | 174 | SENP8 | Q96LD8 | Y38F |
| 76 | EPM2A | O95278 | G271D | 175 | SMYD2 | Q9NRG4 | N300D |
| 77 | F13A1 | P00488 | K505E | 176 | SNRK | Q9NRH2 | A327T |
| 78 | F2 | P00734 | V525A | 177 | SRP54 | P61011 | Q51L |
| 79 | FBP2 | O00757 | R158H | 178 | ST8SIA3 | O43173 | R122W |
| 80 | FOXM1 | Q08050 | H269P | 179 | TF | P02787 | E231D |
| 81 | GAD1 | Q99259 | A253T | 180 | TKT | P29401 | I410M |
| 82 | GGCT | O75223 | S93Y | 181 | TPH2 | Q8IWU9 | R270S |
| 83 | GLO1 | Q04760 | K60N | 182 | TPH2 | Q8IWU9 | K331T |
| 84 | GOT1 | P17174 | R305Q | 183 | TPST2 | O60704 | R105H |
| 85 | GOT1 | P17174 | R42H | 184 | TRIM24 | O15164 | R910C |
| 86 | GRM5 | P41594 | H390Q | 185 | TTLL7 | Q6ZT98 | A374V |
| 87 | GRM7 | Q14831 | D202N | 186 | TUSC3 | Q13454 | R170I |
| 88 | GRM8 | O00222 | A408S | 187 | TYK2 | P29597 | P1105L |
| 89 | GRM8 | O00222 | N450H | 188 | UBE2G2 | P60604 | A26E |
| 90 | GRM8 | O00222 | N452K | 189 | UBE2T | Q9NPD8 | S101Y |
| 91 | GSTA2 | P09210 | I106L | 190 | UCHL1 | P09936 | V217M |
| 92 | GSTA2 | P09210 | P206H | 191 | UGDH | O60701 | R102L |
| 93 | HENMT1 | Q5T8I9 | M157I | 192 | USP5 | P45974 | F463L |
| 94 | HLA-G | P17693 | A235V | 193 | USP8 | P40818 | K851N |
| 95 | HNRNPF | P52597 | V113M | 194 | VRK1 | Q99986 | R160I |
| 96 | HNRNPF | P52597 | S161L | 195 | XRCC6 | P12956 | Q106R |
| 97 | HSPA1L | P34931 | R263G | 196 | YARS2 | Q9Y2Z4 | Y230N |
| 98 | HSPA2 | P54652 | D70N | 197 | YARS2 | Q9Y2Z4 | F229L |
| 99 | IDE | P14735 | H885L |  |  |  |  |

**Table S3.** Functional driver mutations in the functional dataset.

| Index | Gene name | Uniprot ID | Mutation | Index | Gene name | Uniprot ID | Mutation |
| --- | --- | --- | --- | --- | --- | --- | --- |
| 1 | ALK | Q9UM73 | L1122V | 38 | NRAS | P01111 | G13R |
| 2 | ALK | Q9UM73 | G1128A | 39 | PTEN | P60484 | C124R |
| 3 | ALK | Q9UM73 | I1171S | 40 | PTEN | P60484 | G127N |
| 4 | ALK | Q9UM73 | V1180L | 41 | PTEN | P60484 | G129D |
| 5 | ALK | Q9UM73 | M1328I | 42 | PTEN | P60484 | G129R |
| 6 | BTK | Q06187 | C481S | 43 | PTEN | P60484 | R130G |
| 7 | CDK4 | P11802 | K22A | 44 | PTEN | P60484 | G165E |
| 8 | EGFR | P00533 | G719C | 45 | PTEN | P60484 | T167A |
| 9 | EGFR | P00533 | G719S | 46 | PTEN | P60484 | R173H |
| 10 | EGFR | P00533 | T790M | 47 | PTEN | P60484 | F347L |
| 11 | EGFR | P00533 | C797S | 48 | PTEN | P60484 | M35R |
| 12 | EGFR | P00533 | V769M | 49 | PTEN | P60484 | G36R |
| 13 | EP300 | Q09472 | P1502L | 50 | PTEN | P60484 | C71Y |
| 14 | ERBB2 | P04626 | K753E | 51 | PTEN | P60484 | P96Q |
| 15 | ERBB2 | P04626 | L755S | 52 | PTEN | P60484 | L345Q |
| 16 | ERBB2 | P04626 | V773A | 53 | PTEN | P60484 | N276S |
| 17 | ERBB2 | P04626 | T798M | 54 | PTEN | P60484 | G251C |
| 18 | ERBB2 | P04626 | T862A | 55 | PTEN | P60484 | Y174N |
| 19 | ERBB3 | P21860 | V855A | 56 | RAC1 | P63000 | G12V |
| 20 | FGFR2 | P21802 | R678G | 57 | RAC1 | P63000 | P29L |
| 21 | FGFR2 | P21802 | K526E | 58 | RAC1 | P63000 | P29S |
| 22 | FGFR2 | P21802 | K659E | 59 | RAF1 | P04049 | S427G |
| 23 | FGFR4 | P22455 | N535K | 60 | ROS1 | P08922 | L1951R |
| 24 | FGFR4 | P22455 | V550E | 61 | ROS1 | P08922 | C2060G |
| 25 | FLT3 | P36888 | F691L | 62 | SETD2 | Q9BYW2 | R1625C |
| 26 | FLT3 | P36888 | R834Q | 63 | SMAD3 | P84022 | R373Q |
| 27 | GNAS | P63092 | R201C | 64 | TP53 | P04637 | L145R |
| 28 | GNAS | P63092 | R201H | 65 | TP53 | P04637 | Y220C |
| 29 | GSTP1 | P09211 | I105V | 66 | TP53 | P04637 | G244R |
| 30 | JAK1 | P23458 | F958C | 67 | TP53 | P04637 | G244S |
| 31 | MAP2K1 | Q02750 | P306H | 68 | TP53 | P04637 | G245S |
| 32 | MLH1 | P40692 | T117M | 69 | TP53 | P04637 | D186A |
| 33 | MLH1 | P40692 | G67R | 70 | TP53 | P04637 | P153H |
| 34 | MTOR | P42345 | L2230V | 71 | TP53 | P04637 | R110P |
| 35 | NRAS | P01111 | G12C | 72 | TP53 | P04637 | T150I |
| 36 | NRAS | P01111 | G12V | 73 | TP53 | P04637 | C229R |
| 37 | NRAS | P01111 | G13C |  |  |  |  |

**Table S4.** Functional passenger mutations in the functional dataset.

| Index | Gene name | Uniprot ID | Mutation | Index | Gene name | Uniprot ID | Mutation |
| --- | --- | --- | --- | --- | --- | --- | --- |
| 1 | A2M | P01023 | I1091V | 292 | GRM8 | O00222 | A408S |
| 2 | A2M | P01023 | R117W | 293 | GRM8 | O00222 | N450H |
| 3 | A2M | P01023 | P631T | 294 | GRM8 | O00222 | N452K |
| 4 | ACAA1 | P09110 | R249H | 295 | GSTA1 | P08263 | R131C |
| 5 | ACADM | P11310 | S127F | 296 | GSTA2 | P09210 | I106L |
| 6 | ACADS | P16219 | R330H | 297 | GSTA2 | P09210 | P206H |
| 7 | ACADVL | P49748 | L348P | 298 | GSTA2 | P09210 | I219V |
| 8 | ACAT1 | P24752 | Q211R | 299 | GSTM2 | P28161 | R108H |
| 9 | ACHE | P22303 | T467M | 300 | GZMH | P20718 | G178C |
| 10 | ACHE | P22303 | P477S | 301 | HAAO | P46952 | G51D |
| 11 | ACLY | P53396 | Q115L | 302 | HBE1 | P02100 | T39N |
| 12 | ACMSD | Q8TDX5 | D270E | 303 | HBE1 | P02100 | K66R |
| 13 | ACVRL1 | P37023 | V380G | 304 | HBE1 | P02100 | H93R |
| 14 | ACYP1 | P07311 | S93L | 305 | HBG2 | P69892 | V99M |
| 15 | ADA | P00813 | A215V | 306 | HCK | P08631 | A403T |
| 16 | ADAM8 | P78325 | D263N | 307 | HENMT1 | Q5T8I9 | M157I |
| 17 | ADAT2 | Q7Z6V5 | E38K | 308 | HEXA | P06865 | H204L |
| 18 | ADH1A | P07327 | E69V | 309 | HEXB | P07686 | L488V |
| 19 | ADH1B | P00325 | K19N | 310 | HLA-G | P17693 | A235V |
| 20 | ADH5 | P11766 | V203A | 311 | HMGCL | P35914 | R41Q |
| 21 | ADRB2 | P07550 | A200V | 312 | HMGCL | P35914 | S78Y |
| 22 | ADSL | P30566 | C304Y | 313 | HMGCS1 | Q01581 | D43Y |
| 23 | AGXT | P21549 | A112V | 314 | HMOX2 | P30519 | H152R |
| 24 | AGXT | P21549 | S205L | 315 | HNRNPF | P52597 | V113M |
| 25 | AGXT | P21549 | W332C | 316 | HNRNPF | P52597 | S161L |
| 26 | AHCY | P23526 | C421Y | 317 | HNRNPF | P52597 | M2I |
| 27 | AHCY | P23526 | P427L | 318 | HSP90B1 | P14625 | I105M |
| 28 | AHCYL1 | O43865 | D280N | 319 | HSPA1L | P34931 | R263G |
| 29 | AHCYL1 | O43865 | C292W | 320 | HSPA2 | P54652 | D70N |
| 30 | AHCYL2 | Q96HN2 | D582N | 321 | HSPA6 | P17066 | V148L |
| 31 | AK4 | P27144 | R170S | 322 | HSPA6 | P17066 | F152C |
| 32 | AK4 | P27144 | S187N | 323 | HSPA6 | P17066 | D236N |
| 33 | AKR1B1 | P15121 | D309E | 324 | HSPA6 | P17066 | R271C |
| 34 | AKR1C3 | P42330 | S87C | 325 | HTR1B | P28222 | F353L |
| 35 | AKR1C4 | P17516 | L235I | 326 | IDE | P14735 | H885L |
| 36 | AKR1C4 | P17516 | T251P | 327 | IFIT1 | P09914 | M377I |
| 37 | ALB | P02768 | R138Q | 328 | IL18RAP | O95256 | L166F |
| 38 | ALB | P02768 | E155V | 329 | IL18RAP | O95256 | D246N |
| 39 | ALB | P02768 | K543E | 330 | IL1RL1 | Q01638 | P114T |
| 40 | ALB | P02768 | A602S | 331 | IL1RL1 | Q01638 | V316I |
| 41 | ALDH1A1 | P00352 | F425L | 332 | IL22RA2 | Q969J5 | K189N |
| 42 | ALDH4A1 | P30038 | G521V | 333 | ILK | Q13418 | K448N |
| 43 | ALDH4A1 | P30038 | G521W | 334 | IMPA2 | O14732 | D255N |
| 44 | ALDH7A1 | P49419 | G219E | 335 | INSR | P06213 | V1054M |
| 45 | ALDOA | P04075 | R60L | 336 | INSR | P06213 | G1211R |
| 46 | ALDOB | P05062 | Y204H | 337 | INSR | P06213 | S1241C |
| 47 | ALDOB | P05062 | T260N | 338 | KARS | Q15046 | A280T |
| 48 | ALOX12 | P18054 | G454E | 339 | KAT7 | O95251 | A425T |
| 49 | ALOX5 | P09917 | A607V | 340 | KCNK1 | O00180 | G121D |
| 50 | AMD1 | P17707 | S229L | 341 | KCNK1 | O00180 | L146V |
| 51 | AMD1 | P17707 | M70R | 342 | KDM6B | O15054 | W1410C |
| 52 | AMT | P48728 | T115I | 343 | KIF22 | Q14807 | Y126C |
| 53 | AMY2A | P04746 | R176H | 344 | KIF2C | Q99661 | I259T |
| 54 | AMY2A | P04746 | M217T | 345 | KIF3B | O15066 | R336H |
| 55 | AMY2A | P04746 | R76I | 346 | KLK10 | O43240 | A85T |
| 56 | ANPEP | P15144 | E636K | 347 | KLK10 | O43240 | C87S |
| 57 | ANXA5 | P08758 | D20N | 348 | LCK | P06239 | H208N |
| 58 | ANXA5 | P08758 | R25W | 349 | LCK | P06239 | C217Y |
| 59 | AOC1 | P19801 | N110K | 350 | LDHB | P07195 | S168Y |
| 60 | AOC1 | P19801 | Q353E | 351 | LDHB | P07195 | T249A |
| 61 | AOC3 | Q16853 | A456V | 352 | LGR5 | O75473 | Q122H |
| 62 | AOC3 | Q16853 | E556Q | 353 | LIG1 | P18858 | E566K |
| 63 | AOX1 | Q06278 | H1052Q | 354 | LILRB1 | Q8NHL6 | N203K |
| 64 | AOX1 | Q06278 | L1094I | 355 | LIPF | P07098 | S300Y |
| 65 | AOX1 | Q06278 | R833C | 356 | LIPF | P07098 | G340C |
| 66 | APOBEC3A | P31941 | I96M | 357 | LTF | P02788 | A550V |
| 67 | APRT | P07741 | R87Q | 358 | LYPLA1 | O75608 | S7L |
| 68 | ARHGDIB | P52566 | E190Q | 359 | MAP3K12 | Q12852 | S253L |
| 69 | ARHGDIB | P52566 | E37K | 360 | MAP3K7 | O43318 | K52N |
| 70 | ARL1 | P40616 | Y35C | 361 | MAP3K9 | P80192 | R160H |
| 71 | ARL1 | P40616 | R99Q | 362 | MAPK10 | P53779 | L393R |
| 72 | ARL2 | P36404 | D42N | 363 | MAPK10 | P53779 | A80V |
| 73 | ASL | P04424 | E399D | 364 | MAPK8 | P45983 | L115I |
| 74 | ASMT | P46597 | R153L | 365 | MAPK8 | P45983 | K166N |
| 75 | ASMT | P46597 | E234K | 366 | MAPK9 | P45984 | Q37E |
| 76 | ASPA | P45381 | D114H | 367 | MARK3 | P27448 | S215C |
| 77 | ASPA | P45381 | L115I | 368 | MASP2 | O00187 | Y410N |
| 78 | ASPH | Q12797 | K336T | 369 | MAT1A | Q00266 | P255S |
| 79 | ASPH | Q12797 | V676M | 370 | MBD4 | O95243 | K565N |
| 80 | ASPH | Q12797 | V740M | 371 | MCL1 | Q07820 | F270S |
| 81 | ASRGL1 | Q7L266 | G214E | 372 | MCPH1 | Q8NEM0 | E681K |
| 82 | ASRGL1 | Q7L266 | A94T | 373 | MCPH1 | Q8NEM0 | R791H |
| 83 | ATXN3 | P54252 | E43Q | 374 | MDH2 | P40926 | P87S |
| 84 | BACE1 | P56817 | S71L | 375 | ME2 | P23368 | R165I |
| 85 | BCHE | P06276 | S226T | 376 | ME2 | P23368 | E255K |
| 86 | BCHE | P06276 | L301I | 377 | MECR | Q9BV79 | I301M |
| 87 | BHMT | Q93088 | D263N | 378 | MEPCE | Q7L2J0 | R443W |
| 88 | BMX | P51813 | E422Q | 379 | MMAA | Q8IVH4 | V220L |
| 89 | BPGM | P07738 | H188D | 380 | MMAA | Q8IVH4 | R316C |
| 90 | BST1 | Q10588 | A138T | 381 | MME | P08473 | R293Q |
| 91 | C5AR1 | P21730 | R178Q | 382 | MME | P08473 | S548T |
| 92 | C5AR1 | P21730 | D195N | 383 | MME | P08473 | R748W |
| 93 | C5AR1 | P21730 | A88V | 384 | MMP12 | P39900 | G178C |
| 94 | C5AR1 | P21730 | S95P | 385 | MMP16 | P51512 | D239Y |
| 95 | CA12 | O43570 | P73H | 386 | MMP16 | P51512 | Q269L |
| 96 | CA13 | Q8N1Q1 | R81H | 387 | MPG | P29372 | E133A |
| 97 | CA2 | P00918 | E117K | 388 | MPG | P29372 | G189D |
| 98 | CA4 | P22748 | T226I | 389 | MPO | P05164 | L333F |
| 99 | CA4 | P22748 | P39A | 390 | MPO | P05164 | Q696H |
| 100 | CA6 | P23280 | W230L | 391 | MPO | P05164 | E411K |
| 101 | CAMK2D | Q13557 | C127F | 392 | MPO | P05164 | G451W |
| 102 | CAMKK2 | Q96RR4 | P431S | 393 | MPO | P05164 | R590H |
| 103 | CASK | O14936 | E92K | 394 | MVK | Q03426 | I196V |
| 104 | CASP2 | P42575 | R321H | 395 | MYOC | Q99972 | Y437C |
| 105 | CASP3 | P42574 | G165D | 396 | NAMPT | P43490 | K229T |
| 106 | CASP7 | P55210 | S231L | 397 | NCS1 | P62166 | F82L |
| 107 | CASP7 | P55210 | R87Q | 398 | NEK2 | P51955 | R69C |
| 108 | CBS | P35520 | D234N | 399 | NFYA | P23511 | A309T |
| 109 | CD1A | P06126 | L165I | 400 | NFYC | Q13952 | D116N |
| 110 | CD1A | P06126 | A33T | 401 | NME1 | P15531 | E129V |
| 111 | CD1A | P06126 | R99G | 402 | NME3 | Q13232 | R122H |
| 112 | CD1A | P06126 | R99H | 403 | NOS1 | P29475 | E312K |
| 113 | CD1D | P15813 | Q137E | 404 | NOS1 | P29475 | R419C |
| 114 | CD1D | P15813 | L142M | 405 | NOS1 | P29475 | R419H |
| 115 | CD1D | P15813 | G46S | 406 | NOS1 | P29475 | Q483P |
| 116 | CD38 | P28907 | R66Q | 407 | NOS1 | P29475 | R486C |
| 117 | CD38 | P28907 | D217G | 408 | NOS1 | P29475 | V572M |
| 118 | CD38 | P28907 | G223W | 409 | NOS2 | P35228 | A351V |
| 119 | CD38 | P28907 | R66P | 410 | NOS3 | P29474 | A332P |
| 120 | CDA | P32320 | M106I | 411 | NOS3 | P29474 | A332T |
| 121 | CDA | P32320 | C53R | 412 | NOTUM | Q6P988 | G230R |
| 122 | CDA | P32320 | P61Q | 413 | NR1H3 | Q13133 | A255T |
| 123 | CDA | P32320 | R68L | 414 | NR5A2 | O00482 | D389N |
| 124 | CDA | P32320 | M91V | 415 | NR5A2 | O00482 | H430R |
| 125 | CDC34 | P49427 | T122A | 416 | NR5A2 | O00482 | N523T |
| 126 | CDC42 | P60953 | D11N | 417 | OCRL | Q01968 | R552Q |
| 127 | CDC42 | P60953 | D57G | 418 | ODC1 | P11926 | D364N |
| 128 | CDC7 | O00311 | I132T | 419 | OTC | P00480 | R92Q |
| 129 | CDC7 | O00311 | H175N | 420 | OTUB2 | Q96DC9 | K73T |
| 130 | CDK7 | P50613 | E20K | 421 | P4HB | P07237 | A118G |
| 131 | CDK9 | P50750 | A152S | 422 | PAICS | P22234 | K283T |
| 132 | CDKL1 | Q00532 | H125Y | 423 | PAPSS2 | O95340 | L50I |
| 133 | CDKL1 | Q00532 | Y16H | 424 | PASK | Q96RG2 | F1076L |
| 134 | CDKL1 | Q00532 | V19A | 425 | PC | P11498 | P524H |
| 135 | CDKL1 | Q00532 | F36L | 426 | PCK1 | P35558 | A283S |
| 136 | CDKL5 | O76039 | L141I | 427 | PCK1 | P35558 | P285L |
| 137 | CES1 | P23141 | S305P | 428 | PDE1B | Q01064 | P221L |
| 138 | CES1 | P23141 | S365T | 429 | PDE1B | Q01064 | H263L |
| 139 | CETP | P11597 | Q141L | 430 | PDE3B | Q13370 | R694T |
| 140 | CETP | P11597 | D156N | 431 | PDE3B | Q13370 | D846Y |
| 141 | CETP | P11597 | A51T | 432 | PDE3B | Q13370 | R881H |
| 142 | CHAT | P28329 | S214C | 433 | PDE9A | O76083 | D353N |
| 143 | CHIT1 | Q13231 | D138Y | 434 | PDE9A | O76083 | A510V |
| 144 | CHRM2 | P08172 | S110P | 435 | PDHA1 | P08559 | Q84H |
| 145 | CHRM2 | P08172 | S151T | 436 | PDK4 | Q16654 | R161Q |
| 146 | CHRM2 | P08172 | A401V | 437 | PEX5 | P50542 | M613I |
| 147 | CISD3 | P0C7P0 | K48T | 438 | PFKFB2 | O60825 | I101M |
| 148 | CISD3 | P0C7P0 | T80I | 439 | PFKFB2 | O60825 | R102H |
| 149 | CLK2 | P49760 | G170V | 440 | PFKFB2 | O60825 | R193I |
| 150 | CNR1 | P21554 | T197M | 441 | PFKP | Q01813 | D128N |
| 151 | CNR1 | P21554 | Y275S | 442 | PGC | P20142 | P50A |
| 152 | CNR1 | P21554 | I280L | 443 | PGD | P52209 | E436Q |
| 153 | CP | P00450 | A530S | 444 | PGK1 | P00558 | N195S |
| 154 | CP | P00450 | L666M | 445 | PGM1 | P36871 | R23Q |
| 155 | CPA2 | P48052 | R386H | 446 | PHGDH | O43175 | T125M |
| 156 | CPB1 | P15086 | F175C | 447 | PHYH | O14832 | R275W |
| 157 | CPS1 | P31327 | R1262Q | 448 | PHYH | O14832 | K59N |
| 158 | CPS1 | P31327 | D1277Y | 449 | PIK3CG | P48736 | V162I |
| 159 | CPS1 | P31327 | V584M | 450 | PIK3CG | P48736 | R690I |
| 160 | CPS1 | P31327 | E631Q | 451 | PIP4K2A | P48426 | S48W |
| 161 | CPS1 | P31327 | R721L | 452 | PIP4K2B | P78356 | K214T |
| 162 | CPS1 | P31327 | R721Q | 453 | PIP4K2B | P78356 | M65V |
| 163 | CSAD | Q9Y600 | E45Q | 454 | PIP4K2C | Q8TBX8 | P131L |
| 164 | CSAD | Q9Y600 | R380H | 455 | PITPNA | Q00169 | K110E |
| 165 | CSK | P41240 | R318C | 456 | PITPNA | Q00169 | I61V |
| 166 | CTBP1 | Q13363 | R97W | 457 | PKM | P14618 | R342W |
| 167 | CTH | P32929 | E345K | 458 | PLA2G7 | Q13093 | L111P |
| 168 | CTH | P32929 | A357V | 459 | PLCB2 | Q00722 | S406L |
| 169 | CTH | P32929 | R375Q | 460 | PLK1 | P53350 | R57S |
| 170 | CTPS1 | P17812 | S156G | 461 | PMP2 | P02689 | N35D |
| 171 | CTPS1 | P17812 | G15E | 462 | PMP2 | P02689 | Q96K |
| 172 | CTSB | P07858 | V191I | 463 | PNLIPRP2 | P54317 | F149L |
| 173 | CTSD | P07339 | R266C | 464 | PNP | P00491 | A216T |
| 174 | CTSE | P14091 | D96N | 465 | PNP | P00491 | L240F |
| 175 | CTSL | P07711 | G252V | 466 | POFUT2 | Q9Y2G5 | R324W |
| 176 | CTSV | O60911 | M250V | 467 | POLG | P54098 | R964C |
| 177 | CYCS | P99999 | N55D | 468 | POLRMT | O00411 | R860C |
| 178 | CYP11A1 | P05108 | L140Q | 469 | POU6F1 | Q14863 | F282Y |
| 179 | CYP11B2 | P19099 | S183N | 470 | POU6F1 | Q14863 | R285Q |
| 180 | CYP11B2 | P19099 | L184M | 471 | PPOX | P50336 | R168H |
| 181 | CYP11B2 | P19099 | I192M | 472 | PPP3CB | P16298 | G158D |
| 182 | CYP11B2 | P19099 | S308Y | 473 | PRKCA | P17252 | D652Y |
| 183 | CYP19A1 | P11511 | I217T | 474 | PRKG2 | Q13237 | E318K |
| 184 | CYP19A1 | P11511 | A306T | 475 | PRL | P01236 | H208N |
| 185 | CYP1A1 | P04798 | L114I | 476 | PRL | P01236 | K215R |
| 186 | CYP1A1 | P04798 | E226D | 477 | PRL | P01236 | R220Q |
| 187 | CYP2C19 | P33261 | A292T | 478 | PRL | P01236 | I221L |
| 188 | CYP2C8 | P10632 | S478C | 479 | PRL | P01236 | R71W |
| 189 | CYP2C9 | P11712 | T301M | 480 | PROC | P04070 | V434I |
| 190 | CYP7A1 | P22680 | R260C | 481 | PTK2 | Q05397 | R349W |
| 191 | DAO | P14920 | I138L | 482 | PTK2 | Q05397 | H79Y |
| 192 | DAO | P14920 | Q201H | 483 | PTPN1 | P18031 | R257Q |
| 193 | DAO | P14920 | T216I | 484 | PTPN22 | Q9Y2R2 | R141C |
| 194 | DAO | P14920 | T216P | 485 | PTPN3 | P26045 | T744M |
| 195 | DAO | P14920 | R283Q | 486 | PTPN6 | P29350 | R495H |
| 196 | DAPK1 | P53355 | E100K | 487 | PTPN7 | P35236 | R327W |
| 197 | DAPK1 | P53355 | A25V | 488 | PTPN9 | P43378 | G334R |
| 198 | DCK | P27707 | A56V | 489 | PTPRR | Q15256 | R624C |
| 199 | DECR1 | Q16698 | E304K | 490 | PTPRS | Q13332 | T1491M |
| 200 | DECR1 | Q16698 | R315I | 491 | PUDP | Q08623 | A112V |
| 201 | DHODH | Q02127 | S150R | 492 | PYGB | P11216 | R650H |
| 202 | DHPS | P49366 | A343T | 493 | PYGM | P11217 | D694V |
| 203 | DOCK2 | Q92608 | F1328L | 494 | RAB10 | P61026 | I39F |
| 204 | DOHH | Q9BU89 | A39T | 495 | RAB11A | P62491 | L128V |
| 205 | DPEP1 | P16444 | Q361H | 496 | RAB6A | P20340 | F18Y |
| 206 | DPP10 | Q8N608 | M264V | 497 | RAD52 | P43351 | D123N |
| 207 | DPP10 | Q8N608 | K427M | 498 | RAD52 | P43351 | G163V |
| 208 | DPP10 | Q8N608 | I656N | 499 | RALB | P11234 | E106K |
| 209 | DPP4 | P27487 | A732S | 500 | RALB | P11234 | E175K |
| 210 | DPP4 | P27487 | R358M | 501 | RAN | P62826 | K127T |
| 211 | DPP4 | P27487 | A654V | 502 | RAP1A | P62834 | S150L |
| 212 | DPP4 | P27487 | H750N | 503 | RAP1A | P62834 | V154I |
| 213 | DPP6 | P42658 | D629N | 504 | RAP1A | P62834 | S83A |
| 214 | DUT | P33316 | V197A | 505 | RAP1B | P61224 | S83F |
| 215 | DYNC2H1 | Q8NCM8 | L3038R | 506 | RAP2A | P10114 | D38N |
| 216 | ECE1 | P42892 | S150N | 507 | RBKS | Q9H477 | R168P |
| 217 | ECI1 | P42126 | E300D | 508 | RBP1 | P09455 | W107C |
| 218 | EFNB2 | P52799 | F110I | 509 | RBP1 | P09455 | R121T |
| 219 | EIF4E2 | O60573 | F97L | 510 | REN | P00797 | R148C |
| 220 | ELAVL4 | P26378 | T160A | 511 | RIPK2 | O43353 | H144Y |
| 221 | ENPEP | Q07075 | T366M | 512 | RNASE2 | P10153 | R95C |
| 222 | ENPEP | Q07075 | R386S | 513 | RNASE3 | P12724 | T33M |
| 223 | ENPP2 | Q13822 | F169L | 514 | RNASE3 | P12724 | R63H |
| 224 | ENPP2 | Q13822 | H243R | 515 | RNASE4 | P34096 | T107I |
| 225 | EPHA7 | Q15375 | E879K | 516 | RNASE4 | P34096 | R130C |
| 226 | EPHB1 | P54762 | Q720H | 517 | RNASEH1 | O60930 | P166S |
| 227 | EPHB1 | P54762 | R865Q | 518 | RNASEH2A | O75792 | E35V |
| 228 | EPHB1 | P54762 | R883Q | 519 | RORA | P35398 | S283L |
| 229 | EPHX2 | P34913 | S407R | 520 | RORC | P51449 | T375M |
| 230 | EPM2A | O95278 | G271D | 521 | RP2 | O75695 | I166M |
| 231 | EPRS | P07814 | R1119H | 522 | RPS6KA3 | P51812 | L209F |
| 232 | EPRS | P07814 | Y1127C | 523 | RPS6KA3 | P51812 | L506I |
| 233 | ERI3 | O43414 | L203F | 524 | RPS6KA5 | O75582 | G433R |
| 234 | ETHE1 | O95571 | T78S | 525 | RPS6KA5 | O75582 | V711M |
| 235 | F10 | P00742 | M402I | 526 | RRM2B | Q7LG56 | R110C |
| 236 | F13A1 | P00488 | K505E | 527 | SAT1 | P21673 | E23K |
| 237 | F2 | P00734 | V525A | 528 | SCO2 | O43819 | N210S |
| 238 | F9 | P00740 | Y444H | 529 | SDS | P20132 | L172M |
| 239 | FABP2 | P12104 | D75V | 530 | SEC14L2 | O76054 | K165Q |
| 240 | FABP7 | O15540 | R127C | 531 | SENP8 | Q96LD8 | Y38F |
| 241 | FABP7 | O15540 | R127H | 532 | SEPT2 | Q15019 | S51L |
| 242 | FBP2 | O00757 | R158H | 533 | SERPINA6 | P08185 | M255I |
| 243 | FBP2 | O00757 | S271I | 534 | SERPINA6 | P08185 | S394R |
| 244 | FDPS | P14324 | R178C | 535 | SHBG | P04278 | H112D |
| 245 | FECH | P22830 | R115Q | 536 | SI | P14410 | R263Q |
| 246 | FECH | P22830 | Q122K | 537 | SLC1A3 | P43003 | R479W |
| 247 | FGF10 | O15520 | K87N | 538 | SLC2A3 | P11169 | T319N |
| 248 | FOLH1 | Q04609 | G256R | 539 | SLC6A4 | P31645 | E444Q |
| 249 | FOLH1 | Q04609 | E424D | 540 | SLC6A4 | P31645 | A496V |
| 250 | FOLH1 | Q04609 | R580Q | 541 | SMYD2 | Q9NRG4 | N300D |
| 251 | FOXM1 | Q08050 | H269P | 542 | SNRK | Q9NRH2 | A327T |
| 252 | FOXN1 | O15353 | S277R | 543 | SRP54 | P61011 | Q51L |
| 253 | FTL | P02792 | E58D | 544 | ST6GAL1 | P15907 | W96R |
| 254 | FZD7 | O75084 | H73D | 545 | ST8SIA3 | O43173 | R122W |
| 255 | GAD1 | Q99259 | A253T | 546 | ST8SIA3 | O43173 | S168G |
| 256 | GAD2 | Q05329 | S284Y | 547 | ST8SIA3 | O43173 | R298W |
| 257 | GAD2 | Q05329 | R558C | 548 | STAT6 | P42226 | K284N |
| 258 | GALK1 | P51570 | T344M | 549 | STK25 | O00506 | R25H |
| 259 | GALK1 | P51570 | A82S | 550 | SULT1C4 | O75897 | H83Y |
| 260 | GALK2 | Q01415 | F111L | 551 | TARS | P26639 | R591Q |
| 261 | GALK2 | Q01415 | E231A | 552 | TF | P02787 | E231D |
| 262 | GARS | P41250 | D468Y | 553 | TFRC | P02786 | N527Y |
| 263 | GBE1 | Q04446 | A247V | 554 | TGM2 | P21980 | S250I |
| 264 | GGCT | O75223 | S93Y | 555 | TKT | P29401 | I410M |
| 265 | GGPS1 | O95749 | G157E | 556 | TPH2 | Q8IWU9 | R270S |
| 266 | GGPS1 | O95749 | K212N | 557 | TPH2 | Q8IWU9 | K331T |
| 267 | GGT1 | P19440 | E108K | 558 | TPST2 | O60704 | R105H |
| 268 | GLO1 | Q04760 | K60N | 559 | TPST2 | O60704 | R183W |
| 269 | GLO1 | Q04760 | A92V | 560 | TRDMT1 | O14717 | R371H |
| 270 | GLUD1 | P00367 | Y439H | 561 | TRIM24 | O15164 | R910C |
| 271 | GLUL | P15104 | R319C | 562 | TSTA3 | Q13630 | I150S |
| 272 | GLUL | P15104 | D63N | 563 | TTLL7 | Q6ZT98 | A374V |
| 273 | GMDS | O60547 | G241R | 564 | TUSC3 | Q13454 | R170I |
| 274 | GNAI3 | P08754 | R205I | 565 | TYK2 | P29597 | P1105L |
| 275 | GOT1 | P17174 | R305Q | 566 | UBE2G2 | P60604 | A26E |
| 276 | GOT1 | P17174 | R387Q | 567 | UBE2K | P61086 | D98G |
| 277 | GOT1 | P17174 | R42H | 568 | UBE2T | Q9NPD8 | S101Y |
| 278 | GPI | P06744 | K211M | 569 | UCHL1 | P09936 | D156Y |
| 279 | GPI | P06744 | T218S | 570 | UCHL1 | P09936 | V217M |
| 280 | GRIA2 | P42262 | M484I | 571 | UGDH | O60701 | R102L |
| 281 | GRIA2 | P42262 | F679L | 572 | ULK1 | O75385 | A28V |
| 282 | GRIK1 | P39086 | A699V | 573 | UMPS | P11172 | K29T |
| 283 | GRIN2A | Q12879 | S511L | 574 | USP5 | P45974 | F463L |
| 284 | GRM3 | Q14832 | V191M | 575 | USP8 | P40818 | K851N |
| 285 | GRM3 | Q14832 | R277H | 576 | VCP | P55072 | D686G |
| 286 | GRM5 | P41594 | V192L | 577 | VNN1 | O95497 | K273E |
| 287 | GRM5 | P41594 | F277V | 578 | VRK1 | Q99986 | R160I |
| 288 | GRM5 | P41594 | H390Q | 579 | XDH | P47989 | R149H |
| 289 | GRM7 | Q14831 | E185G | 580 | XRCC6 | P12956 | Q106R |
| 290 | GRM7 | Q14831 | D202N | 581 | YARS2 | Q9Y2Z4 | Y230N |
| 291 | GRM8 | O00222 | G312V | 582 | YARS2 | Q9Y2Z4 | F229L |

**Table S5.** Summary of retrieved driver mutations in benchmarking test datasets

| Mutation type | Gene role | Gene name^a^ | # of driver mutations | # of retrieved mutations |
| --- | --- | --- | --- | --- |
| Allosteric driver mutation | Oncogene | ALK | 1 | 1 |
|  |  | EGFR | 1 | 1 |
|  |  | FGFR2 | 3 | 2 |
|  |  | MAP2K1 | 1 | 1 |
|  |  | MTOR | 1 | 1 |
|  |  | ROS1 | 1 | 1 |
|  | Tumor suppressor | EP300 | 1 | 1 |
|  |  | PTEN | 7 | 6 |
|  |  | SMAD3 | 1 | 1 |
|  |  | TP53 | 7 | 7 |
| Orthosteric driver mutation | Oncogene | ALK | 4 | 4 |
|  |  | BTK | 1 | 1 |
|  |  | CDK4 | 1 | 1 |
|  |  | EGFR | 4 | 3 |
|  |  | ERBB2 | 5 | 3 |
|  |  | ERBB3 | 1 | 1 |
|  |  | FGFR4 | 2 | 2 |
|  |  | FLT3 | 2 | 2 |
|  |  | GNAS | 2 | 1 |
|  |  | GSTP1 | 1 | 1 |
|  |  | JAK1 | 1 | 1 |
|  |  | NRAS | 4 | 4 |
|  |  | RAC1 | 3 | 3 |
|  |  | RAF1 | 1 | 1 |
|  |  | ROS1 | 1 | 1 |
|  | Tumor suppressor | MLH1 | 2 | 2 |
|  |  | PTEN | 10 | 5 |
|  |  | SETD2 | 1 | 1 |
|  |  | TP53 | 3 | 2 |

^a^Abbreviations: ALK, ALK tyrosine kinase receptor; BTK, Tyrosine-protein kinase BTK; CDK4, Cyclin-dependent kinase 4; EGFR, Epidermal growth factor receptor; EP300, Histone acetyltransferase p300; ERBB2, Receptor tyrosine-protein kinase erbB-2; ERBB3, Receptor tyrosine-protein kinase erbB-3; FGFR2, Fibroblast growth factor receptor 2; FGFR4, Fibroblast growth factor receptor 4; FLT3, Receptor-type tyrosine-protein kinase FLT3; GNAS, Guanine nucleotide-binding protein G(s) subunit alpha isoforms short; GSTP1, Glutathione S-transferase P; JAK1, Tyrosine-protein kinase JAK1; MAP2K1, Dual specificity mitogen-activated protein kinase kinase 1; MLH1, DNA mismatch repair protein Mlh1; MTOR, Serine/threonine-protein kinase mTOR; NRAS, GTPase NRas; PIK3CA, Phosphatidylinositol 4,5-bisphosphate 3-kinase catalytic subunit alpha isoform; PTEN, Phosphatidylinositol 3,4,5-trisphosphate 3-phosphatase and dual-specificity protein phosphatase PTEN; RAC1, Ras-related C3 botulinum toxin substrate 1; RAF1, RAF proto-oncogene serine/threonine-protein kinase; ROS1, Proto-oncogene tyrosine-protein kinase ROS; SETD2, Histone-lysine N-methyltransferase SETD2; SMAD3, Mothers against decapentaplegic homolog 3; TP53, Cellular tumor antigen p53

**Table S6.** Driver mutations in the training dataset.

| Index | Gene name | Uniprot ID | Mutation | Index | Gene name | Uniprot ID | Mutation |
| --- | --- | --- | --- | --- | --- | --- | --- |
| 1 | ABL1 | P00519 | E255V | 180 | HRAS | P01112 | K117N |
| 2 | ABL1 | P00519 | E255K | 181 | HRAS | P01112 | K117R |
| 3 | ABL1 | P00519 | F359V | 182 | HRAS | P01112 | T58I |
| 4 | ABL1 | P00519 | F359L | 183 | HRAS | P01112 | Q61E |
| 5 | ABL1 | P00519 | F359I | 184 | HRAS | P01112 | Q61L |
| 6 | ABL1 | P00519 | F359C | 185 | HRAS | P01112 | Q61H |
| 7 | ABL1 | P00519 | Q252H | 186 | HRAS | P01112 | Q61K |
| 8 | ABL1 | P00519 | D363Y | 187 | HRAS | P01112 | Q61P |
| 9 | ABL1 | P00519 | V289F | 188 | HRAS | P01112 | Q61R |
| 10 | ABL1 | P00519 | V289A | 189 | HRAS | P01112 | G12V |
| 11 | ABL1 | P00519 | F382L | 190 | HRAS | P01112 | G12R |
| 12 | ABL1 | P00519 | V289I | 191 | HRAS | P01112 | G12S |
| 13 | ABL1 | P00519 | V299L | 192 | HRAS | P01112 | G12D |
| 14 | ABL1 | P00519 | V379I | 193 | HRAS | P01112 | G12C |
| 15 | ABL1 | P00519 | Y342H | 194 | HRAS | P01112 | G12A |
| 16 | ABL1 | P00519 | F283V | 195 | IDH1 | O75874 | R109K |
| 17 | ABL1 | P00519 | I293V | 196 | IDH1 | O75874 | R132Q |
| 18 | ABL1 | P00519 | Y320C | 197 | IDH1 | O75874 | R132P |
| 19 | ABL1 | P00519 | Y253F | 198 | IDH1 | O75874 | R132S |
| 20 | ABL1 | P00519 | Y253H | 199 | IDH1 | O75874 | R132H |
| 21 | ABL1 | P00519 | L298V | 200 | IDH1 | O75874 | R132L |
| 22 | ABL1 | P00519 | A380T | 201 | IDH1 | O75874 | R132C |
| 23 | ABL1 | P00519 | M343T | 202 | IDH1 | O75874 | R132G |
| 24 | ABL1 | P00519 | E258D | 203 | IDH1 | O75874 | M291I |
| 25 | ABL1 | P00519 | V371A | 204 | IDH1 | O75874 | G123R |
| 26 | ABL1 | P00519 | Q252R | 205 | IDH1 | O75874 | R100A |
| 27 | ABL1 | P00519 | V371L | 206 | IDH1 | O75874 | I130M |
| 28 | ABL1 | P00519 | A344V | 207 | IDH1 | O75874 | C379G |
| 29 | ABL1 | P00519 | L370P | 208 | IDH1 | O75874 | Y139D |
| 30 | ABL1 | P00519 | A433T | 209 | IDH1 | O75874 | G97D |
| 31 | ABL1 | P00519 | M472I | 210 | JAK2 | O60674 | G858S |
| 32 | ABL1 | P00519 | D381Y | 211 | JAK2 | O60674 | L551P |
| 33 | ABL1 | P00519 | F317C | 212 | JAK2 | O60674 | Y931C |
| 34 | ABL1 | P00519 | F317L | 213 | JAK2 | O60674 | L611V |
| 35 | ABL1 | P00519 | F317I | 214 | JAK2 | O60674 | L579P |
| 36 | ABL1 | P00519 | F317V | 215 | JAK2 | O60674 | L983F |
| 37 | ABL1 | P00519 | T315I | 216 | KDR | P35968 | S1037L |
| 38 | ABL1 | P00519 | T315A | 217 | KDR | P35968 | P351S |
| 39 | ABL1 | P00519 | L248V | 218 | KDR | P35968 | G345K |
| 40 | ABL1 | P00519 | G303R | 219 | KDR | P35968 | T864K |
| 41 | AKT1 | P31749 | Y176F | 220 | KDR | P35968 | R1051Q |
| 42 | AKT1 | P31749 | E17K | 221 | KDR | P35968 | G922E |
| 43 | AKT1 | P31749 | K179M | 222 | KRAS | P01116 | V152A |
| 44 | AKT1 | P31749 | E267G | 223 | KRAS | P01116 | N116H |
| 45 | AKT1 | P31749 | L202F | 224 | KRAS | P01116 | T20R |
| 46 | AKT1 | P31749 | V271A | 225 | KRAS | P01116 | G13E |
| 47 | AKT1 | P31749 | E191A | 226 | KRAS | P01116 | T58I |
| 48 | AKT1 | P31749 | N53A | 227 | KRAS | P01116 | Q61E |
| 49 | AKT1 | P31749 | V270A | 228 | KRAS | P01116 | Q61L |
| 50 | AKT1 | P31749 | Q79K | 229 | KRAS | P01116 | Q61H |
| 51 | AKT1 | P31749 | Q79E | 230 | KRAS | P01116 | G60C |
| 52 | AKT1 | P31749 | W80A | 231 | KRAS | P01116 | Y64A |
| 53 | AKT1 | P31749 | W80R | 232 | KRAS | P01116 | Q61P |
| 54 | AKT1 | P31749 | C77F | 233 | KRAS | P01116 | Q61R |
| 55 | AKT1 | P31749 | G311D | 234 | KRAS | P01116 | V14I |
| 56 | AR | P10275 | Q876K | 235 | KRAS | P01116 | F28L |
| 57 | AR | P10275 | F877L | 236 | KRAS | P01116 | E31K |
| 58 | AR | P10275 | H875Y | 237 | KRAS | P01116 | K147N |
| 59 | AR | P10275 | L702H | 238 | KRAS | P01116 | K147E |
| 60 | AR | P10275 | W742C | 239 | KRAS | P01116 | P34L |
| 61 | AR | P10275 | W742L | 240 | KRAS | P01116 | P34R |
| 62 | AR | P10275 | T878S | 241 | KRAS | P01116 | G12R |
| 63 | AR | P10275 | T878A | 242 | KRAS | P01116 | G12S |
| 64 | AR | P10275 | R711I | 243 | KRAS | P01116 | G12V |
| 65 | BRAF | P15056 | K601E | 244 | KRAS | P01116 | G12C |
| 66 | BRAF | P15056 | R462E | 245 | KRAS | P01116 | G12A |
| 67 | BRAF | P15056 | R462I | 246 | KRAS | P01116 | G12F |
| 68 | BRAF | P15056 | L537S | 247 | KRAS | P01116 | G12D |
| 69 | BRAF | P15056 | L584F | 248 | KRAS | P01116 | Q22K |
| 70 | BRAF | P15056 | V600R | 249 | KRAS | P01116 | G60V |
| 71 | BRAF | P15056 | V600M | 250 | KRAS | P01116 | G60R |
| 72 | BRAF | P15056 | V600K | 251 | KRAS | P01116 | G60D |
| 73 | BRAF | P15056 | V600E | 252 | KRAS | P01116 | K117N |
| 74 | BRAF | P15056 | V600D | 253 | KRAS | P01116 | Y32S |
| 75 | BRAF | P15056 | V600A | 254 | KRAS | P01116 | E63K |
| 76 | BRAF | P15056 | D594H | 255 | KRAS | P01116 | R73M |
| 77 | BRAF | P15056 | D594N | 256 | KRAS | P01116 | G13V |
| 78 | BRAF | P15056 | D594A | 257 | KRAS | P01116 | G13S |
| 79 | BRAF | P15056 | D594E | 258 | KRAS | P01116 | G13R |
| 80 | BRAF | P15056 | D594G | 259 | KRAS | P01116 | G13D |
| 81 | BRAF | P15056 | D594Y | 260 | KRAS | P01116 | G13A |
| 82 | BRAF | P15056 | D594V | 261 | KRAS | P01116 | Q22R |
| 83 | BRAF | P15056 | L597V | 262 | KRAS | P01116 | Q22E |
| 84 | BRAF | P15056 | Y472C | 263 | KRAS | P01116 | A59T |
| 85 | BRAF | P15056 | L597R | 264 | KRAS | P01116 | I36L |
| 86 | BRAF | P15056 | L597S | 265 | KRAS | P01116 | A11V |
| 87 | BRAF | P15056 | L597Q | 266 | KRAS | P01116 | S65N |
| 88 | BRAF | P15056 | K483E | 267 | KRAS | P01116 | G13C |
| 89 | BRAF | P15056 | R462K | 268 | KRAS | P01116 | L19F |
| 90 | BRAF | P15056 | I592V | 269 | KRAS | P01116 | E62K |
| 91 | BRAF | P15056 | K601N | 270 | KRAS | P01116 | A59G |
| 92 | BRAF | P15056 | K601R | 271 | KRAS | P01116 | A59E |
| 93 | BRAF | P15056 | K601Q | 272 | KRAS | P01116 | Q61K |
| 94 | BRAF | P15056 | K601T | 273 | KRAS | P01116 | Y71H |
| 95 | BRAF | P15056 | T529N | 274 | KRAS | P01116 | S17N |
| 96 | BRAF | P15056 | T529M | 275 | KRAS | P01116 | A146T |
| 97 | BRAF | P15056 | T529I | 276 | KRAS | P01116 | I36M |
| 98 | BRAF | P15056 | G466R | 277 | KRAS | P01116 | A146V |
| 99 | BRAF | P15056 | G466V | 278 | KRAS | P01116 | A146P |
| 100 | BRAF | P15056 | G466A | 279 | KRAS | P01116 | R68S |
| 101 | BRAF | P15056 | G596S | 280 | KRAS | P01116 | D119N |
| 102 | BRAF | P15056 | G466E | 281 | KRAS | P01116 | D119A |
| 103 | BRAF | P15056 | G596R | 282 | KRAS | P01116 | D33E |
| 104 | BRAF | P15056 | W531C | 283 | KRAS | P01116 | A18D |
| 105 | BRAF | P15056 | K483M | 284 | KRAS | P01116 | C118S |
| 106 | BRAF | P15056 | G596C | 285 | MAP2K1 | Q02750 | C121S |
| 107 | BRAF | P15056 | G596D | 286 | MAP2K1 | Q02750 | K97M |
| 108 | BRAF | P15056 | G596V | 287 | MAP2K1 | Q02750 | Y130C |
| 109 | BRAF | P15056 | I592M | 288 | MAP2K1 | Q02750 | E144K |
| 110 | BRAF | P15056 | N581D | 289 | MAP2K1 | Q02750 | L115R |
| 111 | BRAF | P15056 | N581H | 290 | MAP2K1 | Q02750 | L115P |
| 112 | BRAF | P15056 | N581I | 291 | MAP2K1 | Q02750 | I111P |
| 113 | BRAF | P15056 | N581S | 292 | MAP2K1 | Q02750 | I111R |
| 114 | BRAF | P15056 | N581T | 293 | MAP2K1 | Q02750 | I111S |
| 115 | BRAF | P15056 | N581Y | 294 | MAP2K1 | Q02750 | I111N |
| 116 | BRAF | P15056 | L505H | 295 | MAP2K1 | Q02750 | I111A |
| 117 | BRAF | P15056 | F595S | 296 | MAP2K1 | Q02750 | L215P |
| 118 | BRAF | P15056 | A598V | 297 | MAP2K1 | Q02750 | I99T |
| 119 | BRAF | P15056 | F595L | 298 | MAP2K1 | Q02750 | V211D |
| 120 | BRAF | P15056 | G464R | 299 | MAP2K1 | Q02750 | S218D |
| 121 | BRAF | P15056 | I463S | 300 | MAP2K1 | Q02750 | F129L |
| 122 | BRAF | P15056 | H539P | 301 | MAP2K1 | Q02750 | V154I |
| 123 | BRAF | P15056 | A598T | 302 | MAP2K1 | Q02750 | G128D |
| 124 | BRAF | P15056 | G464V | 303 | MAP2K1 | Q02750 | P193S |
| 125 | BRAF | P15056 | G464E | 304 | MAP2K1 | Q02750 | G128V |
| 126 | BRAF | P15056 | K499E | 305 | MAP2K2 | P36507 | S154F |
| 127 | BRAF | P15056 | T599R | 306 | MAP2K2 | P36507 | P197S |
| 128 | BRAF | P15056 | F468C | 307 | MAP2K2 | P36507 | K101M |
| 129 | BRAF | P15056 | R95T | 308 | MAP2K2 | P36507 | C125S |
| 130 | BRAF | P15056 | F468S | 309 | MAP2K2 | P36507 | G83S |
| 131 | BRAF | P15056 | T599I | 310 | MAP2K4 | P45985 | S251N |
| 132 | BRAF | P15056 | V471F | 311 | MAP2K4 | P45985 | S251I |
| 133 | BRAF | P15056 | G469S | 312 | MAP2K4 | P45985 | L146F |
| 134 | BRAF | P15056 | G469R | 313 | MAP2K4 | P45985 | S233A |
| 135 | BRAF | P15056 | G469V | 314 | MAP2K4 | P45985 | S184L |
| 136 | BRAF | P15056 | G469A | 315 | MAP2K4 | P45985 | R228K |
| 137 | BRAF | P15056 | G469E | 316 | MAP2K4 | P45985 | N234I |
| 138 | BRAF | P15056 | V600G | 317 | MAP2K4 | P45985 | D186G |
| 139 | BRAF | P15056 | G469L | 318 | MAP2K7 | O14733 | E116K |
| 140 | BRAF | P15056 | E501K | 319 | MAT2A | P31153 | P151S |
| 141 | BRAF | P15056 | E501G | 320 | MSH2 | P43246 | G674R |
| 142 | BRAF | P15056 | V600L | 321 | MSH2 | P43246 | D543N |
| 143 | BRAF | P15056 | H574Q | 322 | MSH2 | P43246 | G322D |
| 144 | CASP1 | P29466 | C331W | 323 | MSH6 | P52701 | V509A |
| 145 | CASP1 | P29466 | A329T | 324 | MSH6 | P52701 | I972F |
| 146 | CASP1 | P29466 | S236F | 325 | MTOR | P42345 | W2101L |
| 147 | CASP8 | Q14790 | L336F | 326 | MTOR | P42345 | E2033V |
| 148 | CSNK2A1 | P68400 | V162A | 327 | MTOR | P42345 | A2100T |
| 149 | EZH2 | Q15910 | F665V | 328 | MTOR | P42345 | A2034V |
| 150 | EZH2 | Q15910 | N670K | 329 | MTOR | P42345 | F2108L |
| 151 | EZH2 | Q15910 | A677G | 330 | NT5C2 | P49902 | L57F |
| 152 | EZH2 | Q15910 | N668S | 331 | NT5C2 | P49902 | T256I |
| 153 | EZH2 | Q15910 | Y641S | 332 | NT5C2 | P49902 | S443N |
| 154 | EZH2 | Q15910 | Y641N | 333 | NT5C2 | P49902 | K359Q |
| 155 | EZH2 | Q15910 | Y641H | 334 | PPARG | P37231 | I277N |
| 156 | EZH2 | Q15910 | Y641F | 335 | PPARG | P37231 | R316H |
| 157 | EZH2 | Q15910 | Y641C | 336 | PPARG | P37231 | R316C |
| 158 | EZH2 | Q15910 | R685H | 337 | PPARG | P37231 | R385Q |
| 159 | HGF | P14210 | Y544F | 338 | PPARG | P37231 | T356K |
| 160 | HGF | P14210 | R702H | 339 | PPARG | P37231 | Q314P |
| 161 | HGF | P14210 | R630P | 340 | PPARG | P37231 | G372S |
| 162 | HGF | P14210 | W617C | 341 | PTPN11 | Q06124 | A72S |
| 163 | HGF | P14210 | Y619C | 342 | PTPN11 | Q06124 | F71I |
| 164 | HRAS | P01112 | P34R | 343 | PTPN11 | Q06124 | T52S |
| 165 | HRAS | P01112 | A59G | 344 | PTPN11 | Q06124 | H114Y |
| 166 | HRAS | P01112 | G13D | 345 | PTPN11 | Q06124 | G13D |
| 167 | HRAS | P01112 | A146P | 346 | PTPN11 | Q06124 | Q79R |
| 168 | HRAS | P01112 | A146V | 347 | PTPN11 | Q06124 | E76V |
| 169 | HRAS | P01112 | A146T | 348 | PTPN11 | Q06124 | E76K |
| 170 | HRAS | P01112 | D33N | 349 | PTPN11 | Q06124 | E76A |
| 171 | HRAS | P01112 | Q22K | 350 | PTPN11 | Q06124 | E76G |
| 172 | HRAS | P01112 | F28L | 351 | PTPN11 | Q06124 | F71L |
| 173 | HRAS | P01112 | G13I | 352 | PTPN11 | Q06124 | A72D |
| 174 | HRAS | P01112 | G13C | 353 | PTPN11 | Q06124 | A72V |
| 175 | HRAS | P01112 | G13A | 354 | PTPN11 | Q06124 | A72T |
| 176 | HRAS | P01112 | G13V | 355 | PTPN11 | Q06124 | E69K |
| 177 | HRAS | P01112 | G13R | 356 | PTPN11 | Q06124 | I282V |
| 178 | HRAS | P01112 | G13S | 357 | WNK1 | Q9H4A3 | D349N |
| 179 | HRAS | P01112 | A59T |  |  |  |  |

**Table S7.** Passenger mutations in the training dataset.

| Index | Gene name | Uniprot ID | Mutation | Index | Gene name | Uniprot ID | Mutation |
| --- | --- | --- | --- | --- | --- | --- | --- |
| 1 | ABL1 | P00519 | I313T | 1464 | ITGAL | P20701 | M179V |
| 2 | ABL1 | P00519 | I313V | 1465 | ITGAL | P20701 | F193L |
| 3 | ABL1 | P00519 | K378R | 1466 | ITGAL | P20701 | Y282H |
| 4 | ABL1 | P00519 | V270M | 1467 | ITGAL | P20701 | G271S |
| 5 | ABL1 | P00519 | W430C | 1468 | ITGAL | P20701 | G287E |
| 6 | ABL1 | P00519 | W430L | 1469 | ITGAL | P20701 | R281C |
| 7 | ABL1 | P00519 | Y257C | 1470 | ITGAL | P20701 | R281L |
| 8 | ABL1 | P00519 | C369Y | 1471 | ITGAL | P20701 | R281H |
| 9 | ABL1 | P00519 | E466D | 1472 | ITGAL | P20701 | D181N |
| 10 | ABL1 | P00519 | V338F | 1473 | ITGAL | P20701 | K257R |
| 11 | ABL1 | P00519 | E431G | 1474 | ITGAL | P20701 | I279N |
| 12 | ABL1 | P00519 | F493L | 1475 | ITGAL | P20701 | Q296K |
| 13 | ABL1 | P00519 | Y312H | 1476 | ITGAL | P20701 | T231A |
| 14 | ABL1 | P00519 | Y312C | 1477 | ITGAL | P20701 | E171K |
| 15 | ABL1 | P00519 | R367Q | 1478 | ITGAL | P20701 | S166N |
| 16 | ABL1 | P00519 | L301F | 1479 | ITGAL | P20701 | E294K |
| 17 | ABL1 | P00519 | V268L | 1480 | ITGAL | P20701 | E294D |
| 18 | ABL1 | P00519 | I360S | 1481 | ITK | Q08881 | S499F |
| 19 | ABL1 | P00519 | I360V | 1482 | ITK | Q08881 | V409L |
| 20 | ABL1 | P00519 | N368S | 1483 | ITK | Q08881 | F501L |
| 21 | ABL1 | P00519 | N368D | 1484 | ITK | Q08881 | G423V |
| 22 | ABL1 | P00519 | V339A | 1485 | ITK | Q08881 | M411V |
| 23 | ABL1 | P00519 | V339L | 1486 | ITK | Q08881 | M438T |
| 24 | ABL1 | P00519 | F497V | 1487 | ITK | Q08881 | Q420K |
| 25 | ABL1 | P00519 | V268A | 1488 | ITK | Q08881 | D500V |
| 26 | ABL1 | P00519 | R460H | 1489 | ITK | Q08881 | R505T |
| 27 | ABL1 | P00519 | V335M | 1490 | ITK | Q08881 | M503V |
| 28 | ABL1 | P00519 | G426V | 1491 | ITK | Q08881 | D445N |
| 29 | ABL1 | P00519 | T267A | 1492 | ITK | Q08881 | G375V |
| 30 | ABL1 | P00519 | T267M | 1493 | ITK | Q08881 | L443R |
| 31 | ABL1 | P00519 | A337T | 1494 | ITK | Q08881 | M410I |
| 32 | ABL1 | P00519 | M318I | 1495 | ITK | Q08881 | G372V |
| 33 | ABL1 | P00519 | M318T | 1496 | ITK | Q08881 | S414Y |
| 34 | ABL1 | P00519 | M496I | 1497 | ITK | Q08881 | V377M |
| 35 | ABL1 | P00519 | G254R | 1498 | ITK | Q08881 | R486I |
| 36 | ABL1 | P00519 | T319I | 1499 | ITK | Q08881 | V357M |
| 37 | ABL1 | P00519 | R362K | 1500 | ITK | Q08881 | K497N |
| 38 | ABL1 | P00519 | Q300H | 1501 | ITK | Q08881 | F374C |
| 39 | ABL1 | P00519 | R332W | 1502 | ITK | Q08881 | V490L |
| 40 | ABL1 | P00519 | G321R | 1503 | ITK | Q08881 | E368Q |
| 41 | ABL1 | P00519 | G321E | 1504 | ITK | Q08881 | L473M |
| 42 | ABL1 | P00519 | V256L | 1505 | ITK | Q08881 | L433R |
| 43 | ADCY10 | Q96PN6 | K334N | 1506 | ITK | Q08881 | E436A |
| 44 | ADCY10 | Q96PN6 | F96I | 1507 | JAK2 | O60674 | G552S |
| 45 | ADCY10 | Q96PN6 | A100V | 1508 | JAK2 | O60674 | G861W |
| 46 | ADCY10 | Q96PN6 | V172M | 1509 | JAK2 | O60674 | F631C |
| 47 | ADCY10 | Q96PN6 | D99E | 1510 | JAK2 | O60674 | A676V |
| 48 | ADCY10 | Q96PN6 | Y404C | 1511 | JAK2 | O60674 | I559L |
| 49 | ADCY10 | Q96PN6 | F96C | 1512 | JAK2 | O60674 | E898K |
| 50 | ADCY10 | Q96PN6 | D47H | 1513 | JAK2 | O60674 | M929L |
| 51 | ADCY10 | Q96PN6 | V175M | 1514 | JAK2 | O60674 | K912T |
| 52 | ADCY10 | Q96PN6 | F336L | 1515 | JAK2 | O60674 | R980T |
| 53 | ADCY10 | Q96PN6 | S49A | 1516 | JAK2 | O60674 | S550G |
| 54 | ADCY10 | Q96PN6 | G408D | 1517 | JAK2 | O60674 | G856S |
| 55 | ADCY10 | Q96PN6 | G408C | 1518 | JAK2 | O60674 | D976N |
| 56 | ADCY10 | Q96PN6 | A97V | 1519 | JAK2 | O60674 | Q553L |
| 57 | ADCY10 | Q96PN6 | M54I | 1520 | JAK2 | O60674 | I682F |
| 58 | ADCY10 | Q96PN6 | K95N | 1521 | JAK2 | O60674 | V863M |
| 59 | ADCY10 | Q96PN6 | M419K | 1522 | JAK2 | O60674 | A676D |
| 60 | ADCY10 | Q96PN6 | R142Q | 1523 | JAK2 | O60674 | A880T |
| 61 | ADCY10 | Q96PN6 | K144N | 1524 | JAK2 | O60674 | L697R |
| 62 | ADCY10 | Q96PN6 | R176C | 1525 | JAK2 | O60674 | T636I |
| 63 | ADCY10 | Q96PN6 | Q179H | 1526 | JAK2 | O60674 | P933R |
| 64 | ADORA2A | P29274 | K227M | 1527 | JAK2 | O60674 | P933Q |
| 65 | ADORA2A | P29274 | R293P | 1528 | JAK2 | O60674 | V878A |
| 66 | ADORA2A | P29274 | A105V | 1529 | JAK2 | O60674 | V911A |
| 67 | ADORA2A | P29274 | R291P | 1530 | JAK2 | O60674 | C675F |
| 68 | ADORA2A | P29274 | P215S | 1531 | JAK2 | O60674 | F628L |
| 69 | ADORA2A | P29274 | R296H | 1532 | JAK2 | O60674 | F560S |
| 70 | ADORA2A | P29274 | R296L | 1533 | KDR | P35968 | F921C |
| 71 | ADORA2A | P29274 | S35I | 1534 | KDR | P35968 | T376S |
| 72 | ADORA2A | P29274 | P217L | 1535 | KDR | P35968 | G1015D |
| 73 | ADORA2A | P29274 | Y112H | 1536 | KDR | P35968 | E372D |
| 74 | ADORA2A | P29274 | G114C | 1537 | KDR | P35968 | N897I |
| 75 | ADORA2A | P29274 | V275A | 1538 | KDR | P35968 | I362K |
| 76 | ADORA2A | P29274 | V84I | 1539 | KDR | P35968 | G333V |
| 77 | ADORA2A | P29274 | R293C | 1540 | KDR | P35968 | T384M |
| 78 | ADORA2A | P29274 | R293H | 1541 | KDR | P35968 | K353N |
| 79 | ADORA2A | P29274 | R222W | 1542 | KDR | P35968 | V898M |
| 80 | ADORA2A | P29274 | R222Q | 1543 | KDR | P35968 | V898A |
| 81 | ADORA2A | P29274 | A30V | 1544 | KDR | P35968 | M386T |
| 82 | ADORA2A | P29274 | A63D | 1545 | KDR | P35968 | V899I |
| 83 | ADORA2A | P29274 | R300H | 1546 | KDR | P35968 | L1049W |
| 84 | ADORA2A | P29274 | I92M | 1547 | KDR | P35968 | G380W |
| 85 | ADORA2A | P29274 | H278N | 1548 | KDR | P35968 | L889F |
| 86 | ADORA2A | P29274 | M211T | 1549 | KDR | P35968 | R349T |
| 87 | ADORA2A | P29274 | K301N | 1550 | KDR | P35968 | R347C |
| 88 | ADORA2A | P29274 | R300C | 1551 | KDR | P35968 | R347S |
| 89 | ADORA2A | P29274 | T41I | 1552 | KDR | P35968 | R347P |
| 90 | ADORA2A | P29274 | A231V | 1553 | KDR | P35968 | A866S |
| 91 | ADORA2A | P29274 | M270I | 1554 | KDR | P35968 | M1016K |
| 92 | ADORA2A | P29274 | R220W | 1555 | KDR | P35968 | M1016I |
| 93 | ADORA2A | P29274 | R111W | 1556 | KDR | P35968 | G893D |
| 94 | ADORA2A | P29274 | R111L | 1557 | KDR | P35968 | L1035I |
| 95 | ADORA2A | P29274 | Y43H | 1558 | KDR | P35968 | L1035F |
| 96 | ADORA2A | P29274 | L276F | 1559 | KDR | P35968 | P359H |
| 97 | ADRB2 | P07550 | R63H | 1560 | KDR | P35968 | L882V |
| 98 | ADRB2 | P07550 | Y316C | 1561 | KDR | P35968 | T376A |
| 99 | ADRB2 | P07550 | T118M | 1562 | KDR | P35968 | S373F |
| 100 | ADRB2 | P07550 | A200V | 1563 | KDR | P35968 | A379V |
| 101 | ADRB2 | P07550 | M82T | 1564 | KDR | P35968 | R347H |
| 102 | ADRB2 | P07550 | A119T | 1565 | KDR | P35968 | K378N |
| 103 | ADRB2 | P07550 | S111F | 1566 | KDR | P35968 | C1045F |
| 104 | ADRB2 | P07550 | Y174N | 1567 | KDR | P35968 | N900S |
| 105 | ADRB2 | P07550 | V117I | 1568 | KDR | P35968 | T343M |
| 106 | ADRB2 | P07550 | V297M | 1569 | KDR | P35968 | T343K |
| 107 | ADRB2 | P07550 | A271S | 1570 | KDR | P35968 | A331V |
| 108 | ADRB2 | P07550 | F289S | 1571 | KDR | P35968 | A352S |
| 109 | ADRB2 | P07550 | D331V | 1572 | KDR | P35968 | A352V |
| 110 | ADRB2 | P07550 | I112M | 1573 | KDR | P35968 | L400F |
| 111 | ADRB2 | P07550 | L311R | 1574 | KDR | P35968 | G1048V |
| 112 | ADRB2 | P07550 | K267T | 1575 | KDR | P35968 | L355R |
| 113 | ADRB2 | P07550 | N322S | 1576 | KDR | P35968 | I1044T |
| 114 | ADRB2 | P07550 | H296R | 1577 | KDR | P35968 | L886F |
| 115 | ADRB2 | P07550 | C327F | 1578 | KIF11 | P52732 | L214F |
| 116 | ADRB2 | P07550 | G276R | 1579 | KIF11 | P52732 | V298I |
| 117 | ADRB2 | P07550 | G276D | 1580 | KIF11 | P52732 | I332M |
| 118 | ADRB2 | P07550 | T66M | 1581 | KIF11 | P52732 | Q212H |
| 119 | ADRB2 | P07550 | Q197K | 1582 | KIF11 | P52732 | L172V |
| 120 | ADRB2 | P07550 | V206M | 1583 | KIF11 | P52732 | N271S |
| 121 | AKT1 | P31749 | T81N | 1584 | KIF11 | P52732 | G73E |
| 122 | AKT1 | P31749 | I290M | 1585 | KIF11 | P52732 | R138H |
| 123 | AKT1 | P31749 | Y229C | 1586 | KIF11 | P52732 | E116G |
| 124 | AKT1 | P31749 | G232R | 1587 | KIF11 | P52732 | A103S |
| 125 | AKT1 | P31749 | N199I | 1588 | KIF11 | P52732 | A103V |
| 126 | AKT1 | P31749 | L181F | 1589 | KIF11 | P52732 | R297M |
| 127 | AKT1 | P31749 | T211I | 1590 | KIF11 | P52732 | A230V |
| 128 | AKT1 | P31749 | K14N | 1591 | KIF11 | P52732 | G108A |
| 129 | AKT1 | P31749 | K14I | 1592 | KIF11 | P52732 | A218T |
| 130 | AKT1 | P31749 | N204K | 1593 | KIF11 | P52732 | I336V |
| 131 | AKT1 | P31749 | K163R | 1594 | KIF11 | P52732 | Y104C |
| 132 | AKT1 | P31749 | E85K | 1595 | KIF11 | P52732 | K357N |
| 133 | AKT1 | P31749 | G232W | 1596 | KRAS | P01116 | G10E |
| 134 | AKT1 | P31749 | G157S | 1597 | KRAS | P01116 | G10V |
| 135 | AKT1 | P31749 | V201I | 1598 | KRAS | P01116 | G10R |
| 136 | AKT1 | P31749 | D292E | 1599 | KRAS | P01116 | E91G |
| 137 | AKT1 | P31749 | V320L | 1600 | KRAS | P01116 | E91K |
| 138 | AKT1 | P31749 | T291A | 1601 | KRAS | P01116 | R97I |
| 139 | AKT1 | P31749 | T291I | 1602 | KRAS | P01116 | P121H |
| 140 | AKT1 | P31749 | Y272C | 1603 | KRAS | P01116 | S145L |
| 141 | AKT1 | P31749 | F55Y | 1604 | KRAS | P01116 | K16R |
| 142 | AKT1 | P31749 | F55L | 1605 | KRAS | P01116 | Q99E |
| 143 | AKT1 | P31749 | G159D | 1606 | KRAS | P01116 | G15S |
| 144 | AKT1 | P31749 | D274H | 1607 | KRAS | P01116 | V9I |
| 145 | AKT1 | P31749 | A177S | 1608 | KRAS | P01116 | G15D |
| 146 | AKT1 | P31749 | G162S | 1609 | KRAS | P01116 | V9D |
| 147 | AKT1 | P31749 | R273Q | 1610 | KRAS | P01116 | I21R |
| 148 | ALB | P02768 | D211N | 1611 | KRAS | P01116 | D92Y |
| 149 | ALB | P02768 | A234D | 1612 | KRAS | P01116 | R149G |
| 150 | ALB | P02768 | R138Q | 1613 | KRAS | P01116 | V7G |
| 151 | ALB | P02768 | F575C | 1614 | KRAS | P01116 | V7M |
| 152 | ALB | P02768 | D283E | 1615 | KRAS | P01116 | T144P |
| 153 | ALB | P02768 | D283N | 1616 | KRAS | P01116 | D69G |
| 154 | ALB | P02768 | R210G | 1617 | KRAS | P01116 | M72I |
| 155 | ALB | P02768 | R210W | 1618 | KRAS | P01116 | T35I |
| 156 | ALB | P02768 | R210Q | 1619 | KRAS | P01116 | T35A |
| 157 | ALB | P02768 | I288N | 1620 | KRAS | P01116 | D30Y |
| 158 | ALB | P02768 | I288T | 1621 | KRAS | P01116 | C80S |
| 159 | ALB | P02768 | A473V | 1622 | KRAS | P01116 | R149K |
| 160 | ALB | P02768 | C462F | 1623 | KRAS | P01116 | D30E |
| 161 | ALB | P02768 | A315T | 1624 | KRAS | P01116 | V8I |
| 162 | ALB | P02768 | S504F | 1625 | KRAS | P01116 | D57N |
| 163 | ALB | P02768 | Y174F | 1626 | KRAS | P01116 | P121S |
| 164 | ALB | P02768 | R34W | 1627 | KRAS | P01116 | D57Y |
| 165 | ALB | P02768 | N507K | 1628 | KRAS | P01116 | Q70P |
| 166 | ALB | P02768 | E424K | 1629 | KRAS | P01116 | M67L |
| 167 | ALB | P02768 | L206F | 1630 | KRAS | P01116 | I93F |
| 168 | ALB | P02768 | Y164H | 1631 | KRAS | P01116 | G115E |
| 169 | ALB | P02768 | A602S | 1632 | KRAS | P01116 | V7E |
| 170 | ALB | P02768 | A601V | 1633 | KRAS | P01116 | H95L |
| 171 | ALB | P02768 | G458D | 1634 | KRAS | P01116 | E37K |
| 172 | ALB | P02768 | M572I | 1635 | LIMK2 | P53671 | L458V |
| 173 | ALB | P02768 | L355V | 1636 | LIMK2 | P53671 | F341V |
| 174 | ALB | P02768 | R469K | 1637 | LIMK2 | P53671 | N456T |
| 175 | ALB | P02768 | D348N | 1638 | LIMK2 | P53671 | I392F |
| 176 | ALB | P02768 | A237T | 1639 | LIMK2 | P53671 | R474Q |
| 177 | ALB | P02768 | T379I | 1640 | LIMK2 | P53671 | R450W |
| 178 | ALB | P02768 | R434P | 1641 | LIMK2 | P53671 | L472R |
| 179 | ALB | P02768 | R434H | 1642 | LIMK2 | P53671 | E361Q |
| 180 | ALB | P02768 | R434C | 1643 | LIMK2 | P53671 | L401M |
| 181 | ALB | P02768 | Y162N | 1644 | LIMK2 | P53671 | C365R |
| 182 | ALB | P02768 | R246I | 1645 | LIMK2 | P53671 | R381C |
| 183 | ALB | P02768 | C277W | 1646 | LTA4H | P09960 | V28G |
| 184 | ALB | P02768 | E555Q | 1647 | LTA4H | P09960 | V293L |
| 185 | ALB | P02768 | F94S | 1648 | LTA4H | P09960 | P180L |
| 186 | ALB | P02768 | H170Y | 1649 | LTA4H | P09960 | E160Q |
| 187 | ALB | P02768 | S226G | 1650 | LTA4H | P09960 | S106Y |
| 188 | ALB | P02768 | Y185F | 1651 | LTA4H | P09960 | S106F |
| 189 | ALB | P02768 | L599R | 1652 | LTA4H | P09960 | F107S |
| 190 | ALB | P02768 | Y185C | 1653 | LTA4H | P09960 | K566R |
| 191 | ALB | P02768 | E407D | 1654 | LTA4H | P09960 | R327H |
| 192 | ALB | P02768 | L299V | 1655 | LTA4H | P09960 | R327C |
| 193 | ALB | P02768 | H266R | 1656 | LTA4H | P09960 | V161L |
| 194 | ALB | P02768 | A374S | 1657 | LTA4H | P09960 | F107L |
| 195 | ALB | P02768 | N42D | 1658 | LTA4H | P09960 | E108K |
| 196 | ALB | P02768 | V240A | 1659 | LTA4H | P09960 | Y268C |
| 197 | ALB | P02768 | D279Y | 1660 | MALT1 | Q9UDY8 | M351I |
| 198 | ALB | P02768 | L454Q | 1661 | MALT1 | Q9UDY8 | R576W |
| 199 | ALB | P02768 | D279N | 1662 | MALT1 | Q9UDY8 | R392H |
| 200 | ALB | P02768 | E165D | 1663 | MALT1 | Q9UDY8 | R392C |
| 201 | ALB | P02768 | D207N | 1664 | MALT1 | Q9UDY8 | K543R |
| 202 | ALB | P02768 | G352D | 1665 | MALT1 | Q9UDY8 | H356Y |
| 203 | ALB | P02768 | V405A | 1666 | MALT1 | Q9UDY8 | K360R |
| 204 | ALB | P02768 | G455E | 1667 | MALT1 | Q9UDY8 | D716H |
| 205 | ALB | P02768 | P142L | 1668 | MALT1 | Q9UDY8 | E497V |
| 206 | ALB | P02768 | S311F | 1669 | MALT1 | Q9UDY8 | E500D |
| 207 | ALB | P02768 | S478F | 1670 | MALT1 | Q9UDY8 | G349R |
| 208 | ALB | P02768 | G231E | 1671 | MALT1 | Q9UDY8 | I712V |
| 209 | ALB | P02768 | L370R | 1672 | MALT1 | Q9UDY8 | T492M |
| 210 | ALB | P02768 | V47M | 1673 | MALT1 | Q9UDY8 | S382L |
| 211 | ALB | P02768 | N42K | 1674 | MALT1 | Q9UDY8 | I501T |
| 212 | ALB | P02768 | T551I | 1675 | MAOB | P27338 | T202A |
| 213 | ALB | P02768 | S459N | 1676 | MAOB | P27338 | G57R |
| 214 | ALB | P02768 | P471S | 1677 | MAOB | P27338 | N170K |
| 215 | ALB | P02768 | F43L | 1678 | MAOB | P27338 | Q206H |
| 216 | ALB | P02768 | V506A | 1679 | MAOB | P27338 | F168L |
| 217 | ALB | P02768 | A175S | 1680 | MAOB | P27338 | S200L |
| 218 | ALB | P02768 | D37H | 1681 | MAOB | P27338 | T399S |
| 219 | ALB | P02768 | D37N | 1682 | MAOB | P27338 | H90Y |
| 220 | ALB | P02768 | R242C | 1683 | MAOB | P27338 | I316V |
| 221 | ALB | P02768 | D574N | 1684 | MAOB | P27338 | T174S |
| 222 | ALB | P02768 | R372S | 1685 | MAOB | P27338 | G58R |
| 223 | ALB | P02768 | E417K | 1686 | MAOB | P27338 | G205A |
| 224 | ALB | P02768 | G596C | 1687 | MAOB | P27338 | V61L |
| 225 | ALB | P02768 | A241G | 1688 | MAOB | P27338 | V61F |
| 226 | ALB | P02768 | A241V | 1689 | MAP2K1 | Q02750 | M94I |
| 227 | ALB | P02768 | M147V | 1690 | MAP2K1 | Q02750 | R189K |
| 228 | ALB | P02768 | L262I | 1691 | MAP2K1 | Q02750 | V127M |
| 229 | ALB | P02768 | L404V | 1692 | MAP2K1 | Q02750 | R96K |
| 230 | ALB | P02768 | V367A | 1693 | MAP2K1 | Q02750 | V224M |
| 231 | ALB | P02768 | A239V | 1694 | MAP2K1 | Q02750 | Y229H |
| 232 | ALB | P02768 | S459T | 1695 | MAP2K1 | Q02750 | S212N |
| 233 | ALB | P02768 | A278V | 1696 | MAP2K1 | Q02750 | H188Y |
| 234 | ALB | P02768 | E378K | 1697 | MAP2K1 | Q02750 | A132V |
| 235 | ALB | P02768 | E208K | 1698 | MAP2K1 | Q02750 | G79V |
| 236 | ALB | P02768 | P176L | 1699 | MAP2K1 | Q02750 | G79S |
| 237 | ALB | P02768 | A430V | 1700 | MAP2K1 | Q02750 | A76S |
| 238 | ALB | P02768 | E316K | 1701 | MAP2K1 | Q02750 | A76V |
| 239 | ALB | P02768 | Y174H | 1702 | MAP2K1 | Q02750 | G80S |
| 240 | ALB | P02768 | E309K | 1703 | MAP2K1 | Q02750 | S194A |
| 241 | ALB | P02768 | S216L | 1704 | MAP2K1 | Q02750 | G213V |
| 242 | ALB | P02768 | L179F | 1705 | MAP2K1 | Q02750 | M146I |
| 243 | ALB | P02768 | S513L | 1706 | MAP2K1 | Q02750 | R227K |
| 244 | ALB | P02768 | K543E | 1707 | MAP2K1 | Q02750 | K192N |
| 245 | ALB | P02768 | V317L | 1708 | MAP2K1 | Q02750 | L118V |
| 246 | ALB | P02768 | D261Y | 1709 | MAP2K1 | Q02750 | L206F |
| 247 | ALB | P02768 | P176A | 1710 | MAP2K1 | Q02750 | S150F |
| 248 | ALB | P02768 | K548N | 1711 | MAP2K1 | Q02750 | N78S |
| 249 | ALB | P02768 | T379S | 1712 | MAP2K2 | P36507 | V228M |
| 250 | ALB | P02768 | K223Q | 1713 | MAP2K2 | P36507 | G214V |
| 251 | ALB | P02768 | E155K | 1714 | MAP2K2 | P36507 | G214R |
| 252 | ALB | P02768 | E155V | 1715 | MAP2K2 | P36507 | Y134C |
| 253 | AMD1 | P17707 | E11K | 1716 | MAP2K2 | P36507 | E77G |
| 254 | AMD1 | P17707 | D317N | 1717 | MAP2K2 | P36507 | E77K |
| 255 | AMD1 | P17707 | L65P | 1718 | MAP2K2 | P36507 | D151E |
| 256 | AMD1 | P17707 | V16G | 1719 | MAP2K2 | P36507 | Q157H |
| 257 | AMD1 | P17707 | S251F | 1720 | MAP2K2 | P36507 | S198C |
| 258 | AMD1 | P17707 | D174N | 1721 | MAP2K2 | P36507 | V215E |
| 259 | AMD1 | P17707 | S229L | 1722 | MAP2K2 | P36507 | I200T |
| 260 | AMD1 | P17707 | N224S | 1723 | MAP2K2 | P36507 | A80V |
| 261 | AMD1 | P17707 | N224D | 1724 | MAP2K2 | P36507 | G217C |
| 262 | AMD1 | P17707 | L14M | 1725 | MAP2K2 | P36507 | V85G |
| 263 | AMD1 | P17707 | N319S | 1726 | MAP2K2 | P36507 | R231C |
| 264 | AMD1 | P17707 | I244T | 1727 | MAP2K2 | P36507 | R231H |
| 265 | AMD1 | P17707 | C49S | 1728 | MAP2K2 | P36507 | F133L |
| 266 | AMD1 | P17707 | F192L | 1729 | MAP2K2 | P36507 | C211R |
| 267 | AMD1 | P17707 | E178D | 1730 | MAP2K2 | P36507 | M150L |
| 268 | AMD1 | P17707 | R114C | 1731 | MAP2K2 | P36507 | T230M |
| 269 | AMD1 | P17707 | L13M | 1732 | MAP2K2 | P36507 | R238W |
| 270 | AMD1 | P17707 | T161N | 1733 | MAP2K4 | P45985 | L180V |
| 271 | AMD1 | P17707 | M70R | 1734 | MAP2K4 | P45985 | P123Q |
| 272 | AMD1 | P17707 | Y193C | 1735 | MAP2K4 | P45985 | G166C |
| 273 | AMD1 | P17707 | W157R | 1736 | MAP2K4 | P45985 | F248V |
| 274 | AMD1 | P17707 | Y318H | 1737 | MAP2K4 | P45985 | R170K |
| 275 | AMD1 | P17707 | N258I | 1738 | MAP2K4 | P45985 | K231I |
| 276 | AMD1 | P17707 | E247D | 1739 | MAP2K4 | P45985 | L236F |
| 277 | AMD1 | P17707 | T176N | 1740 | MAP2K4 | P45985 | D101Y |
| 278 | AMD1 | P17707 | E67Q | 1741 | MAP2K4 | P45985 | D101E |
| 279 | AMD1 | P17707 | F7L | 1742 | MAP2K4 | P45985 | G252R |
| 280 | AMY2A | P04746 | Q56H | 1743 | MAP2K4 | P45985 | V130F |
| 281 | AMY2A | P04746 | P79Q | 1744 | MAP2K4 | P45985 | H227D |
| 282 | AMY2A | P04746 | L252M | 1745 | MAP2K4 | P45985 | C246R |
| 283 | AMY2A | P04746 | D251V | 1746 | MAP2K4 | P45985 | C246Y |
| 284 | AMY2A | P04746 | W372C | 1747 | MAP2K4 | P45985 | S182P |
| 285 | AMY2A | P04746 | P69T | 1748 | MAP2K4 | P45985 | D229V |
| 286 | AMY2A | P04746 | I66V | 1749 | MAP2K4 | P45985 | D229N |
| 287 | AMY2A | P04746 | L211I | 1750 | MAP2K4 | P45985 | D229G |
| 288 | AMY2A | P04746 | D251N | 1751 | MAP2K4 | P45985 | A99V |
| 289 | AMY2A | P04746 | Q56P | 1752 | MAP2K4 | P45985 | M178R |
| 290 | AMY2A | P04746 | E270K | 1753 | MAP2K4 | P45985 | P232H |
| 291 | AMY2A | P04746 | H216L | 1754 | MAP2K4 | P45985 | K131R |
| 292 | AMY2A | P04746 | E75K | 1755 | MAP2K4 | P45985 | D96V |
| 293 | AMY2A | P04746 | L181I | 1756 | MAP2K4 | P45985 | S124G |
| 294 | AMY2A | P04746 | P69H | 1757 | MAP2K4 | P45985 | R132I |
| 295 | AMY2A | P04746 | H30N | 1758 | MAP2K4 | P45985 | E171D |
| 296 | AMY2A | P04746 | G179S | 1759 | MAP2K4 | P45985 | H121Q |
| 297 | AMY2A | P04746 | G253C | 1760 | MAP2K4 | P45985 | L237Q |
| 298 | AMY2A | P04746 | G253D | 1761 | MAP2K4 | P45985 | A129T |
| 299 | AMY2A | P04746 | M217T | 1762 | MAP2K4 | P45985 | K143T |
| 300 | AMY2A | P04746 | D251Y | 1763 | MAP2K4 | P45985 | E179K |
| 301 | AMY2A | P04746 | P59S | 1764 | MAP2K4 | P45985 | I108M |
| 302 | AMY2A | P04746 | P59Q | 1765 | MAP2K4 | P45985 | G166D |
| 303 | AMY2A | P04746 | V249G | 1766 | MAP2K4 | P45985 | A112S |
| 304 | AMY2A | P04746 | V249I | 1767 | MAP2K4 | P45985 | A112V |
| 305 | AMY2A | P04746 | P72H | 1768 | MAP2K4 | P45985 | Q142L |
| 306 | AMY2A | P04746 | D371N | 1769 | MAP2K4 | P45985 | I230M |
| 307 | AMY2A | P04746 | P79A | 1770 | MAP2K4 | P45985 | D247Y |
| 308 | AMY2A | P04746 | A121T | 1771 | MAP2K4 | P45985 | D247N |
| 309 | AMY2A | P04746 | R176C | 1772 | MAP2K7 | O14733 | E197K |
| 310 | AMY2A | P04746 | R176H | 1773 | MAP2K7 | O14733 | T190M |
| 311 | AMY2A | P04746 | R352Q | 1774 | MAP2K7 | O14733 | M196I |
| 312 | AMY2A | P04746 | R210K | 1775 | MAP2K7 | O14733 | M196T |
| 313 | AMY2A | P04746 | R76I | 1776 | MAP2K7 | O14733 | L250V |
| 314 | AMY2A | P04746 | R76G | 1777 | MAP2K7 | O14733 | L251P |
| 315 | AR | P10275 | Q734H | 1778 | MAP2K7 | O14733 | G132R |
| 316 | AR | P10275 | Y835C | 1779 | MAP2K7 | O14733 | G132C |
| 317 | AR | P10275 | D891N | 1780 | MAP2K7 | O14733 | R209Q |
| 318 | AR | P10275 | D891Y | 1781 | MAP2K7 | O14733 | R209W |
| 319 | AR | P10275 | R841L | 1782 | MAP2K7 | O14733 | R152W |
| 320 | AR | P10275 | R841H | 1783 | MAP2K7 | O14733 | G132S |
| 321 | AR | P10275 | Y764C | 1784 | MAP2K7 | O14733 | G129D |
| 322 | AR | P10275 | L881Q | 1785 | MAP2K7 | O14733 | G129S |
| 323 | AR | P10275 | E707D | 1786 | MAP2K7 | O14733 | V134A |
| 324 | AR | P10275 | P893L | 1787 | MAP2K7 | O14733 | V180M |
| 325 | AR | P10275 | M788I | 1788 | MAP2K7 | O14733 | T103M |
| 326 | AR | P10275 | L831P | 1789 | MAP2K7 | O14733 | D119Y |
| 327 | AR | P10275 | F814V | 1790 | MAP2K7 | O14733 | A147V |
| 328 | AR | P10275 | E830K | 1791 | MAP2K7 | O14733 | E204K |
| 329 | AR | P10275 | Y740H | 1792 | MAP2K7 | O14733 | A203T |
| 330 | AR | P10275 | Y740C | 1793 | MAP2K7 | O14733 | M196V |
| 331 | AR | P10275 | M833R | 1794 | MAP2K7 | O14733 | N248S |
| 332 | AR | P10275 | V716M | 1795 | MAPK1 | P28482 | I84T |
| 333 | AR | P10275 | M896V | 1796 | MAPK1 | P28482 | D318N |
| 334 | AR | P10275 | I836M | 1797 | MAPK1 | P28482 | D321G |
| 335 | AR | P10275 | I836V | 1798 | MAPK1 | P28482 | Q132E |
| 336 | AR | P10275 | G725D | 1799 | MAPK1 | P28482 | D321A |
| 337 | AR | P10275 | P724T | 1800 | MAPK1 | P28482 | D321E |
| 338 | AR | P10275 | E873Q | 1801 | MAPK1 | P28482 | D321N |
| 339 | AR | P10275 | L723F | 1802 | MAPK1 | P28482 | D321V |
| 340 | AR | P10275 | A749D | 1803 | MAPK1 | P28482 | Y131C |
| 341 | AR | P10275 | A749T | 1804 | MAPK1 | P28482 | R135K |
| 342 | AR | P10275 | A749V | 1805 | MAPK1 | P28482 | E33Q |
| 343 | AR | P10275 | L745F | 1806 | MAPK1 | P28482 | D106H |
| 344 | AR | P10275 | F892L | 1807 | MAPK1 | P28482 | I103R |
| 345 | AR | P10275 | D733N | 1808 | MAPK1 | P28482 | D167G |
| 346 | AR | P10275 | D733E | 1809 | MAPK1 | P28482 | I31M |
| 347 | AR | P10275 | D891H | 1810 | MAPK1 | P28482 | L121I |
| 348 | AR | P10275 | L874P | 1811 | MAPK1 | P28482 | C166R |
| 349 | AR | P10275 | E898G | 1812 | MAPK1 | P28482 | A52S |
| 350 | AR | P10275 | E898D | 1813 | MAPK1 | P28482 | E81K |
| 351 | AR | P10275 | A810V | 1814 | MAPK1 | P28482 | Y316C |
| 352 | AR | P10275 | P893S | 1815 | MAPK1 | P28482 | Y316F |
| 353 | AR | P10275 | R753Q | 1816 | MAPK1 | P28482 | L112R |
| 354 | AR | P10275 | E894K | 1817 | MAPK1 | P28482 | D162N |
| 355 | AR | P10275 | M750L | 1818 | MAPK1 | P28482 | D162G |
| 356 | AR | P10275 | A766T | 1819 | MAPK10 | P53779 | H163R |
| 357 | AR | P10275 | V890M | 1820 | MAPK10 | P53779 | K121E |
| 358 | AR | P10275 | G751F | 1821 | MAPK10 | P53779 | D207A |
| 359 | AR | P10275 | G751D | 1822 | MAPK10 | P53779 | D207N |
| 360 | AR | P10275 | G751S | 1823 | MAPK10 | P53779 | V90L |
| 361 | AR | P10275 | G744V | 1824 | MAPK10 | P53779 | M159V |
| 362 | AR | P10275 | A736P | 1825 | MAPK10 | P53779 | Y168H |
| 363 | AR | P10275 | V731M | 1826 | MAPK10 | P53779 | K204E |
| 364 | AR | P10275 | L882I | 1827 | MAPK10 | P53779 | L203F |
| 365 | AR | P10275 | M750T | 1828 | MAPK10 | P53779 | Q75H |
| 366 | AR | P10275 | M750I | 1829 | MAPK10 | P53779 | M159I |
| 367 | AR | P10275 | M750K | 1830 | MAPK10 | P53779 | A366V |
| 368 | AR | P10275 | Q903R | 1831 | MAPK10 | P53779 | E367K |
| 369 | AR | P10275 | Q903H | 1832 | MAPK10 | P53779 | A80V |
| 370 | AR | P10275 | Q903E | 1833 | MAPK10 | P53779 | I124F |
| 371 | AURKA | O14965 | R180K | 1834 | MAPK10 | P53779 | L153V |
| 372 | AURKA | O14965 | R205K | 1835 | MAPK14 | Q16539 | T106I |
| 373 | AURKA | O14965 | K258A | 1836 | MAPK14 | Q16539 | P191H |
| 374 | AURKA | O14965 | S245P | 1837 | MAPK14 | Q16539 | P191S |
| 375 | AURKA | O14965 | E260D | 1838 | MAPK14 | Q16539 | A111T |
| 376 | AURKA | O14965 | W277L | 1839 | MAPK14 | Q16539 | E192Q |
| 377 | AURKA | O14965 | S249L | 1840 | MAPK14 | Q16539 | L113V |
| 378 | AURKA | O14965 | V218F | 1841 | MAPK14 | Q16539 | R49H |
| 379 | AURKA | O14965 | V279L | 1842 | MAPK14 | Q16539 | H148D |
| 380 | AURKA | O14965 | V279A | 1843 | MAPK14 | Q16539 | N82H |
| 381 | AURKA | O14965 | D202G | 1844 | MAPK14 | Q16539 | R149K |
| 382 | AURKA | O14965 | A273E | 1845 | MAPK14 | Q16539 | M194V |
| 383 | AURKA | O14965 | E152K | 1846 | MAPK14 | Q16539 | G36N |
| 384 | AURKA | O14965 | R251K | 1847 | MAPK14 | Q16539 | A51V |
| 385 | AURKA | O14965 | H254Y | 1848 | MAPK14 | Q16539 | V89F |
| 386 | AURKA | O14965 | V206A | 1849 | MAPK14 | Q16539 | V89I |
| 387 | AURKA | O14965 | D132Y | 1850 | MAPK14 | Q16539 | Y35H |
| 388 | AURKA | O14965 | K250N | 1851 | MAPK14 | Q16539 | G110A |
| 389 | AURKA | O14965 | L139V | 1852 | MAPK14 | Q16539 | K249T |
| 390 | AURKA | O14965 | R220G | 1853 | MAPK14 | Q16539 | V158M |
| 391 | AURKA | O14965 | R189Q | 1854 | MAPK7 | Q13164 | L106I |
| 392 | AURKA | O14965 | E175K | 1855 | MAPK7 | Q13164 | A116T |
| 393 | AURKA | O14965 | G145V | 1856 | MAPK7 | Q13164 | R98Q |
| 394 | AURKA | O14965 | D256N | 1857 | MAPK7 | Q13164 | I86T |
| 395 | AURKA | O14965 | V174M | 1858 | MAPK7 | Q13164 | D138N |
| 396 | AURKA | O14965 | G173E | 1859 | MAPK7 | Q13164 | A65V |
| 397 | AURKA | O14965 | G173R | 1860 | MAPK7 | Q13164 | T60I |
| 398 | AURKA | O14965 | Y246H | 1861 | MAPK7 | Q13164 | D143H |
| 399 | AURKA | O14965 | G216R | 1862 | MAPK8 | P45983 | D112Y |
| 400 | AURKA | O14965 | L194F | 1863 | MAPK8 | P45983 | T178M |
| 401 | AURKA | O14965 | S155R | 1864 | MAPK8 | P45983 | M77I |
| 402 | BRAF | P15056 | T470I | 1865 | MAPK8 | P45983 | P184L |
| 403 | BRAF | P15056 | G518D | 1866 | MAPK8 | P45983 | L115I |
| 404 | BRAF | P15056 | L515P | 1867 | MAPK8 | P45983 | N114H |
| 405 | BRAF | P15056 | M564T | 1868 | MAPK8 | P45983 | K166N |
| 406 | BRAF | P15056 | L64I | 1869 | MAPK8 | P45983 | M200I |
| 407 | BRAF | P15056 | L567V | 1870 | MAPK8 | P45983 | G38R |
| 408 | BRAF | P15056 | V480M | 1871 | MAPK8 | P45983 | V260I |
| 409 | BRAF | P15056 | H60R | 1872 | MAPK8 | P45983 | G177K |
| 410 | BRAF | P15056 | A497V | 1873 | MAPK8 | P45983 | G177R |
| 411 | BRAF | P15056 | T57I | 1874 | MAPK8 | P45983 | L115F |
| 412 | BRAF | P15056 | S465F | 1875 | MAPK8 | P45983 | I231V |
| 413 | BRAF | P15056 | V502A | 1876 | MAPK8 | P45983 | E195K |
| 414 | BRAF | P15056 | A91V | 1877 | MAPK8 | P45983 | K166M |
| 415 | BRAF | P15056 | M53T | 1878 | MAPK8 | P45983 | L198F |
| 416 | BRAF | P15056 | D576E | 1879 | MAPK8 | P45983 | A42T |
| 417 | BRAF | P15056 | L577I | 1880 | MAPK8 | P45983 | A42S |
| 418 | BRAF | P15056 | R575K | 1881 | MAT2A | P31153 | G26W |
| 419 | BRAF | P15056 | F583C | 1882 | MAT2A | P31153 | I205T |
| 420 | BRAF | P15056 | G503R | 1883 | MAT2A | P31153 | G69R |
| 421 | BRAF | P15056 | E80D | 1884 | MAT2A | P31153 | G69E |
| 422 | BRAF | P15056 | K591R | 1885 | MAT2A | P31153 | H209L |
| 423 | BRAF | P15056 | K591E | 1886 | MAT2A | P31153 | E57K |
| 424 | BRAF | P15056 | N49I | 1887 | MAT2A | P31153 | K32Q |
| 425 | BRAF | P15056 | M517V | 1888 | MAT2A | P31153 | A55D |
| 426 | BRAF | P15056 | R506G | 1889 | MAT2A | P31153 | L315V |
| 427 | BRAF | P15056 | G593D | 1890 | MAT2A | P31153 | H122R |
| 428 | BRAF | P15056 | G593S | 1891 | MAT2A | P31153 | P255H |
| 429 | BRAF | P15056 | A526V | 1892 | MAT2A | P31153 | S341G |
| 430 | BRAF | P15056 | G534R | 1893 | MAT2A | P31153 | E342K |
| 431 | BRAF | P15056 | F516L | 1894 | MAT2A | P31153 | V54A |
| 432 | BRAF | P15056 | L65M | 1895 | MAT2A | P31153 | Q256H |
| 433 | BRAF | P15056 | L514P | 1896 | MAT2A | P31153 | F139S |
| 434 | BRAF | P15056 | L514I | 1897 | MAT2A | P31153 | I332V |
| 435 | BRAF | P15056 | P75S | 1898 | ME2 | P23368 | R67Q |
| 436 | BRAF | P15056 | I572F | 1899 | ME2 | P23368 | E255K |
| 437 | BRAF | P15056 | P75L | 1900 | ME2 | P23368 | G168D |
| 438 | BRAF | P15056 | H540Q | 1901 | ME2 | P23368 | S447G |
| 439 | BRAF | P15056 | E45K | 1902 | ME2 | P23368 | R165I |
| 440 | BRAF | P15056 | H585Y | 1903 | ME2 | P23368 | E93D |
| 441 | BRAF | P15056 | I582M | 1904 | ME2 | P23368 | T283I |
| 442 | BRAF | P15056 | I582K | 1905 | ME2 | P23368 | R67G |
| 443 | BRAF | P15056 | L79M | 1906 | ME2 | P23368 | L169F |
| 444 | BRAF | P15056 | G518A | 1907 | ME2 | P23368 | D278N |
| 445 | CASP1 | P29466 | R391T | 1908 | ME2 | P23368 | I126L |
| 446 | CASP1 | P29466 | V338A | 1909 | ME2 | P23368 | G446C |
| 447 | CASP1 | P29466 | E241K | 1910 | ME2 | P23368 | R91T |
| 448 | CASP1 | P29466 | F173V | 1911 | ME2 | P23368 | R165K |
| 449 | CASP1 | P29466 | F234S | 1912 | ME2 | P23368 | I179S |
| 450 | CASP1 | P29466 | T388N | 1913 | ME2 | P23368 | L169I |
| 451 | CASP1 | P29466 | E390K | 1914 | ME2 | P23368 | A39T |
| 452 | CASP1 | P29466 | S339Y | 1915 | ME2 | P23368 | A39S |
| 453 | CASP1 | P29466 | S339F | 1916 | ME2 | P23368 | Q253H |
| 454 | CASP6 | P55212 | H58Y | 1917 | MIF | P14174 | M3I |
| 455 | CASP6 | P55212 | K99E | 1918 | MIF | P14174 | N98I |
| 456 | CASP6 | P55212 | K99T | 1919 | MIF | P14174 | Y99C |
| 457 | CASP6 | P55212 | Q274L | 1920 | MIF | P14174 | M102I |
| 458 | CASP6 | P55212 | R220W | 1921 | MIF | P14174 | P34S |
| 459 | CASP6 | P55212 | L98F | 1922 | MIF | P14174 | P2R |
| 460 | CASP6 | P55212 | L98H | 1923 | MIF | P14174 | G32D |
| 461 | CASP6 | P55212 | A279V | 1924 | MMP7 | P09237 | S225F |
| 462 | CASP6 | P55212 | H101R | 1925 | MMP7 | P09237 | M135I |
| 463 | CASP6 | P55212 | Y216C | 1926 | MMP7 | P09237 | H122P |
| 464 | CASP6 | P55212 | A195T | 1927 | MMP7 | P09237 | G137D |
| 465 | CASP6 | P55212 | A213T | 1928 | MMP7 | P09237 | R127L |
| 466 | CASP6 | P55212 | A34T | 1929 | MMP7 | P09237 | R127Q |
| 467 | CASP6 | P55212 | K285N | 1930 | MMP7 | P09237 | Y236F |
| 468 | CASP6 | P55212 | F56L | 1931 | MMP7 | P09237 | P121L |
| 469 | CASP6 | P55212 | Q230E | 1932 | MMP7 | P09237 | G223R |
| 470 | CASP6 | P55212 | T199M | 1933 | MMP7 | P09237 | H178Y |
| 471 | CASP6 | P55212 | S226L | 1934 | MMP7 | P09237 | F65L |
| 472 | CASP7 | P55210 | E274D | 1935 | MMP9 | P14780 | A417V |
| 473 | CASP7 | P55210 | E274Q | 1936 | MMP9 | P14780 | V398M |
| 474 | CASP7 | P55210 | S224F | 1937 | MMP9 | P14780 | L442I |
| 475 | CASP7 | P55210 | P235S | 1938 | MMP9 | P14780 | G100E |
| 476 | CASP7 | P55210 | K160E | 1939 | MMP9 | P14780 | G408V |
| 477 | CASP7 | P55210 | V149I | 1940 | MMP9 | P14780 | H190Y |
| 478 | CASP7 | P55210 | G236E | 1941 | MMP9 | P14780 | P194T |
| 479 | CASP7 | P55210 | Q276H | 1942 | MMP9 | P14780 | S394T |
| 480 | CASP7 | P55210 | R187Q | 1943 | MMP9 | P14780 | G186R |
| 481 | CASP7 | P55210 | E284D | 1944 | MMP9 | P14780 | P193S |
| 482 | CASP7 | P55210 | S234N | 1945 | MMP9 | P14780 | R51C |
| 483 | CASP7 | P55210 | S231L | 1946 | MMP9 | P14780 | D182N |
| 484 | CASP7 | P55210 | W232G | 1947 | MMP9 | P14780 | A406T |
| 485 | CASP7 | P55210 | D93H | 1948 | MMP9 | P14780 | A406V |
| 486 | CASP7 | P55210 | R210Q | 1949 | MMP9 | P14780 | G404C |
| 487 | CASP7 | P55210 | R87Q | 1950 | MMP9 | P14780 | G404D |
| 488 | CASP8 | Q14790 | V410A | 1951 | MMP9 | P14780 | R424L |
| 489 | CASP8 | Q14790 | T469I | 1952 | MMP9 | P14780 | L188M |
| 490 | CASP8 | Q14790 | A404V | 1953 | MMP9 | P14780 | A400T |
| 491 | CASP8 | Q14790 | W420C | 1954 | MMP9 | P14780 | A400E |
| 492 | CASP8 | Q14790 | S338F | 1955 | MMP9 | P14780 | M422I |
| 493 | CASP8 | Q14790 | S338C | 1956 | MSH2 | P43246 | M83I |
| 494 | CASP8 | Q14790 | S316F | 1957 | MSH2 | P43246 | S676L |
| 495 | CASP8 | Q14790 | N241S | 1958 | MSH2 | P43246 | I544M |
| 496 | CASP8 | Q14790 | N241K | 1959 | MSH2 | P43246 | K550T |
| 497 | CASP8 | Q14790 | F340C | 1960 | MSH2 | P43246 | T677R |
| 498 | CASP8 | Q14790 | L315V | 1961 | MSH2 | P43246 | N671I |
| 499 | CASP8 | Q14790 | D259Y | 1962 | MSH2 | P43246 | T526A |
| 500 | CASP8 | Q14790 | R413Q | 1963 | MSH2 | P43246 | Q510H |
| 501 | CASP8 | Q14790 | G342D | 1964 | MSH2 | P43246 | S676P |
| 502 | CASP8 | Q14790 | G342S | 1965 | MSH2 | P43246 | G673E |
| 503 | CASP8 | Q14790 | K353N | 1966 | MSH2 | P43246 | G673R |
| 504 | CASP8 | Q14790 | K353T | 1967 | MSH2 | P43246 | G548V |
| 505 | CASP8 | Q14790 | R260S | 1968 | MSH2 | P43246 | A517V |
| 506 | CASP8 | Q14790 | D455H | 1969 | MSH2 | P43246 | G669C |
| 507 | CASP8 | Q14790 | D266N | 1970 | MSH2 | P43246 | G669D |
| 508 | CASP8 | Q14790 | I333M | 1971 | MSH2 | P43246 | Q645E |
| 509 | CBS | P35520 | G256D | 1972 | MSH2 | P43246 | R524C |
| 510 | CBS | P35520 | S420L | 1973 | MSH2 | P43246 | R524H |
| 511 | CBS | P35520 | G305R | 1974 | MSH2 | P43246 | M813W |
| 512 | CBS | P35520 | A350S | 1975 | MSH2 | P43246 | M813T |
| 513 | CBS | P35520 | L287F | 1976 | MSH2 | P43246 | V802A |
| 514 | CBS | P35520 | A509T | 1977 | MSH6 | P52701 | W970C |
| 515 | CBS | P35520 | R439W | 1978 | MSH6 | P52701 | G448E |
| 516 | CBS | P35520 | V118M | 1979 | MSH6 | P52701 | W970L |
| 517 | CBS | P35520 | I537V | 1980 | MSH6 | P52701 | G430R |
| 518 | CBS | P35520 | G348V | 1981 | MSH6 | P52701 | G971E |
| 519 | CBS | P35520 | H507Q | 1982 | MSH6 | P52701 | A465T |
| 520 | CBS | P35520 | H507R | 1983 | MSH6 | P52701 | M452V |
| 521 | CBS | P35520 | I261T | 1984 | MSH6 | P52701 | E490K |
| 522 | CBS | P35520 | G347S | 1985 | MSH6 | P52701 | Y427D |
| 523 | CCR5 | P51681 | T288M | 1986 | MSH6 | P52701 | R468H |
| 524 | CCR5 | P51681 | T288A | 1987 | MSH6 | P52701 | E1023G |
| 525 | CCR5 | P51681 | Q194H | 1988 | MSH6 | P52701 | E980K |
| 526 | CCR5 | P51681 | K191N | 1989 | MSH6 | P52701 | K431T |
| 527 | CCR5 | P51681 | G286E | 1990 | MSH6 | P52701 | G443R |
| 528 | CCR5 | P51681 | F109Y | 1991 | MSH6 | P52701 | I464T |
| 529 | CCR5 | P51681 | N192I | 1992 | MSH6 | P52701 | K1000N |
| 530 | CCR5 | P51681 | T284N | 1993 | MSH6 | P52701 | M410I |
| 531 | CCR5 | P51681 | G106V | 1994 | MSH6 | P52701 | G409E |
| 532 | CCR5 | P51681 | T284S | 1995 | MSH6 | P52701 | I1019K |
| 533 | CCR5 | P51681 | N252S | 1996 | MSH6 | P52701 | V450A |
| 534 | CCR5 | P51681 | N258K | 1997 | MSH6 | P52701 | P408S |
| 535 | CCR5 | P51681 | F85L | 1998 | MSH6 | P52701 | E487D |
| 536 | CCR5 | P51681 | V83L | 1999 | MSH6 | P52701 | E487K |
| 537 | CCR5 | P51681 | G106R | 2000 | MSH6 | P52701 | K412T |
| 538 | CCR5 | P51681 | Q280L | 2001 | MSH6 | P52701 | P462L |
| 539 | CCR5 | P51681 | I110T | 2002 | MSH6 | P52701 | T1189I |
| 540 | CCR5 | P51681 | L104F | 2003 | MSH6 | P52701 | Q485H |
| 541 | CCR5 | P51681 | I198V | 2004 | MSH6 | P52701 | R411M |
| 542 | CDC34 | P49427 | I96T | 2005 | MSH6 | P52701 | V508M |
| 543 | CDC34 | P49427 | L130I | 2006 | MSH6 | P52701 | V508L |
| 544 | CDC34 | P49427 | S129F | 2007 | MSH6 | P52701 | Y469F |
| 545 | CDC34 | P49427 | T122I | 2008 | MTOR | P42345 | L2103I |
| 546 | CDC34 | P49427 | T122A | 2009 | MTOR | P42345 | R2339I |
| 547 | CDC34 | P49427 | L124F | 2010 | MTOR | P42345 | H2106Y |
| 548 | CDC34 | P49427 | P49L | 2011 | MTOR | P42345 | E2032K |
| 549 | CDK2 | P24941 | L175M | 2012 | MTOR | P42345 | R2224K |
| 550 | CDK2 | P24941 | G13D | 2013 | MTOR | P42345 | M2345I |
| 551 | CDK2 | P24941 | G13S | 2014 | MTOR | P42345 | G2359R |
| 552 | CDK2 | P24941 | E12A | 2015 | MTOR | P42345 | E2190K |
| 553 | CDK2 | P24941 | V164L | 2016 | MTOR | P42345 | V2240G |
| 554 | CDK2 | P24941 | V230F | 2017 | MTOR | P42345 | L2097F |
| 555 | CDK2 | P24941 | D270N | 2018 | MTOR | P42345 | G2030S |
| 556 | CDK2 | P24941 | K20E | 2019 | MTOR | P42345 | H2242R |
| 557 | CDK2 | P24941 | H125Y | 2020 | MTOR | P42345 | W2239C |
| 558 | CDK2 | P24941 | G13C | 2021 | MTOR | P42345 | Y2225C |
| 559 | CDK2 | P24941 | D68N | 2022 | MTOR | P42345 | H2355R |
| 560 | CDK2 | P24941 | P155S | 2023 | MTOR | P42345 | P2241S |
| 561 | CDK2 | P24941 | P155H | 2024 | MTOR | P42345 | I2356V |
| 562 | CDK2 | P24941 | S232Y | 2025 | MTOR | P42345 | I2356F |
| 563 | CDK2 | P24941 | K34T | 2026 | MTOR | P42345 | S2342Y |
| 564 | CDK2 | P24941 | K34N | 2027 | MTOR | P42345 | P2169S |
| 565 | CDK2 | P24941 | K89T | 2028 | MTOR | P42345 | G2359E |
| 566 | CDK2 | P24941 | K65R | 2029 | MTOR | P42345 | D2338N |
| 567 | CDK2 | P24941 | V163M | 2030 | MTOR | P42345 | R2036L |
| 568 | CDK2 | P24941 | H84Y | 2031 | MTOR | P42345 | R2036H |
| 569 | CHEK1 | O14757 | D130Y | 2032 | NT5C2 | P49902 | L287F |
| 570 | CHEK1 | O14757 | A200S | 2033 | NT5C2 | P49902 | S251R |
| 571 | CHEK1 | O14757 | G16V | 2034 | NT5C2 | P49902 | M436I |
| 572 | CHEK1 | O14757 | F93V | 2035 | NT5C2 | P49902 | R291Q |
| 573 | CHEK1 | O14757 | E134K | 2036 | NT5C2 | P49902 | R291W |
| 574 | CHEK1 | O14757 | E134G | 2037 | NT5C2 | P49902 | K111Q |
| 575 | CHEK1 | O14757 | E134D | 2038 | NT5C2 | P49902 | D206Y |
| 576 | CHEK1 | O14757 | E85D | 2039 | NT5C2 | P49902 | L214P |
| 577 | CHEK1 | O14757 | L136F | 2040 | NT5C2 | P49902 | R129M |
| 578 | CHEK1 | O14757 | G21R | 2041 | NT5C2 | P49902 | D252E |
| 579 | CHEK1 | O14757 | G89R | 2042 | NT5C2 | P49902 | W207L |
| 580 | CHEK1 | O14757 | A36T | 2043 | NT5C2 | P49902 | L119F |
| 581 | CHEK1 | O14757 | I39T | 2044 | NT5C2 | P49902 | M53R |
| 582 | CHEK1 | O14757 | M201V | 2045 | NT5C2 | P49902 | G355E |
| 583 | CHEK1 | O14757 | T14N | 2046 | NT5C2 | P49902 | N158S |
| 584 | CHRM2 | P08172 | V106I | 2047 | OPRD1 | P41143 | A94S |
| 585 | CHRM2 | P08172 | A191T | 2048 | OPRD1 | P41143 | D210N |
| 586 | CHRM2 | P08172 | T190M | 2049 | OPRD1 | P41143 | L125F |
| 587 | CHRM2 | P08172 | Q179H | 2050 | OPRD1 | P41143 | D95N |
| 588 | CHRM2 | P08172 | Q179P | 2051 | OPRD1 | P41143 | N314S |
| 589 | CHRM2 | P08172 | W427L | 2052 | OPRD1 | P41143 | V217M |
| 590 | CHRM2 | P08172 | M406I | 2053 | OPRD1 | P41143 | K108N |
| 591 | CHRM2 | P08172 | P415T | 2054 | OPRD1 | P41143 | V281I |
| 592 | CHRM2 | P08172 | E172D | 2055 | OPRD1 | P41143 | A305V |
| 593 | CHRM2 | P08172 | F181L | 2056 | OPRD1 | P41143 | A305T |
| 594 | CHRM2 | P08172 | E175K | 2057 | OPRD1 | P41143 | C273Y |
| 595 | CHRM2 | P08172 | Y430D | 2058 | OPRD1 | P41143 | A96V |
| 596 | CHRM2 | P08172 | Y80H | 2059 | OPRD1 | P41143 | H301Q |
| 597 | CHRM2 | P08172 | S182P | 2060 | OPRD1 | P41143 | D128N |
| 598 | CHRM2 | P08172 | A401V | 2061 | OPRD1 | P41143 | S135N |
| 599 | CHRM2 | P08172 | T411P | 2062 | OPRD1 | P41143 | A98V |
| 600 | CHRM2 | P08172 | T411I | 2063 | OPRD1 | P41143 | Y308C |
| 601 | CHRM2 | P08172 | I178N | 2064 | PADI4 | Q9UM07 | E411K |
| 602 | CHRM2 | P08172 | V171M | 2065 | PADI4 | Q9UM07 | E252D |
| 603 | CHRM2 | P08172 | F195L | 2066 | PADI4 | Q9UM07 | E378V |
| 604 | CHRM2 | P08172 | A194S | 2067 | PADI4 | Q9UM07 | P387T |
| 605 | CHRM2 | P08172 | S110L | 2068 | PADI4 | Q9UM07 | V152M |
| 606 | CHRM2 | P08172 | N404S | 2069 | PADI4 | Q9UM07 | S468F |
| 607 | CHRM2 | P08172 | P198A | 2070 | PADI4 | Q9UM07 | D345G |
| 608 | CHRM2 | P08172 | W89S | 2071 | PADI4 | Q9UM07 | P380S |
| 609 | CHRM2 | P08172 | T187P | 2072 | PADI4 | Q9UM07 | P380L |
| 610 | CHRM2 | P08172 | T399N | 2073 | PADI4 | Q9UM07 | F389V |
| 611 | CHRM2 | P08172 | L428I | 2074 | PADI4 | Q9UM07 | Y391N |
| 612 | CHRM2 | P08172 | S107R | 2075 | PADI4 | Q9UM07 | R374S |
| 613 | CHRM2 | P08172 | T170I | 2076 | PADI4 | Q9UM07 | N588S |
| 614 | CHRM2 | P08172 | V421L | 2077 | PADI4 | Q9UM07 | H471Q |
| 615 | CHRM2 | P08172 | W422L | 2078 | PADI4 | Q9UM07 | R394Q |
| 616 | CHRM2 | P08172 | S110P | 2079 | PADI4 | Q9UM07 | E474K |
| 617 | CHRM2 | P08172 | Y88S | 2080 | PADI4 | Q9UM07 | N585S |
| 618 | CHRM2 | P08172 | L102V | 2081 | PADI4 | Q9UM07 | K377N |
| 619 | CHRM2 | P08172 | S76F | 2082 | PADI4 | Q9UM07 | G470C |
| 620 | CHRM2 | P08172 | D103Y | 2083 | PADI4 | Q9UM07 | M352V |
| 621 | CHRM2 | P08172 | A194E | 2084 | PADI4 | Q9UM07 | R639K |
| 622 | CHRM2 | P08172 | S151T | 2085 | PADI4 | Q9UM07 | I381M |
| 623 | CHRM2 | P08172 | S151P | 2086 | PADI4 | Q9UM07 | R651T |
| 624 | CHRM2 | P08172 | P418L | 2087 | PADI4 | Q9UM07 | D350N |
| 625 | CHRM2 | P08172 | P418H | 2088 | PADI4 | Q9UM07 | L429I |
| 626 | CHRM2 | P08172 | P418T | 2089 | PADI4 | Q9UM07 | G646S |
| 627 | CHRM2 | P08172 | L102M | 2090 | PADI4 | Q9UM07 | V649E |
| 628 | CPS1 | P31327 | P563S | 2091 | PADI4 | Q9UM07 | M352I |
| 629 | CPS1 | P31327 | T1391M | 2092 | PADI4 | Q9UM07 | L429V |
| 630 | CPS1 | P31327 | P563L | 2093 | PADI4 | Q9UM07 | S406F |
| 631 | CPS1 | P31327 | R718T | 2094 | PADI4 | Q9UM07 | V472M |
| 632 | CPS1 | P31327 | H1195Q | 2095 | PAH | P00439 | E44D |
| 633 | CPS1 | P31327 | L1245I | 2096 | PAH | P00439 | E78D |
| 634 | CPS1 | P31327 | C711F | 2097 | PAH | P00439 | R270T |
| 635 | CPS1 | P31327 | N1437K | 2098 | PAH | P00439 | L62Q |
| 636 | CPS1 | P31327 | N1257K | 2099 | PAH | P00439 | I65L |
| 637 | CPS1 | P31327 | P1125H | 2100 | PAH | P00439 | F80I |
| 638 | CPS1 | P31327 | H1447D | 2101 | PAH | P00439 | S67C |
| 639 | CPS1 | P31327 | E1171Q | 2102 | PAH | P00439 | F331L |
| 640 | CPS1 | P31327 | G596C | 2103 | PAH | P00439 | S350F |
| 641 | CPS1 | P31327 | R547K | 2104 | PAH | P00439 | E280K |
| 642 | CPS1 | P31327 | E714D | 2105 | PAH | P00439 | D282G |
| 643 | CPS1 | P31327 | V622L | 2106 | PAH | P00439 | E43K |
| 644 | CPS1 | P31327 | R1089C | 2107 | PAH | P00439 | G352S |
| 645 | CPS1 | P31327 | R1089H | 2108 | PAH | P00439 | E381K |
| 646 | CPS1 | P31327 | A655V | 2109 | PAH | P00439 | H290Y |
| 647 | CPS1 | P31327 | R1453W | 2110 | PAH | P00439 | E286K |
| 648 | CPS1 | P31327 | T1443A | 2111 | PAH | P00439 | E330K |
| 649 | CPS1 | P31327 | M656K | 2112 | PAH | P00439 | E330D |
| 650 | CPS1 | P31327 | M656I | 2113 | PAH | P00439 | G346A |
| 651 | CPS1 | P31327 | M656V | 2114 | PAH | P00439 | K42E |
| 652 | CPS1 | P31327 | G628V | 2115 | PAH | P00439 | K42I |
| 653 | CPS1 | P31327 | R587H | 2116 | PAH | P00439 | E44K |
| 654 | CPS1 | P31327 | W1397C | 2117 | PAH | P00439 | E78K |
| 655 | CPS1 | P31327 | S663A | 2118 | PAH | P00439 | G289R |
| 656 | CPS1 | P31327 | R1174Q | 2119 | PAH | P00439 | V45D |
| 657 | CPS1 | P31327 | R721L | 2120 | PAH | P00439 | V45A |
| 658 | CPS1 | P31327 | R721Q | 2121 | PAH | P00439 | N61I |
| 659 | CPS1 | P31327 | S625P | 2122 | PAH | P00439 | L48F |
| 660 | CPS1 | P31327 | V584M | 2123 | PAH | P00439 | P279L |
| 661 | CPS1 | P31327 | R1317W | 2124 | PAH | P00439 | K274M |
| 662 | CPS1 | P31327 | E631Q | 2125 | PAH | P00439 | P69S |
| 663 | CPS1 | P31327 | A1173T | 2126 | PAH | P00439 | P69H |
| 664 | CPS1 | P31327 | E1177Q | 2127 | PAK1 | Q13153 | T406I |
| 665 | CPS1 | P31327 | K624T | 2128 | PAK1 | Q13153 | R421W |
| 666 | CPS1 | P31327 | E1175K | 2129 | PAK1 | Q13153 | F410L |
| 667 | CPS1 | P31327 | R1371Q | 2130 | PAK1 | Q13153 | T415N |
| 668 | CPS1 | P31327 | R1371W | 2131 | PAK1 | Q13153 | F379L |
| 669 | CPS1 | P31327 | R1371L | 2132 | PAK1 | Q13153 | F379C |
| 670 | CPS1 | P31327 | L1252F | 2133 | PAK1 | Q13153 | L405I |
| 671 | CPS1 | P31327 | A591S | 2134 | PAK1 | Q13153 | D332N |
| 672 | CPS1 | P31327 | R1453Q | 2135 | PAK1 | Q13153 | I312S |
| 673 | CPS1 | P31327 | P583A | 2136 | PAK1 | Q13153 | I312N |
| 674 | CPS1 | P31327 | R1262Q | 2137 | PAK1 | Q13153 | M301I |
| 675 | CPS1 | P31327 | D1396N | 2138 | PAK1 | Q13153 | V342F |
| 676 | CPS1 | P31327 | T660A | 2139 | PAK1 | Q13153 | R421Q |
| 677 | CPS1 | P31327 | H1202N | 2140 | PAK1 | Q13153 | S382L |
| 678 | CPS1 | P31327 | L1127V | 2141 | PAK1 | Q13153 | N394S |
| 679 | CPS1 | P31327 | A562S | 2142 | PAK1 | Q13153 | M424I |
| 680 | CPS1 | P31327 | N698S | 2143 | PAK1 | Q13153 | I298N |
| 681 | CPS1 | P31327 | R1129K | 2144 | PAK1 | Q13153 | G277A |
| 682 | CPS1 | P31327 | E1177G | 2145 | PAK1 | Q13153 | M319I |
| 683 | CPS1 | P31327 | H1447Y | 2146 | PAK1 | Q13153 | M319T |
| 684 | CPS1 | P31327 | H1447P | 2147 | PAK1 | Q13153 | D389Y |
| 685 | CPS1 | P31327 | R718I | 2148 | PAK1 | Q13153 | E315K |
| 686 | CPS1 | P31327 | M792I | 2149 | PAK1 | Q13153 | N326H |
| 687 | CPS1 | P31327 | W1410L | 2150 | PANK1 | Q8TE04 | G340E |
| 688 | CPS1 | P31327 | G1204E | 2151 | PANK1 | Q8TE04 | G435V |
| 689 | CPS1 | P31327 | G661C | 2152 | PANK1 | Q8TE04 | A492V |
| 690 | CPS1 | P31327 | Q1368K | 2153 | PANK1 | Q8TE04 | K476E |
| 691 | CPS1 | P31327 | S564N | 2154 | PANK1 | Q8TE04 | F486C |
| 692 | CPS1 | P31327 | K1168T | 2155 | PANK1 | Q8TE04 | T521I |
| 693 | CPS1 | P31327 | I713S | 2156 | PANK1 | Q8TE04 | S491A |
| 694 | CPS1 | P31327 | T544M | 2157 | PANK1 | Q8TE04 | N525D |
| 695 | CPS1 | P31327 | S1203L | 2158 | PANK1 | Q8TE04 | N547H |
| 696 | CSNK2A1 | P68400 | L173I | 2159 | PANK1 | Q8TE04 | R432T |
| 697 | CSNK2A1 | P68400 | G46S | 2160 | PANK3 | Q9H999 | N299S |
| 698 | CSNK2A1 | P68400 | D175H | 2161 | PANK3 | Q9H999 | N299H |
| 699 | CSNK2A1 | P68400 | L45S | 2162 | PANK3 | Q9H999 | Y356N |
| 700 | CSNK2A1 | P68400 | N117K | 2163 | PANK3 | Q9H999 | R325H |
| 701 | CSNK2A1 | P68400 | H160R | 2164 | PANK3 | Q9H999 | R207Q |
| 702 | CSNK2A1 | P68400 | F113V | 2165 | PANK3 | Q9H999 | R251S |
| 703 | CSNK2A1 | P68400 | L70I | 2166 | PANK3 | Q9H999 | R251H |
| 704 | CSNK2A1 | P68400 | F54C | 2167 | PANK3 | Q9H999 | W266L |
| 705 | CSNK2A1 | P68400 | R172Q | 2168 | PANK3 | Q9H999 | E354G |
| 706 | CSNK2A1 | P68400 | D37V | 2169 | PANK3 | Q9H999 | D247N |
| 707 | CSNK2A1 | P68400 | V116I | 2170 | PANK3 | Q9H999 | G255E |
| 708 | CSNK2A1 | P68400 | V116A | 2171 | PANK3 | Q9H999 | G210E |
| 709 | CSNK2A1 | P68400 | T119K | 2172 | PANK3 | Q9H999 | F323L |
| 710 | CSNK2A1 | P68400 | A110T | 2173 | PANK3 | Q9H999 | L263F |
| 711 | CTSK | P43235 | A234T | 2174 | PANK3 | Q9H999 | I190F |
| 712 | CTSK | P43235 | D117Y | 2175 | PANK3 | Q9H999 | R325C |
| 713 | CTSK | P43235 | R241Q | 2176 | PANK3 | Q9H999 | G216V |
| 714 | CTSK | P43235 | L274V | 2177 | PANK3 | Q9H999 | G216D |
| 715 | CTSK | P43235 | Y283N | 2178 | PANK3 | Q9H999 | A118G |
| 716 | CTSK | P43235 | E232D | 2179 | PANK3 | Q9H999 | G210W |
| 717 | CTSK | P43235 | V278I | 2180 | PARP14 | Q460N5 | A1145T |
| 718 | CTSK | P43235 | G137R | 2181 | PARP14 | Q460N5 | V934G |
| 719 | CTSK | P43235 | R312Q | 2182 | PARP14 | Q460N5 | N824S |
| 720 | CTSK | P43235 | Q133E | 2183 | PARP14 | Q460N5 | A822G |
| 721 | CTSK | P43235 | G113C | 2184 | PARP14 | Q460N5 | V967D |
| 722 | CTSK | P43235 | G178V | 2185 | PARP14 | Q460N5 | A1128V |
| 723 | CTSK | P43235 | A248T | 2186 | PARP14 | Q460N5 | E1151D |
| 724 | CYP3A4 | P08684 | A370V | 2187 | PARP14 | Q460N5 | G1135E |
| 725 | CYP3A4 | P08684 | A448D | 2188 | PARP14 | Q460N5 | A1131S |
| 726 | CYP3A4 | P08684 | G436R | 2189 | PARP14 | Q460N5 | S963F |
| 727 | CYP3A4 | P08684 | F219L | 2190 | PARP14 | Q460N5 | E825K |
| 728 | CYP3A4 | P08684 | R212K | 2191 | PARP14 | Q460N5 | A836V |
| 729 | CYP3A4 | P08684 | M452V | 2192 | PARP14 | Q460N5 | R884C |
| 730 | CYP3A4 | P08684 | M452I | 2193 | PDE10A | Q9Y233 | S301L |
| 731 | CYP3A4 | P08684 | K209E | 2194 | PDE10A | Q9Y233 | V356L |
| 732 | CYP3A4 | P08684 | F213I | 2195 | PDE10A | Q9Y233 | P350T |
| 733 | CYP3A4 | P08684 | R440K | 2196 | PDE10A | Q9Y233 | V356A |
| 734 | CYP3A4 | P08684 | P434S | 2197 | PDE10A | Q9Y233 | P350A |
| 735 | CYP3A4 | P08684 | R130Q | 2198 | PDE10A | Q9Y233 | D305N |
| 736 | CYP3A4 | P08684 | P368S | 2199 | PDE10A | Q9Y233 | D624Y |
| 737 | CYP3A4 | P08684 | P218L | 2200 | PDE10A | Q9Y233 | D624N |
| 738 | CYP3A4 | P08684 | I301T | 2201 | PDE10A | Q9Y233 | L665V |
| 739 | CYP3A4 | P08684 | R446K | 2202 | PDE10A | Q9Y233 | A724T |
| 740 | CYP3A4 | P08684 | F137S | 2203 | PDE10A | Q9Y233 | R351H |
| 741 | CYP3A4 | P08684 | R212I | 2204 | PDE10A | Q9Y233 | M703V |
| 742 | CYP3A4 | P08684 | F241L | 2205 | PDE10A | Q9Y233 | G329R |
| 743 | CYP3A4 | P08684 | P242S | 2206 | PDE10A | Q9Y233 | F290Y |
| 744 | CYP3A4 | P08684 | S119F | 2207 | PDE10A | Q9Y233 | A626S |
| 745 | CYP3A4 | P08684 | L449I | 2208 | PDE10A | Q9Y233 | G329E |
| 746 | CYP3A4 | P08684 | A305V | 2209 | PDE10A | Q9Y233 | G332C |
| 747 | CYP3A4 | P08684 | S119P | 2210 | PDE10A | Q9Y233 | E685G |
| 748 | EED | O75530 | I363T | 2211 | PDE10A | Q9Y233 | V334L |
| 749 | EED | O75530 | G167V | 2212 | PDE10A | Q9Y233 | T678M |
| 750 | EED | O75530 | M366I | 2213 | PDE10A | Q9Y233 | G715C |
| 751 | EED | O75530 | Y308C | 2214 | PDE10A | Q9Y233 | Q333K |
| 752 | EED | O75530 | Y308F | 2215 | PDE10A | Q9Y233 | T661S |
| 753 | EED | O75530 | T149S | 2216 | PDE10A | Q9Y233 | Q716L |
| 754 | EED | O75530 | R414P | 2217 | PDE10A | Q9Y233 | E689D |
| 755 | EED | O75530 | I363V | 2218 | PDE10A | Q9Y233 | K279N |
| 756 | EED | O75530 | A412T | 2219 | PDE10A | Q9Y233 | K279T |
| 757 | EED | O75530 | R414Q | 2220 | PDE10A | Q9Y233 | K279Q |
| 758 | EIF4E | P06730 | V149A | 2221 | PDE10A | Q9Y233 | K279R |
| 759 | EIF4E | P06730 | F48S | 2222 | PDE10A | Q9Y233 | H557Y |
| 760 | EIF4E | P06730 | D90G | 2223 | PDE10A | Q9Y233 | S561N |
| 761 | EIF4E | P06730 | T68N | 2224 | PDE10A | Q9Y233 | T364M |
| 762 | EIF4E | P06730 | A74V | 2225 | PDE10A | Q9Y233 | L665I |
| 763 | EIF4E | P06730 | R61Q | 2226 | PDE10A | Q9Y233 | A679T |
| 764 | EIF4E | P06730 | S64F | 2227 | PDE10A | Q9Y233 | M704I |
| 765 | EIF4E | P06730 | W43C | 2228 | PDE10A | Q9Y233 | A288T |
| 766 | EIF4E | P06730 | W46C | 2229 | PDE10A | Q9Y233 | S561I |
| 767 | EIF4E | P06730 | A58P | 2230 | PDE10A | Q9Y233 | R286C |
| 768 | EIF4E | P06730 | L137H | 2231 | PDE10A | Q9Y233 | W752L |
| 769 | EIF4E | P06730 | G88C | 2232 | PDE10A | Q9Y233 | D554N |
| 770 | EIF4E | P06730 | W46L | 2233 | PDE10A | Q9Y233 | V381A |
| 771 | EIF4E | P06730 | S82C | 2234 | PDE10A | Q9Y233 | G718V |
| 772 | EIF4E | P06730 | R128H | 2235 | PDE10A | Q9Y233 | G718E |
| 773 | EIF4E | P06730 | R128C | 2236 | PDE10A | Q9Y233 | Y347N |
| 774 | EIF4E | P06730 | E70K | 2237 | PDE10A | Q9Y233 | C369S |
| 775 | EIF4E | P06730 | D51Y | 2238 | PDE10A | Q9Y233 | I620F |
| 776 | EIF4E | P06730 | F47L | 2239 | PDE10A | Q9Y233 | T675K |
| 777 | EIF4E | P06730 | R186K | 2240 | PDE10A | Q9Y233 | C666R |
| 778 | EIF4E | P06730 | E140Q | 2241 | PDE10A | Q9Y233 | N680S |
| 779 | EIF4E | P06730 | P38S | 2242 | PDE10A | Q9Y233 | I322M |
| 780 | EIF4E | P06730 | D51G | 2243 | PDE10A | Q9Y233 | L358M |
| 781 | EIF4E | P06730 | I35V | 2244 | PDE10A | Q9Y233 | R365L |
| 782 | EPAS1 | Q99814 | P334S | 2245 | PDE10A | Q9Y233 | R365W |
| 783 | EPAS1 | Q99814 | V317M | 2246 | PDE10A | Q9Y233 | W752C |
| 784 | EPAS1 | Q99814 | M338I | 2247 | PDE10A | Q9Y233 | T363I |
| 785 | EPAS1 | Q99814 | H248N | 2248 | PDE10A | Q9Y233 | F719L |
| 786 | EPAS1 | Q99814 | I261V | 2249 | PDE5A | O76074 | D803N |
| 787 | EPAS1 | Q99814 | L344M | 2250 | PDE5A | O76074 | L804V |
| 788 | EPAS1 | Q99814 | V343I | 2251 | PDE5A | O76074 | A783V |
| 789 | EPAS1 | Q99814 | S276L | 2252 | PDE5A | O76074 | F787C |
| 790 | EPAS1 | Q99814 | D258N | 2253 | PDE5A | O76074 | W853R |
| 791 | EPAS1 | Q99814 | Y342H | 2254 | PDE5A | O76074 | Q776K |
| 792 | EPAS1 | Q99814 | R275H | 2255 | PDE5A | O76074 | F786L |
| 793 | EPAS1 | Q99814 | E279V | 2256 | PDE5A | O76074 | F786C |
| 794 | EPAS1 | Q99814 | G323E | 2257 | PDE5A | O76074 | I778T |
| 795 | EPAS1 | Q99814 | A277P | 2258 | PDE5A | O76074 | I813V |
| 796 | EPAS1 | Q99814 | T324M | 2259 | PDE5A | O76074 | D654N |
| 797 | EPAS1 | Q99814 | R260I | 2260 | PDK2 | Q15119 | E56Q |
| 798 | EPAS1 | Q99814 | R308Q | 2261 | PDK2 | Q15119 | E56K |
| 799 | EPAS1 | Q99814 | K291N | 2262 | PDK2 | Q15119 | I169M |
| 800 | EPAS1 | Q99814 | T321I | 2263 | PDK2 | Q15119 | S333F |
| 801 | EPAS1 | Q99814 | T321A | 2264 | PDK2 | Q15119 | L174P |
| 802 | EPAS1 | Q99814 | Q306L | 2265 | PDK2 | Q15119 | I175V |
| 803 | EPAS1 | Q99814 | Q306H | 2266 | PDK2 | Q15119 | T51P |
| 804 | EPAS1 | Q99814 | M289V | 2267 | PDK2 | Q15119 | R60L |
| 805 | EPAS1 | Q99814 | M289I | 2268 | PDK2 | Q15119 | R60C |
| 806 | EPAS1 | Q99814 | M250L | 2269 | PDK2 | Q15119 | A259V |
| 807 | EPAS1 | Q99814 | L272R | 2270 | PDK2 | Q15119 | S29F |
| 808 | ESR2 | Q92731 | V307A | 2271 | PDK2 | Q15119 | E46K |
| 809 | ESR2 | Q92731 | D326G | 2272 | PDK2 | Q15119 | L57M |
| 810 | ESR2 | Q92731 | A313T | 2273 | PDK2 | Q15119 | R166H |
| 811 | ESR2 | Q92731 | R346C | 2274 | PDPK1 | O15530 | T95M |
| 812 | ESR2 | Q92731 | D326H | 2275 | PDPK1 | O15530 | L88F |
| 813 | ESR2 | Q92731 | D326N | 2276 | PDPK1 | O15530 | L88V |
| 814 | ESR2 | Q92731 | L374P | 2277 | PFKP | Q01813 | V418G |
| 815 | ESR2 | Q92731 | E337V | 2278 | PFKP | Q01813 | S640R |
| 816 | ESR2 | Q92731 | G342E | 2279 | PFKP | Q01813 | E140D |
| 817 | ESR2 | Q92731 | G372E | 2280 | PFKP | Q01813 | S32R |
| 818 | ESR2 | Q92731 | L322I | 2281 | PFKP | Q01813 | D128N |
| 819 | ESR2 | Q92731 | V320M | 2282 | PFKP | Q01813 | R576C |
| 820 | ESR2 | Q92731 | D303N | 2283 | PFKP | Q01813 | G34R |
| 821 | ESR2 | Q92731 | R501C | 2284 | PFKP | Q01813 | T103M |
| 822 | ESR2 | Q92731 | V370I | 2285 | PFKP | Q01813 | A537T |
| 823 | ESR2 | Q92731 | R329Q | 2286 | PFKP | Q01813 | R665K |
| 824 | ESR2 | Q92731 | R329W | 2287 | PFKP | Q01813 | R665M |
| 825 | ESR2 | Q92731 | A302V | 2288 | PFKP | Q01813 | S130I |
| 826 | ESR2 | Q92731 | L354F | 2289 | PFKP | Q01813 | H671R |
| 827 | EZH2 | Q15910 | I708T | 2290 | PFKP | Q01813 | R481H |
| 828 | EZH2 | Q15910 | F667L | 2291 | PFKP | Q01813 | D663N |
| 829 | EZH2 | Q15910 | A687V | 2292 | PFKP | Q01813 | L131I |
| 830 | EZH2 | Q15910 | I684S | 2293 | PFKP | Q01813 | R481C |
| 831 | F2 | P00734 | R543L | 2294 | PFKP | Q01813 | G33S |
| 832 | F2 | P00734 | R543H | 2295 | PFKP | Q01813 | G129W |
| 833 | F2 | P00734 | L459Q | 2296 | PFKP | Q01813 | D177Y |
| 834 | F2 | P00734 | R456Q | 2297 | PFKP | Q01813 | S173Y |
| 835 | F2 | P00734 | A551V | 2298 | PFKP | Q01813 | S173C |
| 836 | F2 | P00734 | S589P | 2299 | PFKP | Q01813 | G129E |
| 837 | F2 | P00734 | G569V | 2300 | PFKP | Q01813 | A100D |
| 838 | F2 | P00734 | V588I | 2301 | PFKP | Q01813 | F509S |
| 839 | F2 | P00734 | I542M | 2302 | PFKP | Q01813 | F509I |
| 840 | F2 | P00734 | D597G | 2303 | PFKP | Q01813 | D128G |
| 841 | F2 | P00734 | L459P | 2304 | PFKP | Q01813 | R219Q |
| 842 | F2 | P00734 | L390M | 2305 | PFKP | Q01813 | A537S |
| 843 | F2 | P00734 | G570A | 2306 | PFKP | Q01813 | G133R |
| 844 | F2 | P00734 | E509K | 2307 | PFKP | Q01813 | H208D |
| 845 | F2 | P00734 | A405T | 2308 | PFKP | Q01813 | G129R |
| 846 | F2 | P00734 | G570R | 2309 | PFKP | Q01813 | R576H |
| 847 | F2 | P00734 | G561E | 2310 | PFKP | Q01813 | A96V |
| 848 | F7 | P08709 | A266V | 2311 | PFKP | Q01813 | D175Y |
| 849 | F7 | P08709 | A266T | 2312 | PFKP | Q01813 | D175N |
| 850 | F7 | P08709 | D256E | 2313 | PFKP | Q01813 | N542S |
| 851 | F7 | P08709 | A235D | 2314 | PFKP | Q01813 | F578L |
| 852 | F7 | P08709 | R350C | 2315 | PKLR | P30613 | R479C |
| 853 | F7 | P08709 | R350S | 2316 | PKLR | P30613 | R479H |
| 854 | F7 | P08709 | A251E | 2317 | PKLR | P30613 | E161D |
| 855 | F7 | P08709 | A251V | 2318 | PKLR | P30613 | G478A |
| 856 | F7 | P08709 | A251T | 2319 | PKLR | P30613 | A336T |
| 857 | F7 | P08709 | A390T | 2320 | PKLR | P30613 | A336E |
| 858 | F7 | P08709 | A390V | 2321 | PKLR | P30613 | D528Y |
| 859 | F7 | P08709 | G234R | 2322 | PKLR | P30613 | T475I |
| 860 | F7 | P08709 | R284L | 2323 | PKLR | P30613 | A370V |
| 861 | F7 | P08709 | G278E | 2324 | PKLR | P30613 | G563S |
| 862 | F7 | P08709 | G278W | 2325 | PKLR | P30613 | R498H |
| 863 | F7 | P08709 | G278R | 2326 | PKLR | P30613 | K158Q |
| 864 | F7 | P08709 | D302N | 2327 | PKLR | P30613 | G338E |
| 865 | F7 | P08709 | G269S | 2328 | PKLR | P30613 | K158N |
| 866 | F7 | P08709 | Q236R | 2329 | PKLR | P30613 | R559Q |
| 867 | F7 | P08709 | E270K | 2330 | PKM | P14618 | R455Q |
| 868 | F7 | P08709 | L348P | 2331 | PKM | P14618 | V453M |
| 869 | F7 | P08709 | D277N | 2332 | PKM | P14618 | L465M |
| 870 | F7 | P08709 | L273F | 2333 | PKM | P14618 | G298C |
| 871 | F7 | P08709 | W344L | 2334 | PKM | P14618 | R106W |
| 872 | F7 | P08709 | V232M | 2335 | PKM | P14618 | R106Q |
| 873 | F7 | P08709 | L229M | 2336 | PKM | P14618 | A293G |
| 874 | FBP1 | P09467 | M249I | 2337 | PKM | P14618 | A242V |
| 875 | FBP1 | P09467 | A251T | 2338 | PKM | P14618 | R516H |
| 876 | FBP1 | P09467 | E21K | 2339 | PKM | P14618 | R516C |
| 877 | FBP1 | P09467 | R255H | 2340 | PKM | P14618 | M69I |
| 878 | FBP1 | P09467 | L176F | 2341 | PKM | P14618 | M291I |
| 879 | FBP1 | P09467 | V246A | 2342 | PKM | P14618 | R500Q |
| 880 | FBP1 | P09467 | E30K | 2343 | PKM | P14618 | L398H |
| 881 | FBP1 | P09467 | M178V | 2344 | PKM | P14618 | G468V |
| 882 | FBP1 | P09467 | Y216C | 2345 | PKM | P14618 | T432M |
| 883 | FBP1 | P09467 | R141I | 2346 | PKM | P14618 | N350S |
| 884 | FBP1 | P09467 | K275R | 2347 | PKM | P14618 | R73H |
| 885 | FBP1 | P09467 | A25T | 2348 | PKM | P14618 | G46V |
| 886 | FBP1 | P09467 | R141G | 2349 | PKM | P14618 | T513I |
| 887 | FBP1 | P09467 | I209F | 2350 | PKM | P14618 | D354N |
| 888 | FBP2 | O00757 | R277W | 2351 | PKM | P14618 | R342L |
| 889 | FBP2 | O00757 | R16C | 2352 | PKM | P14618 | R342W |
| 890 | FBP2 | O00757 | S125F | 2353 | PKM | P14618 | F307C |
| 891 | FBP2 | O00757 | K275N | 2354 | PKM | P14618 | L465P |
| 892 | FBP2 | O00757 | G215D | 2355 | PKM | P14618 | R43P |
| 893 | FBP2 | O00757 | G29W | 2356 | PKM | P14618 | P107L |
| 894 | FBP2 | O00757 | R16H | 2357 | PKM | P14618 | R489W |
| 895 | FBP2 | O00757 | S177A | 2358 | PKM | P14618 | R467C |
| 896 | FBP2 | O00757 | N126T | 2359 | PKM | P14618 | E272K |
| 897 | FBP2 | O00757 | R314Q | 2360 | PKM | P14618 | S519F |
| 898 | FBP2 | O00757 | L176F | 2361 | PKM | P14618 | H464R |
| 899 | FBP2 | O00757 | V253M | 2362 | PPARG | P37231 | M357I |
| 900 | FBP2 | O00757 | V161M | 2363 | PPARG | P37231 | R308C |
| 901 | FBP2 | O00757 | S177F | 2364 | PPARG | P37231 | G374V |
| 902 | FBP2 | O00757 | V246A | 2365 | PPARG | P37231 | S282F |
| 903 | FBP2 | O00757 | K21R | 2366 | PPARG | P37231 | P387R |
| 904 | FBP2 | O00757 | V246L | 2367 | PPARG | P37231 | F310S |
| 905 | FBP2 | O00757 | G179E | 2368 | PPARG | P37231 | I369L |
| 906 | FBP2 | O00757 | E281K | 2369 | PPARG | P37231 | G366E |
| 907 | FBP2 | O00757 | I139M | 2370 | PPARG | P37231 | C313Y |
| 908 | FBP2 | O00757 | T28S | 2371 | PPARG | P37231 | F315Y |
| 909 | FBP2 | O00757 | S271I | 2372 | PPARG | P37231 | E476G |
| 910 | FBP2 | O00757 | R23H | 2373 | PPARG | P37231 | E323K |
| 911 | FBP2 | O00757 | R23C | 2374 | PPARG | P37231 | E323D |
| 912 | FBP2 | O00757 | S132C | 2375 | PPARG | P37231 | E319D |
| 913 | FBP2 | O00757 | A131T | 2376 | PRKAA2 | P54646 | E100A |
| 914 | FCGR3A | P08637 | R115W | 2377 | PRKAA2 | P54646 | G159V |
| 915 | FCGR3A | P08637 | L136M | 2378 | PRKAA2 | P54646 | K45R |
| 916 | FCGR3A | P08637 | W34R | 2379 | PRKAA2 | P54646 | E143D |
| 917 | FCGR3A | P08637 | G85R | 2380 | PRKAA2 | P54646 | V17M |
| 918 | FCGR3A | P08637 | R36K | 2381 | PRKAA2 | P54646 | S97F |
| 919 | FCGR3A | P08637 | G107C | 2382 | PRKAA2 | P54646 | V24F |
| 920 | FCGR3A | P08637 | G107S | 2383 | PRKAA2 | P54646 | I46L |
| 921 | FCGR3A | P08637 | R88G | 2384 | PRKAA2 | P54646 | R10Q |
| 922 | FCGR3A | P08637 | R88S | 2385 | PRKAA2 | P54646 | R10L |
| 923 | FCGR3A | P08637 | F151Y | 2386 | PRKAA2 | P54646 | N144I |
| 924 | FCGR3A | P08637 | V181L | 2387 | PRKAA2 | P54646 | G25D |
| 925 | FCGR3A | P08637 | L175I | 2388 | PRKAA2 | P54646 | F102S |
| 926 | FCGR3A | P08637 | L38F | 2389 | PRKAA2 | P54646 | G28S |
| 927 | FCGR3A | P08637 | Y87C | 2390 | PRKAA2 | P54646 | E94G |
| 928 | FCGR3A | P08637 | I190M | 2391 | PRKAA2 | P54646 | E94K |
| 929 | FCGR3A | P08637 | S169A | 2392 | PRKAA2 | P54646 | G159E |
| 930 | FCGR3A | P08637 | Q33H | 2393 | PRKAA2 | P54646 | G159R |
| 931 | FCGR3A | P08637 | G168D | 2394 | PRKAA2 | P54646 | V42L |
| 932 | FCGR3A | P08637 | L102H | 2395 | PRKAA2 | P54646 | I77M |
| 933 | FCGR3A | P08637 | L102R | 2396 | PRKAA2 | P54646 | M91V |
| 934 | FDPS | P14324 | Y265F | 2397 | PRKAA2 | P54646 | D88Y |
| 935 | FDPS | P14324 | F304L | 2398 | PRKAA2 | P54646 | K51M |
| 936 | FDPS | P14324 | G322R | 2399 | PRKAA2 | P54646 | D157N |
| 937 | FDPS | P14324 | G322S | 2400 | PRKAB1 | Q9Y478 | N110I |
| 938 | FDPS | P14324 | G322D | 2401 | PRKAB1 | Q9Y478 | N110S |
| 939 | FDPS | P14324 | R178C | 2402 | PRKAB1 | Q9Y478 | R83P |
| 940 | FDPS | P14324 | R178H | 2403 | PRKAB1 | Q9Y478 | N111K |
| 941 | FDPS | P14324 | D240Y | 2404 | PRKAG1 | P54619 | G311R |
| 942 | FDPS | P14324 | I262V | 2405 | PRKAG1 | P54619 | H151Y |
| 943 | FDPS | P14324 | K332T | 2406 | PRKAG1 | P54619 | Q320K |
| 944 | FDPS | P14324 | I324V | 2407 | PRKAG1 | P54619 | I204F |
| 945 | FDPS | P14324 | G121E | 2408 | PRKAG1 | P54619 | R299Q |
| 946 | FDPS | P14324 | K323T | 2409 | PRKAG1 | P54619 | V313L |
| 947 | FDPS | P14324 | K323N | 2410 | PRKAG1 | P54619 | Y241C |
| 948 | FDPS | P14324 | E384K | 2411 | PRKAG1 | P54619 | V225I |
| 949 | FDPS | P14324 | Y124C | 2412 | PRKAG1 | P54619 | I219V |
| 950 | FDPS | P14324 | F272L | 2413 | PRKAG1 | P54619 | G199D |
| 951 | FDPS | P14324 | P275A | 2414 | PRMT3 | O60678 | R239Q |
| 952 | FDPS | P14324 | R412C | 2415 | PRMT3 | O60678 | V373M |
| 953 | FDPS | P14324 | Y415H | 2416 | PRMT3 | O60678 | I313T |
| 954 | FDPS | P14324 | D173A | 2417 | PRMT3 | O60678 | V406G |
| 955 | FDPS | P14324 | A411V | 2418 | PRMT3 | O60678 | D285G |
| 956 | FDPS | P14324 | T233S | 2419 | PRMT3 | O60678 | H479Q |
| 957 | FDPS | P14324 | F305L | 2420 | PRMT3 | O60678 | R379I |
| 958 | FKBP1A | P62942 | S39Y | 2421 | PRMT3 | O60678 | A377V |
| 959 | FKBP1A | P62942 | S39C | 2422 | PRMT3 | O60678 | W383C |
| 960 | FKBP1A | P62942 | E61K | 2423 | PRMT3 | O60678 | T449M |
| 961 | FKBP1A | P62942 | R58Q | 2424 | PRMT3 | O60678 | Y243H |
| 962 | FKBP1A | P62942 | F100L | 2425 | PRMT3 | O60678 | I380T |
| 963 | FKBP1A | P62942 | F47L | 2426 | PRMT3 | O60678 | R513C |
| 964 | FKBP1A | P62942 | A82V | 2427 | PRMT3 | O60678 | G265V |
| 965 | GBA | P04062 | N421I | 2428 | PRMT3 | O60678 | W480G |
| 966 | GBA | P04062 | P161L | 2429 | PRMT3 | O60678 | G311V |
| 967 | GBA | P04062 | L280F | 2430 | PRMT3 | O60678 | E231A |
| 968 | GBA | P04062 | V437F | 2431 | PRPS1 | P60891 | L145V |
| 969 | GBA | P04062 | Q401L | 2432 | PRPS1 | P60891 | V219G |
| 970 | GBA | P04062 | N435K | 2433 | PRPS1 | P60891 | R96Q |
| 971 | GCGR | P47871 | K344N | 2434 | PRPS1 | P60891 | R96W |
| 972 | GCGR | P47871 | R346Q | 2435 | PRPS1 | P60891 | F313L |
| 973 | GCGR | P47871 | L357V | 2436 | PRPS1 | P60891 | N144S |
| 974 | GCGR | P47871 | V292I | 2437 | PRPS1 | P60891 | N144K |
| 975 | GCGR | P47871 | E133K | 2438 | PRPS1 | P60891 | G174R |
| 976 | GCGR | P47871 | D209N | 2439 | PRPS1 | P60891 | S103N |
| 977 | GCGR | P47871 | R308W | 2440 | PRPS1 | P60891 | S103T |
| 978 | GCGR | P47871 | Y145H | 2441 | PRPS1 | P60891 | S103R |
| 979 | GCGR | P47871 | E406D | 2442 | PRPS1 | P60891 | A105V |
| 980 | GCGR | P47871 | E406K | 2443 | PRPS1 | P60891 | T126I |
| 981 | GCGR | P47871 | A380T | 2444 | PRPS1 | P60891 | D221G |
| 982 | GCGR | P47871 | L198I | 2445 | PRPS1 | P60891 | D101Y |
| 983 | GCGR | P47871 | R308Q | 2446 | PTPN1 | P18031 | G220S |
| 984 | GCGR | P47871 | K205T | 2447 | PTPN1 | P18031 | G220D |
| 985 | GCGR | P47871 | D30H | 2448 | PTPN1 | P18031 | H214P |
| 986 | GCGR | P47871 | R173H | 2449 | PTPN1 | P18031 | V198F |
| 987 | GCK | P35557 | D160Y | 2450 | PTPN1 | P18031 | M282T |
| 988 | GCK | P35557 | D160N | 2451 | PTPN1 | P18031 | I219T |
| 989 | GCK | P35557 | Y215F | 2452 | PTPN1 | P18031 | Q85H |
| 990 | GCK | P35557 | Y215N | 2453 | PTPN1 | P18031 | S222A |
| 991 | GCK | P35557 | T65I | 2454 | PTPN1 | P18031 | D181A |
| 992 | GCK | P35557 | A450V | 2455 | PTPN1 | P18031 | R257Q |
| 993 | GCK | P35557 | D205N | 2456 | PTPN1 | P18031 | W179R |
| 994 | GCK | P35557 | A449V | 2457 | PTPN1 | P18031 | Y46H |
| 995 | GCK | P35557 | G68S | 2458 | PTPN1 | P18031 | E200K |
| 996 | GCK | P35557 | R63H | 2459 | PTPN1 | P18031 | A217T |
| 997 | GCK | P35557 | G81V | 2460 | PTPN1 | P18031 | R257W |
| 998 | GCK | P35557 | A259T | 2461 | PTPN1 | P18031 | D48Y |
| 999 | GCK | P35557 | M251L | 2462 | PTPN1 | P18031 | M258I |
| 1000 | GCK | P35557 | G227D | 2463 | PTPN11 | Q06124 | T219A |
| 1001 | GCK | P35557 | A450T | 2464 | PTPN11 | Q06124 | K55N |
| 1002 | GCK | P35557 | E221K | 2465 | PTPN11 | Q06124 | T42A |
| 1003 | GCK | P35557 | S64F | 2466 | PTPN11 | Q06124 | E110G |
| 1004 | GCK | P35557 | V222I | 2467 | PTPN11 | Q06124 | L77V |
| 1005 | GCK | P35557 | D217N | 2468 | PTPN11 | Q06124 | V78I |
| 1006 | GCK | P35557 | G229D | 2469 | PTPN11 | Q06124 | N37T |
| 1007 | GLRA3 | O75311 | L160I | 2470 | PTPN11 | Q06124 | R265Q |
| 1008 | GLRA3 | O75311 | L160F | 2471 | PTPN11 | Q06124 | P107F |
| 1009 | GLRA3 | O75311 | I267M | 2472 | PTPN11 | Q06124 | H53Q |
| 1010 | GLRA3 | O75311 | S311A | 2473 | PTPN11 | Q06124 | H85Y |
| 1011 | GLRA3 | O75311 | R98H | 2474 | PTPN11 | Q06124 | Y279C |
| 1012 | GLRA3 | O75311 | R98C | 2475 | PTPN11 | Q06124 | D286V |
| 1013 | GLRA3 | O75311 | R98S | 2476 | PTPN11 | Q06124 | P33S |
| 1014 | GLRA3 | O75311 | R304Q | 2477 | PTPN11 | Q06124 | P33L |
| 1015 | GLRA3 | O75311 | P263S | 2478 | PTPN11 | Q06124 | L262F |
| 1016 | GLRA3 | O75311 | L265V | 2479 | PTPN11 | Q06124 | S44F |
| 1017 | GLRA3 | O75311 | Y261C | 2480 | PTPN11 | Q06124 | F285L |
| 1018 | GLRA3 | O75311 | L325F | 2481 | PTPN11 | Q06124 | M82T |
| 1019 | GLRA3 | O75311 | V268F | 2482 | PTPN11 | Q06124 | M82V |
| 1020 | GLRA3 | O75311 | V293M | 2483 | PTPN11 | Q06124 | Q271E |
| 1021 | GLRA3 | O75311 | T295M | 2484 | PTPN11 | Q06124 | W248C |
| 1022 | GLRA3 | O75311 | G254R | 2485 | PTPN11 | Q06124 | I54T |
| 1023 | GLRA3 | O75311 | M260K | 2486 | PTPN11 | Q06124 | R220C |
| 1024 | GLRA3 | O75311 | L257P | 2487 | PTPN11 | Q06124 | R220H |
| 1025 | GLRA3 | O75311 | S191R | 2488 | PTPN11 | Q06124 | P38H |
| 1026 | GLRA3 | O75311 | M260I | 2489 | PTPN11 | Q06124 | G115E |
| 1027 | GLRA3 | O75311 | P263L | 2490 | PTPN11 | Q06124 | P284L |
| 1028 | GLRA3 | O75311 | I163V | 2491 | PTPN11 | Q06124 | D40G |
| 1029 | GLRA3 | O75311 | S300N | 2492 | PTPN11 | Q06124 | F285S |
| 1030 | GLRA3 | O75311 | F132L | 2493 | PTPN11 | Q06124 | G268S |
| 1031 | GLRA3 | O75311 | K314N | 2494 | PYGL | P06737 | D340Y |
| 1032 | GLRA3 | O75311 | R152K | 2495 | PYGL | P06737 | R67C |
| 1033 | GLRA3 | O75311 | D317H | 2496 | PYGL | P06737 | P195S |
| 1034 | GLRA3 | O75311 | S311L | 2497 | PYGL | P06737 | T229N |
| 1035 | GLRA3 | O75311 | L265I | 2498 | PYGL | P06737 | N339I |
| 1036 | GLRA3 | O75311 | Q299E | 2499 | PYGL | P06737 | H63R |
| 1037 | GLRA3 | O75311 | R152G | 2500 | PYGL | P06737 | R570S |
| 1038 | GLRA3 | O75311 | T195K | 2501 | PYGL | P06737 | R311H |
| 1039 | GLRA3 | O75311 | D130N | 2502 | PYGL | P06737 | I69F |
| 1040 | GLS | O94925 | A485T | 2503 | PYGL | P06737 | G695R |
| 1041 | GLS | O94925 | I339S | 2504 | PYGL | P06737 | G678V |
| 1042 | GLS | O94925 | I250L | 2505 | PYGL | P06737 | E89D |
| 1043 | GLS | O94925 | R387Q | 2506 | PYGL | P06737 | G135S |
| 1044 | GLS | O94925 | S419C | 2507 | PYGL | P06737 | H58Y |
| 1045 | GLS | O94925 | K481N | 2508 | PYGL | P06737 | I309V |
| 1046 | GLS | O94925 | R317K | 2509 | PYGL | P06737 | R243C |
| 1047 | GLS | O94925 | G509C | 2510 | PYGL | P06737 | R243H |
| 1048 | GLS | O94925 | L323F | 2511 | PYGL | P06737 | H615Y |
| 1049 | GNPDA1 | P46926 | R158C | 2512 | PYGL | P06737 | Y821F |
| 1050 | GNPDA1 | P46926 | T187M | 2513 | PYGL | P06737 | F286L |
| 1051 | GNPDA1 | P46926 | G136A | 2514 | PYGL | P06737 | H378R |
| 1052 | GNPDA1 | P46926 | A145V | 2515 | PYGL | P06737 | F197L |
| 1053 | GNPDA1 | P46926 | L39H | 2516 | PYGL | P06737 | G187E |
| 1054 | GNPDA1 | P46926 | G150V | 2517 | PYGL | P06737 | G613R |
| 1055 | GNPDA1 | P46926 | A185V | 2518 | PYGL | P06737 | F54C |
| 1056 | GNPDA1 | P46926 | T161P | 2519 | PYGL | P06737 | A611T |
| 1057 | GNPDA1 | P46926 | C118Y | 2520 | PYGL | P06737 | E383K |
| 1058 | GNPDA1 | P46926 | F229L | 2521 | PYGL | P06737 | S668F |
| 1059 | GNPDA1 | P46926 | M184T | 2522 | PYGL | P06737 | I308T |
| 1060 | GNPDA1 | P46926 | G139C | 2523 | PYGL | P06737 | H74Q |
| 1061 | GRIA2 | P42262 | S727Y | 2524 | PYGL | P06737 | N679D |
| 1062 | GRIA2 | P42262 | T728M | 2525 | PYGL | P06737 | R194L |
| 1063 | GRIA2 | P42262 | D672Y | 2526 | PYGL | P06737 | F287S |
| 1064 | GRIA2 | P42262 | D672G | 2527 | PYGL | P06737 | D228Y |
| 1065 | GRIA2 | P42262 | D749N | 2528 | PYGM | P11217 | R570Q |
| 1066 | GRIA2 | P42262 | G752C | 2529 | PYGM | P11217 | R570W |
| 1067 | GRIA2 | P42262 | K677E | 2530 | PYGM | P11217 | R194W |
| 1068 | GRIA2 | P42262 | M729R | 2531 | PYGM | P11217 | D694V |
| 1069 | GRIA2 | P42262 | M729T | 2532 | PYGM | P11217 | R320S |
| 1070 | GRIA2 | P42262 | E678K | 2533 | PYGM | P11217 | R320H |
| 1071 | GRIA2 | P42262 | E678V | 2534 | PYGM | P11217 | T376A |
| 1072 | GRIA2 | P42262 | A498V | 2535 | PYGM | P11217 | Y614H |
| 1073 | GRIA2 | P42262 | A498P | 2536 | PYGM | P11217 | G135R |
| 1074 | GRIA2 | P42262 | S673F | 2537 | PYGM | P11217 | K483N |
| 1075 | GRIA2 | P42262 | T707A | 2538 | PYGM | P11217 | A610S |
| 1076 | GRIA2 | P42262 | M484I | 2539 | PYGM | P11217 | A610P |
| 1077 | GRIA2 | P42262 | T501N | 2540 | PYGM | P11217 | A611T |
| 1078 | GRIA2 | P42262 | A770T | 2541 | PYGM | P11217 | S668F |
| 1079 | GRIA2 | P42262 | Y426C | 2542 | PYGM | P11217 | E573K |
| 1080 | GRIA2 | P42262 | G754D | 2543 | PYGM | P11217 | R243H |
| 1081 | GRIA2 | P42262 | G520W | 2544 | PYGM | P11217 | G676V |
| 1082 | GRIA2 | P42262 | G520R | 2545 | PYGM | P11217 | G676C |
| 1083 | GRIA2 | P42262 | D749H | 2546 | PYGM | P11217 | R771Q |
| 1084 | GRIA2 | P42262 | F512L | 2547 | PYGM | P11217 | D307G |
| 1085 | GRIA2 | P42262 | L725F | 2548 | PYGM | P11217 | E288K |
| 1086 | GRIA2 | P42262 | S522C | 2549 | PYGM | P11217 | G486S |
| 1087 | GRIA2 | P42262 | S750F | 2550 | PYGM | P11217 | A617T |
| 1088 | GRIA2 | P42262 | S513T | 2551 | PYGM | P11217 | T379M |
| 1089 | GRIA2 | P42262 | P499Q | 2552 | PYGM | P11217 | R82K |
| 1090 | GRIA2 | P42262 | Y445H | 2553 | PYGM | P11217 | T484I |
| 1091 | GRIA2 | P42262 | A473D | 2554 | PYGM | P11217 | Y821H |
| 1092 | GRIA2 | P42262 | A473S | 2555 | PYGM | P11217 | R310H |
| 1093 | GRIA2 | P42262 | F679C | 2556 | RNMT | O43148 | W178C |
| 1094 | GRIA2 | P42262 | I502V | 2557 | RNMT | O43148 | D350Y |
| 1095 | GRIA2 | P42262 | F679I | 2558 | RNMT | O43148 | N424S |
| 1096 | GRIA2 | P42262 | F679L | 2559 | RNMT | O43148 | T259S |
| 1097 | GRIN1 | Q05586 | M785V | 2560 | RNMT | O43148 | P376L |
| 1098 | GRIN2A | Q12879 | I514T | 2561 | RNMT | O43148 | H288Y |
| 1099 | GRIN2A | Q12879 | K484M | 2562 | RNMT | O43148 | P369S |
| 1100 | GRIN2A | Q12879 | T758N | 2563 | RNMT | O43148 | F285C |
| 1101 | GRIN2A | Q12879 | R692T | 2564 | RNMT | O43148 | V366M |
| 1102 | GRIN2A | Q12879 | L735M | 2565 | RNMT | O43148 | C356Y |
| 1103 | GRIN2A | Q12879 | G532E | 2566 | RNMT | O43148 | P452L |
| 1104 | GRIN2A | Q12879 | A778T | 2567 | RNMT | O43148 | E380K |
| 1105 | GRIN2A | Q12879 | L411P | 2568 | RNMT | O43148 | S232F |
| 1106 | GRIN2A | Q12879 | G510D | 2569 | RNMT | O43148 | M300R |
| 1107 | GRIN2A | Q12879 | S511L | 2570 | RNMT | O43148 | V368A |
| 1108 | GRIN2A | Q12879 | P686L | 2571 | RNMT | O43148 | E265K |
| 1109 | GRIN2A | Q12879 | A733S | 2572 | RNMT | O43148 | L377S |
| 1110 | GRIN2A | Q12879 | T531M | 2573 | RNMT | O43148 | C206Y |
| 1111 | GRIN2A | Q12879 | V529L | 2574 | RNMT | O43148 | L468F |
| 1112 | GRIN2A | Q12879 | G760S | 2575 | RNMT | O43148 | R450G |
| 1113 | GRIN2A | Q12879 | A733T | 2576 | RNMT | O43148 | R450W |
| 1114 | GRIN2A | Q12879 | E789K | 2577 | RNMT | O43148 | D298Y |
| 1115 | GRIN2A | Q12879 | D731N | 2578 | RNMT | O43148 | R327L |
| 1116 | GRIN2A | Q12879 | T513N | 2579 | RNMT | O43148 | R327C |
| 1117 | GRIN2A | Q12879 | V713I | 2580 | RNMT | O43148 | R327H |
| 1118 | GRIN2A | Q12879 | R692K | 2581 | RNMT | O43148 | S465N |
| 1119 | GRIN2A | Q12879 | G762E | 2582 | RNMT | O43148 | V368F |
| 1120 | GRIN2A | Q12879 | G762R | 2583 | RNMT | O43148 | E397Q |
| 1121 | GRIN2A | Q12879 | F782I | 2584 | RNMT | O43148 | M381R |
| 1122 | GRIN2A | Q12879 | F524V | 2585 | RNMT | O43148 | M444T |
| 1123 | GRIN2A | Q12879 | F524L | 2586 | RORC | P51449 | T375M |
| 1124 | GRIN2A | Q12879 | S519C | 2587 | RORC | P51449 | F486V |
| 1125 | GRIN2A | Q12879 | L777Q | 2588 | RORC | P51449 | I350S |
| 1126 | GRIN2A | Q12879 | A732V | 2589 | RORC | P51449 | A327D |
| 1127 | GRIN2A | Q12879 | A732T | 2590 | RORC | P51449 | A327S |
| 1128 | GRIN2A | Q12879 | A757V | 2591 | RORC | P51449 | A497T |
| 1129 | GRIN2A | Q12879 | E691D | 2592 | RORC | P51449 | Q487K |
| 1130 | GRIN2A | Q12879 | F459S | 2593 | RORC | P51449 | Q286H |
| 1131 | GRIN2A | Q12879 | L512F | 2594 | RORC | P51449 | C320R |
| 1132 | GRIN2A | Q12879 | E691K | 2595 | RORC | P51449 | C393Y |
| 1133 | GRIN2A | Q12879 | I497F | 2596 | RORC | P51449 | M385I |
| 1134 | GRIN2A | Q12879 | I497S | 2597 | RORC | P51449 | F388L |
| 1135 | GRIN2A | Q12879 | D776Y | 2598 | RORC | P51449 | Y502N |
| 1136 | GRIN2A | Q12879 | V783A | 2599 | RORC | P51449 | S404F |
| 1137 | GRIN2A | Q12879 | G688E | 2600 | RORC | P51449 | Y369C |
| 1138 | GRIN2A | Q12879 | T684K | 2601 | RORC | P51449 | F506L |
| 1139 | GRIN2A | Q12879 | T690M | 2602 | RORC | P51449 | V331M |
| 1140 | GRIN2A | Q12879 | I533N | 2603 | RORC | P51449 | P499S |
| 1141 | GRIN2A | Q12879 | I775T | 2604 | RORC | P51449 | M365V |
| 1142 | GRIN2A | Q12879 | L411M | 2605 | RORC | P51449 | G383S |
| 1143 | GRIN2A | Q12879 | V685M | 2606 | RORC | P51449 | G383D |
| 1144 | GRIN2A | Q12879 | V685L | 2607 | RORC | P51449 | F377Y |
| 1145 | GRIN2A | Q12879 | S525F | 2608 | RORC | P51449 | F401L |
| 1146 | GRIN2A | Q12879 | M788I | 2609 | RRM1 | P23921 | L205F |
| 1147 | GRIN2A | Q12879 | G510A | 2610 | RRM1 | P23921 | D287N |
| 1148 | GRIN2A | Q12879 | T759N | 2611 | RRM1 | P23921 | D7E |
| 1149 | GRIN2A | Q12879 | D776N | 2612 | RRM1 | P23921 | I18F |
| 1150 | GRIN2A | Q12879 | D776E | 2613 | RRM1 | P23921 | R284Q |
| 1151 | GRIN2A | Q12879 | R518C | 2614 | RRM1 | P23921 | G264W |
| 1152 | GRIN2A | Q12879 | R518H | 2615 | RRM1 | P23921 | G247R |
| 1153 | GRIN2A | Q12879 | R518L | 2616 | RRM1 | P23921 | S84C |
| 1154 | GRM5 | P41594 | K396T | 2617 | RRM1 | P23921 | A283T |
| 1155 | GRM5 | P41594 | M281K | 2618 | RRM1 | P23921 | Y285C |
| 1156 | GRM5 | P41594 | P193T | 2619 | RRM1 | P23921 | V43L |
| 1157 | GRM5 | P41594 | P193S | 2620 | RRM1 | P23921 | G259D |
| 1158 | GRM5 | P41594 | P193L | 2621 | RRM1 | P23921 | R256Q |
| 1159 | GRM5 | P41594 | R190S | 2622 | RRM1 | P23921 | D16H |
| 1160 | GRM5 | P41594 | R61S | 2623 | RRM1 | P23921 | T58A |
| 1161 | GRM5 | P41594 | G624S | 2624 | RRM1 | P23921 | E431D |
| 1162 | GRM5 | P41594 | L808F | 2625 | RRM1 | P23921 | I44F |
| 1163 | GRM5 | P41594 | L808I | 2626 | RRM1 | P23921 | G259S |
| 1164 | GRM5 | P41594 | L808R | 2627 | RRM1 | P23921 | A296T |
| 1165 | GRM5 | P41594 | A155V | 2628 | RRM1 | P23921 | V3L |
| 1166 | GRM5 | P41594 | L179Q | 2629 | RRM1 | P23921 | V3M |
| 1167 | GRM5 | P41594 | S805L | 2630 | RRM1 | P23921 | L47F |
| 1168 | GRM5 | P41594 | V192L | 2631 | RRM1 | P23921 | P294L |
| 1169 | GRM5 | P41594 | T801I | 2632 | RRM1 | P23921 | I22F |
| 1170 | GRM5 | P41594 | F804L | 2633 | RRM1 | P23921 | R212C |
| 1171 | GRM5 | P41594 | I784V | 2634 | RRM1 | P23921 | C254F |
| 1172 | GRM5 | P41594 | L627Q | 2635 | RRM1 | P23921 | R293C |
| 1173 | GRM5 | P41594 | T777R | 2636 | RRM1 | P23921 | R293H |
| 1174 | GRM5 | P41594 | W307C | 2637 | RRM1 | P23921 | N85S |
| 1175 | GRM5 | P41594 | S304R | 2638 | RRM1 | P23921 | N85D |
| 1176 | GRM5 | P41594 | G628C | 2639 | RRM1 | P23921 | N354H |
| 1177 | GRM5 | P41594 | F818L | 2640 | RXRA | P19793 | T278I |
| 1178 | GRM5 | P41594 | A623D | 2641 | RXRA | P19793 | W305C |
| 1179 | GRM5 | P41594 | L738I | 2642 | RXRA | P19793 | R316H |
| 1180 | GRM5 | P41594 | L744V | 2643 | RXRA | P19793 | R316L |
| 1181 | GRM5 | P41594 | F277V | 2644 | RXRA | P19793 | A311T |
| 1182 | GRM5 | P41594 | G739I | 2645 | RXRA | P19793 | L308V |
| 1183 | GRM5 | P41594 | G739E | 2646 | RXRA | P19793 | G343C |
| 1184 | GRM5 | P41594 | L622I | 2647 | RXRA | P19793 | A272T |
| 1185 | GRM5 | P41594 | I621T | 2648 | RXRA | P19793 | A344T |
| 1186 | GRM5 | P41594 | G650D | 2649 | RXRA | P19793 | V342M |
| 1187 | GRM5 | P41594 | V313A | 2650 | RXRA | P19793 | S312A |
| 1188 | GRM5 | P41594 | D394N | 2651 | RXRA | P19793 | G429W |
| 1189 | GRM5 | P41594 | R68I | 2652 | RXRA | P19793 | G329R |
| 1190 | GRM5 | P41594 | G652C | 2653 | RXRA | P19793 | S317C |
| 1191 | GRM5 | P41594 | G745R | 2654 | RXRA | P19793 | S317F |
| 1192 | GRM5 | P41594 | D195N | 2655 | RXRA | P19793 | V265I |
| 1193 | GRM5 | P41594 | L653F | 2656 | RXRA | P19793 | D273G |
| 1194 | GRM5 | P41594 | L653V | 2657 | RXRA | P19793 | D273N |
| 1195 | GRM5 | P41594 | W785R | 2658 | RXRA | P19793 | E434K |
| 1196 | GRM5 | P41594 | S153Y | 2659 | RXRA | P19793 | H435Y |
| 1197 | GRM5 | P41594 | A623S | 2660 | RXRA | P19793 | G429V |
| 1198 | GRM5 | P41594 | S176R | 2661 | RXRA | P19793 | G429R |
| 1199 | GRM5 | P41594 | S176I | 2662 | RXRA | P19793 | R316C |
| 1200 | GRM5 | P41594 | P193H | 2663 | RXRA | P19793 | I345M |
| 1201 | GRM5 | P41594 | R61H | 2664 | SAMHD1 | Q9Y3Z3 | G324E |
| 1202 | GRM5 | P41594 | R61C | 2665 | SAMHD1 | Q9Y3Z3 | R164Q |
| 1203 | GRM5 | P41594 | A174T | 2666 | SAMHD1 | Q9Y3Z3 | G153V |
| 1204 | GRM5 | P41594 | Y172C | 2667 | SAMHD1 | Q9Y3Z3 | D353H |
| 1205 | GRM5 | P41594 | Y172H | 2668 | SAMHD1 | Q9Y3Z3 | K523E |
| 1206 | GRM5 | P41594 | Q647L | 2669 | SAMHD1 | Q9Y3Z3 | K312T |
| 1207 | GRM5 | P41594 | W100L | 2670 | SAMHD1 | Q9Y3Z3 | C522Y |
| 1208 | GRM5 | P41594 | S151F | 2671 | SAMHD1 | Q9Y3Z3 | D207Y |
| 1209 | GRM5 | P41594 | S151P | 2672 | SAMHD1 | Q9Y3Z3 | R366H |
| 1210 | GRM5 | P41594 | S151Y | 2673 | SAMHD1 | Q9Y3Z3 | R366C |
| 1211 | HBA1 | P69905 | A131T | 2674 | SAMHD1 | Q9Y3Z3 | R333H |
| 1212 | HBA1 | P69905 | T138I | 2675 | SAMHD1 | Q9Y3Z3 | D120N |
| 1213 | HBA1 | P69905 | D127Y | 2676 | SAMHD1 | Q9Y3Z3 | G151V |
| 1214 | HBA1 | P69905 | F99L | 2677 | SAMHD1 | Q9Y3Z3 | G124D |
| 1215 | HBA1 | P69905 | K140N | 2678 | SAMHD1 | Q9Y3Z3 | R371C |
| 1216 | HBB | P68871 | E102D | 2679 | SAMHD1 | Q9Y3Z3 | R371H |
| 1217 | HBB | P68871 | V35L | 2680 | SAMHD1 | Q9Y3Z3 | D501Y |
| 1218 | HBB | P68871 | V35F | 2681 | SAMHD1 | Q9Y3Z3 | R220Q |
| 1219 | HBB | P68871 | Q132L | 2682 | SAMHD1 | Q9Y3Z3 | R220L |
| 1220 | HBB | P68871 | Q128H | 2683 | SAMHD1 | Q9Y3Z3 | R451C |
| 1221 | HBB | P68871 | P37A | 2684 | SAMHD1 | Q9Y3Z3 | S302C |
| 1222 | HBB | P68871 | Q132H | 2685 | SAMHD1 | Q9Y3Z3 | R451P |
| 1223 | HBB | P68871 | R105K | 2686 | SAMHD1 | Q9Y3Z3 | R318S |
| 1224 | HGF | P14210 | L634I | 2687 | SAMHD1 | Q9Y3Z3 | Q326L |
| 1225 | HGF | P14210 | H554Q | 2688 | SAMHD1 | Q9Y3Z3 | S302Y |
| 1226 | HGF | P14210 | R708L | 2689 | SAMHD1 | Q9Y3Z3 | D218H |
| 1227 | HGF | P14210 | F706V | 2690 | SAMHD1 | Q9Y3Z3 | K116E |
| 1228 | HGF | P14210 | D552N | 2691 | SAMHD1 | Q9Y3Z3 | R333C |
| 1229 | HGF | P14210 | R685I | 2692 | SAMHD1 | Q9Y3Z3 | R333S |
| 1230 | HGF | P14210 | Y625C | 2693 | SAMHD1 | Q9Y3Z3 | M362L |
| 1231 | HGF | P14210 | R533L | 2694 | SAMHD1 | Q9Y3Z3 | K377N |
| 1232 | HGF | P14210 | E656Q | 2695 | SAMHD1 | Q9Y3Z3 | H162Q |
| 1233 | HGF | P14210 | D578N | 2696 | SAMHD1 | Q9Y3Z3 | S214P |
| 1234 | HGF | P14210 | D578E | 2697 | SERPINC1 | P01008 | K43N |
| 1235 | HGF | P14210 | D578Y | 2698 | SERPINC1 | P01008 | M347V |
| 1236 | HGF | P14210 | I505K | 2699 | SERPINC1 | P01008 | R161Q |
| 1237 | HGF | P14210 | E670K | 2700 | SERPINC1 | P01008 | T433S |
| 1238 | HGF | P14210 | G675S | 2701 | SERPINC1 | P01008 | S426L |
| 1239 | HGF | P14210 | P537N | 2702 | SERPINC1 | P01008 | V349M |
| 1240 | HGF | P14210 | P537T | 2703 | SERPINC1 | P01008 | G424D |
| 1241 | HGF | P14210 | E656K | 2704 | SERPINC1 | P01008 | E264K |
| 1242 | HGF | P14210 | S577T | 2705 | SERPINC1 | P01008 | T418I |
| 1243 | HGF | P14210 | G616V | 2706 | SERPINC1 | P01008 | E145K |
| 1244 | HGF | P14210 | G696V | 2707 | SERPINC1 | P01008 | E145D |
| 1245 | HGF | P14210 | D540E | 2708 | SERPINC1 | P01008 | N77S |
| 1246 | HGF | P14210 | G576R | 2709 | SERPINC1 | P01008 | R79C |
| 1247 | HGF | P14210 | G576E | 2710 | SERPINC1 | P01008 | R79L |
| 1248 | HGF | P14210 | R539Q | 2711 | SERPINC1 | P01008 | R79H |
| 1249 | HGF | P14210 | R514K | 2712 | SERPINC1 | P01008 | N428K |
| 1250 | HGF | P14210 | I664N | 2713 | SERPINC1 | P01008 | S144F |
| 1251 | HGF | P14210 | G671V | 2714 | SERPINC1 | P01008 | P429L |
| 1252 | HGF | P14210 | G671R | 2715 | SERPINC1 | P01008 | R45W |
| 1253 | HGF | P14210 | R695H | 2716 | SERPINC1 | P01008 | C40Y |
| 1254 | HGF | P14210 | R695C | 2717 | SERPINC1 | P01008 | F153L |
| 1255 | HGF | P14210 | P668S | 2718 | SERPINC1 | P01008 | V432A |
| 1256 | HGF | P14210 | P668L | 2719 | SERPINC1 | P01008 | R56C |
| 1257 | HGF | P14210 | A661D | 2720 | SERPINC1 | P01008 | E446K |
| 1258 | HGF | P14210 | R708Q | 2721 | SERPINC1 | P01008 | P73S |
| 1259 | HGF | P14210 | H551Y | 2722 | SERPINC1 | P01008 | A42T |
| 1260 | HGF | P14210 | S655C | 2723 | SERPINC1 | P01008 | R56H |
| 1261 | HGF | P14210 | H645N | 2724 | SERPINC1 | P01008 | M346V |
| 1262 | HGF | P14210 | G667E | 2725 | SERPINC1 | P01008 | F434L |
| 1263 | HGF | P14210 | G667A | 2726 | SERPINC1 | P01008 | R78W |
| 1264 | HGF | P14210 | R647P | 2727 | SERPINC1 | P01008 | Q286K |
| 1265 | HGF | P14210 | Y572C | 2728 | SERPINC1 | P01008 | G288S |
| 1266 | HGF | P14210 | G665R | 2729 | SERPINC1 | P01008 | E145Q |
| 1267 | HGF | P14210 | V650E | 2730 | SERPINC1 | P01008 | E287K |
| 1268 | HGF | P14210 | V650M | 2731 | SERPINE1 | P05121 | K230T |
| 1269 | HGF | P14210 | G621R | 2732 | SERPINE1 | P05121 | D254N |
| 1270 | HGF | P14210 | Y712S | 2733 | SERPINE1 | P05121 | R386W |
| 1271 | HGF | P14210 | D672N | 2734 | SERPINE1 | P05121 | N232S |
| 1272 | HGF | P14210 | V692L | 2735 | SERPINE1 | P05121 | T228N |
| 1273 | HGF | P14210 | V692F | 2736 | SERPINE1 | P05121 | F132Y |
| 1274 | HGF | P14210 | C535W | 2737 | SERPINE1 | P05121 | R154K |
| 1275 | HGF | P14210 | K649M | 2738 | SERPINE1 | P05121 | R141W |
| 1276 | HGF | P14210 | D626Y | 2739 | SERPINE1 | P05121 | R124Q |
| 1277 | HK1 | P19367 | T153M | 2740 | SERPINE1 | P05121 | D125H |
| 1278 | HK1 | P19367 | G87E | 2741 | SERPINE1 | P05121 | F136S |
| 1279 | HK1 | P19367 | G862R | 2742 | SERPINE1 | P05121 | F136C |
| 1280 | HK1 | P19367 | M300I | 2743 | SERPINE1 | P05121 | F259L |
| 1281 | HK1 | P19367 | G231S | 2744 | SERPINE1 | P05121 | V122I |
| 1282 | HK1 | P19367 | E708K | 2745 | SERPINE1 | P05121 | M289I |
| 1283 | HK1 | P19367 | G414R | 2746 | SERPINE1 | P05121 | P250A |
| 1284 | HK1 | P19367 | R91Q | 2747 | SERPINE1 | P05121 | D161E |
| 1285 | HK1 | P19367 | G299C | 2748 | SERPINE1 | P05121 | E353D |
| 1286 | HK1 | P19367 | G450S | 2749 | SERPINE1 | P05121 | E248K |
| 1287 | HK1 | P19367 | L83V | 2750 | SERPINE1 | P05121 | P201L |
| 1288 | HK1 | P19367 | L228M | 2751 | SERPINE1 | P05121 | T143K |
| 1289 | HK1 | P19367 | G679V | 2752 | SERPINE1 | P05121 | V147L |
| 1290 | HRAS | P01112 | D108Y | 2753 | SERPINE1 | P05121 | R154I |
| 1291 | HRAS | P01112 | A134T | 2754 | SERPINE1 | P05121 | D148N |
| 1292 | HRAS | P01112 | A134S | 2755 | SERPINE1 | P05121 | I158T |
| 1293 | HRAS | P01112 | T20I | 2756 | SERPINE1 | P05121 | A119T |
| 1294 | HRAS | P01112 | K147N | 2757 | SERPINE1 | P05121 | A119V |
| 1295 | HRAS | P01112 | V9M | 2758 | SERPINE1 | P05121 | E353K |
| 1296 | HRAS | P01112 | A83D | 2759 | SERPINE1 | P05121 | W162C |
| 1297 | HRAS | P01112 | G15D | 2760 | SIRT1 | Q96EB6 | E208K |
| 1298 | HRAS | P01112 | G15S | 2761 | SIRT1 | Q96EB6 | F273S |
| 1299 | HRAS | P01112 | D57N | 2762 | SIRT1 | Q96EB6 | D292E |
| 1300 | HRAS | P01112 | M72I | 2763 | SIRT1 | Q96EB6 | D292Y |
| 1301 | HRAS | P01112 | A18V | 2764 | SIRT1 | Q96EB6 | E230K |
| 1302 | HRAS | P01112 | A18T | 2765 | SIRT1 | Q96EB6 | N417S |
| 1303 | HRAS | P01112 | T148A | 2766 | SIRT1 | Q96EB6 | P293S |
| 1304 | HRAS | P01112 | V14G | 2767 | SIRT1 | Q96EB6 | D298H |
| 1305 | HRAS | P01112 | Y32C | 2768 | SIRT1 | Q96EB6 | A295S |
| 1306 | HRAS | P01112 | D92N | 2769 | SIRT1 | Q96EB6 | P207L |
| 1307 | HRAS | P01112 | H94Y | 2770 | SIRT1 | Q96EB6 | S229L |
| 1308 | HRAS | P01112 | S145L | 2771 | SIRT1 | Q96EB6 | R199Q |
| 1309 | HRAS | P01112 | E62G | 2772 | SIRT1 | Q96EB6 | P447S |
| 1310 | HRAS | P01112 | G10A | 2773 | SIRT1 | Q96EB6 | I347K |
| 1311 | HRAS | P01112 | Y71H | 2774 | SIRT1 | Q96EB6 | L228I |
| 1312 | HRAS | P01112 | G60S | 2775 | SIRT3 | Q9NTG7 | D172E |
| 1313 | HRAS | P01112 | G60V | 2776 | SIRT3 | Q9NTG7 | V292L |
| 1314 | HRAS | P01112 | A11S | 2777 | SIRT3 | Q9NTG7 | G232V |
| 1315 | HRAS | P01112 | G60D | 2778 | SIRT3 | Q9NTG7 | P297S |
| 1316 | HRAS | P01112 | L120V | 2779 | SIRT3 | Q9NTG7 | S149N |
| 1317 | HRAS | P01112 | L120P | 2780 | SIRT3 | Q9NTG7 | H248N |
| 1318 | HRAS | P01112 | T144I | 2781 | SIRT3 | Q9NTG7 | G319V |
| 1319 | HRAS | P01112 | V81M | 2782 | SIRT3 | Q9NTG7 | Q170H |
| 1320 | HRAS | P01112 | D69N | 2783 | SIRT3 | Q9NTG7 | S329G |
| 1321 | HRAS | P01112 | D69H | 2784 | SIRT3 | Q9NTG7 | N229T |
| 1322 | HRAS | P01112 | S106P | 2785 | SIRT3 | Q9NTG7 | I291S |
| 1323 | HRAS | P01112 | E31K | 2786 | SIRT3 | Q9NTG7 | S329N |
| 1324 | HRAS | P01112 | Y137D | 2787 | SIRT3 | Q9NTG7 | V306M |
| 1325 | HRAS | P01112 | E91Q | 2788 | SIRT3 | Q9NTG7 | F192L |
| 1326 | HRAS | P01112 | A83V | 2789 | SIRT3 | Q9NTG7 | F293L |
| 1327 | HRAS | P01112 | E91K | 2790 | SIRT3 | Q9NTG7 | E177K |
| 1328 | HRAS | P01112 | C118Y | 2791 | SLC1A3 | P43003 | S365I |
| 1329 | HRAS | P01112 | R135Q | 2792 | SLC1A3 | P43003 | A446V |
| 1330 | HRAS | P01112 | S89T | 2793 | SLC1A3 | P43003 | R385H |
| 1331 | HRAS | P01112 | R68Q | 2794 | SLC1A3 | P43003 | N398K |
| 1332 | HRAS | P01112 | R68W | 2795 | SLC1A3 | P43003 | A446E |
| 1333 | HRAS | P01112 | S17G | 2796 | SLC1A3 | P43003 | I397V |
| 1334 | HRAS | P01112 | I36V | 2797 | SLC1A3 | P43003 | L448Q |
| 1335 | HRAS | P01112 | K88E | 2798 | SLC1A3 | P43003 | L448M |
| 1336 | HRAS | P01112 | S106L | 2799 | SLC1A3 | P43003 | A395T |
| 1337 | HRAS | P01112 | D107G | 2800 | SLC1A3 | P43003 | R479Q |
| 1338 | HRAS | P01112 | K16T | 2801 | SLC1A3 | P43003 | R479W |
| 1339 | HRAS | P01112 | K16R | 2802 | SLC1A3 | P43003 | D472E |
| 1340 | HRAS | P01112 | R102L | 2803 | SLC1A3 | P43003 | V484I |
| 1341 | HRAS | P01112 | D119H | 2804 | SLC1A3 | P43003 | W473C |
| 1342 | HRAS | P01112 | D119N | 2805 | SLC1A3 | P43003 | A359V |
| 1343 | HSPA5 | P11021 | R97W | 2806 | SLC1A3 | P43003 | M399K |
| 1344 | HSPA5 | P11021 | L35M | 2807 | SLC1A3 | P43003 | V393I |
| 1345 | HSPA5 | P11021 | D560H | 2808 | SLC1A3 | P43003 | V393G |
| 1346 | HSPA5 | P11021 | S571Y | 2809 | SLC1A3 | P43003 | Y127C |
| 1347 | HSPA5 | P11021 | K296T | 2810 | SLC1A3 | P43003 | G394E |
| 1348 | HSPA5 | P11021 | P63Q | 2811 | SLC1A3 | P43003 | P392L |
| 1349 | HSPA5 | P11021 | V461A | 2812 | SLC1A3 | P43003 | R388K |
| 1350 | HSPA5 | P11021 | P369L | 2813 | SLC1A3 | P43003 | V494A |
| 1351 | HSPA5 | P11021 | I426F | 2814 | SLC1A3 | P43003 | T402A |
| 1352 | HSPA5 | P11021 | D231N | 2815 | SLC1A3 | P43003 | V390M |
| 1353 | HSPA5 | P11021 | L436P | 2816 | SLC1A3 | P43003 | G107R |
| 1354 | HSPA5 | P11021 | I463V | 2817 | SLC1A3 | P43003 | I438M |
| 1355 | HSPA5 | P11021 | Y568C | 2818 | SLC1A3 | P43003 | F389L |
| 1356 | HSPA5 | P11021 | G36R | 2819 | SLC1A3 | P43003 | Q445E |
| 1357 | HSPA5 | P11021 | N389K | 2820 | SLC1A3 | P43003 | R477H |
| 1358 | HSPA5 | P11021 | S452P | 2821 | SLC1A3 | P43003 | R477C |
| 1359 | HSPA5 | P11021 | P173L | 2822 | SLC1A3 | P43003 | A115V |
| 1360 | HSPA5 | P11021 | T171S | 2823 | SLC1A3 | P43003 | T450A |
| 1361 | HSPA5 | P11021 | T434A | 2824 | SNRNP200 | O75643 | R558H |
| 1362 | HSPA5 | P11021 | Q496H | 2825 | SNRNP200 | O75643 | P543H |
| 1363 | IDH1 | O75874 | R119Q | 2826 | SNRNP200 | O75643 | F1259C |
| 1364 | IDH1 | O75874 | I112T | 2827 | SNRNP200 | O75643 | E1237K |
| 1365 | IDH1 | O75874 | G286S | 2828 | SNRNP200 | O75643 | V512M |
| 1366 | IDH1 | O75874 | I215F | 2829 | SNRNP200 | O75643 | H618N |
| 1367 | IDH1 | O75874 | G264D | 2830 | SNRNP200 | O75643 | G1708V |
| 1368 | IDH1 | O75874 | G131D | 2831 | SNRNP200 | O75643 | I1681M |
| 1369 | IDH1 | O75874 | A282V | 2832 | SNRNP200 | O75643 | H1236N |
| 1370 | IDH1 | O75874 | S278L | 2833 | SNRNP200 | O75643 | H1457Y |
| 1371 | IDH1 | O75874 | M254I | 2834 | SNRNP200 | O75643 | T1197P |
| 1372 | IDH1 | O75874 | W205L | 2835 | SNRNP200 | O75643 | K1710R |
| 1373 | IDH1 | O75874 | Y285H | 2836 | SNRNP200 | O75643 | V464M |
| 1374 | IDH1 | O75874 | P118L | 2837 | SNRNP200 | O75643 | V1256M |
| 1375 | IDH1 | O75874 | A258T | 2838 | SNRNP200 | O75643 | R483W |
| 1376 | IDH1 | O75874 | K126R | 2839 | THRA | P10827 | G291D |
| 1377 | IDH1 | O75874 | A111D | 2840 | THRA | P10827 | G291S |
| 1378 | IDH1 | O75874 | I128V | 2841 | THRA | P10827 | C255Y |
| 1379 | IDH1 | O75874 | E262Q | 2842 | THRA | P10827 | K220N |
| 1380 | IDH1 | O75874 | Q283H | 2843 | THRA | P10827 | A264T |
| 1381 | IDH1 | O75874 | I113T | 2844 | THRA | P10827 | G290D |
| 1382 | IDH1 | O75874 | P78H | 2845 | THRA | P10827 | E217K |
| 1383 | IDH1 | O75874 | N213T | 2846 | THRA | P10827 | I222M |
| 1384 | IDH1 | O75874 | G263V | 2847 | THRA | P10827 | A214V |
| 1385 | IDH1 | O75874 | N96I | 2848 | THRA | P10827 | K288E |
| 1386 | IDH1 | O75874 | E306D | 2849 | UBE2I | P63279 | P88S |
| 1387 | IDH1 | O75874 | G310R | 2850 | UBE2I | P63279 | R61W |
| 1388 | IDH1 | O75874 | G310V | 2851 | UBE2I | P63279 | F77I |
| 1389 | IDH1 | O75874 | R119W | 2852 | UBE2I | P63279 | P88H |
| 1390 | IDH1 | O75874 | A111T | 2853 | UBE2I | P63279 | A131S |
| 1391 | IDH1 | O75874 | M290I | 2854 | UBE2I | P63279 | F77C |
| 1392 | IDH1 | O75874 | I113V | 2855 | UBE2I | P63279 | A26T |
| 1393 | IDH1 | O75874 | I128S | 2856 | UBE2T | Q9NPD8 | S101Y |
| 1394 | IDH1 | O75874 | A256S | 2857 | UBE2T | Q9NPD8 | H76Y |
| 1395 | IDH2 | P48735 | S326F | 2858 | UBE2T | Q9NPD8 | Y75S |
| 1396 | IDH2 | P48735 | T169I | 2859 | UBE2T | Q9NPD8 | W98C |
| 1397 | IDH2 | P48735 | V161I | 2860 | UBE2T | Q9NPD8 | F63L |
| 1398 | IDH2 | P48735 | R89H | 2861 | UBE2T | Q9NPD8 | I79F |
| 1399 | IDH2 | P48735 | R89C | 2862 | UBE2T | Q9NPD8 | R69Q |
| 1400 | IDH2 | P48735 | A321V | 2863 | UBE2T | Q9NPD8 | I104T |
| 1401 | IDH2 | P48735 | I62M | 2864 | UGDH | O60701 | Y14C |
| 1402 | IDH2 | P48735 | I62V | 2865 | UGDH | O60701 | R230K |
| 1403 | IDH2 | P48735 | E345K | 2866 | UGDH | O60701 | E54V |
| 1404 | IDH2 | P48735 | V294M | 2867 | UGDH | O60701 | R442W |
| 1405 | IDH2 | P48735 | F324V | 2868 | UGDH | O60701 | R346K |
| 1406 | IDH2 | P48735 | T352P | 2869 | UGDH | O60701 | S348F |
| 1407 | IDH2 | P48735 | N367K | 2870 | UGDH | O60701 | A168T |
| 1408 | IDH2 | P48735 | A370T | 2871 | UGDH | O60701 | F265L |
| 1409 | IDH2 | P48735 | Q296R | 2872 | UGDH | O60701 | L227V |
| 1410 | IDH2 | P48735 | R149W | 2873 | UGDH | O60701 | S275N |
| 1411 | IDH2 | P48735 | N136S | 2874 | USP7 | Q93009 | R408K |
| 1412 | IDH2 | P48735 | T434N | 2875 | USP7 | Q93009 | A221V |
| 1413 | IDH2 | P48735 | S249G | 2876 | USP7 | Q93009 | H464Y |
| 1414 | IDH2 | P48735 | G55S | 2877 | USP7 | Q93009 | I354M |
| 1415 | IDH2 | P48735 | R122C | 2878 | USP7 | Q93009 | V296F |
| 1416 | IDH2 | P48735 | Q296K | 2879 | USP7 | Q93009 | Q219P |
| 1417 | IDH2 | P48735 | R353H | 2880 | USP7 | Q93009 | M407R |
| 1418 | IDH3A | P50213 | T342I | 2881 | USP7 | Q93009 | D349N |
| 1419 | IDH3A | P50213 | E287G | 2882 | USP7 | Q93009 | D295G |
| 1420 | IDH3A | P50213 | T101N | 2883 | USP7 | Q93009 | C223Y |
| 1421 | IDH3A | P50213 | K96T | 2884 | USP7 | Q93009 | D481H |
| 1422 | IDH3A | P50213 | I258F | 2885 | VCP | P55072 | R377H |
| 1423 | IDH3A | P50213 | P304T | 2886 | VCP | P55072 | Y495C |
| 1424 | IDH3G | P51553 | Q259L | 2887 | VCP | P55072 | F503V |
| 1425 | IDH3G | P51553 | D254G | 2888 | VCP | P55072 | I369T |
| 1426 | IGF1R | P08069 | G1085D | 2889 | VCP | P55072 | I369V |
| 1427 | IGF1R | P08069 | D1086N | 2890 | VCP | P55072 | R256Q |
| 1428 | IGF1R | P08069 | L1126V | 2891 | VCP | P55072 | D304N |
| 1429 | IGF1R | P08069 | H1061Y | 2892 | VCP | P55072 | L657I |
| 1430 | IGF1R | P08069 | H1061Q | 2893 | VCP | P55072 | G245R |
| 1431 | IGF1R | P08069 | D1153E | 2894 | VCP | P55072 | P520L |
| 1432 | IGF1R | P08069 | R1134T | 2895 | VCP | P55072 | R359Q |
| 1433 | IGF1R | P08069 | R1084W | 2896 | VCP | P55072 | P646S |
| 1434 | IGF1R | P08069 | F1154L | 2897 | VCP | P55072 | P646Q |
| 1435 | IGF1R | P08069 | T1157M | 2898 | VCP | P55072 | G480R |
| 1436 | IGF1R | P08069 | T1157A | 2899 | VCP | P55072 | F575L |
| 1437 | IGF1R | P08069 | A1051S | 2900 | VCP | P55072 | P571S |
| 1438 | IGF1R | P08069 | L1066Q | 2901 | VCP | P55072 | A255V |
| 1439 | IGF1R | P08069 | M1012I | 2902 | VCP | P55072 | D686G |
| 1440 | IGF1R | P08069 | G1067S | 2903 | VCP | P55072 | G518V |
| 1441 | IGF1R | P08069 | V1053L | 2904 | VCP | P55072 | G370A |
| 1442 | IGF1R | P08069 | I1151V | 2905 | VCP | P55072 | Y517C |
| 1443 | IGF1R | P08069 | M1179V | 2906 | VCP | P55072 | R635T |
| 1444 | IGF1R | P08069 | V1077A | 2907 | WNK1 | Q9H4A3 | H347Y |
| 1445 | IGF1R | P08069 | I1032T | 2908 | WNK1 | Q9H4A3 | H347N |
| 1446 | IGF1R | P08069 | F1047L | 2909 | WNK1 | Q9H4A3 | A372P |
| 1447 | IGF1R | P08069 | S1089R | 2910 | WNK1 | Q9H4A3 | L374Q |
| 1448 | IGF1R | P08069 | E1046K | 2911 | WNK1 | Q9H4A3 | Y236F |
| 1449 | IGF1R | P08069 | V1132I | 2912 | WNK1 | Q9H4A3 | E261D |
| 1450 | IGF1R | P08069 | K1150R | 2913 | WNK1 | Q9H4A3 | K310R |
| 1451 | IGF1R | P08069 | N1049D | 2914 | WNK1 | Q9H4A3 | L299F |
| 1452 | ITGAL | P20701 | M183I | 2915 | WNK1 | Q9H4A3 | L299V |
| 1453 | ITGAL | P20701 | G285R | 2916 | WNK1 | Q9H4A3 | R348H |
| 1454 | ITGAL | P20701 | F159V | 2917 | WNK1 | Q9H4A3 | R348C |
| 1455 | ITGAL | P20701 | E266K | 2918 | WNK1 | Q9H4A3 | L374R |
| 1456 | ITGAL | P20701 | S304T | 2919 | WNK1 | Q9H4A3 | G228D |
| 1457 | ITGAL | P20701 | F178L | 2920 | WNK1 | Q9H4A3 | A248T |
| 1458 | ITGAL | P20701 | D156N | 2921 | WNK1 | Q9H4A3 | W249L |
| 1459 | ITGAL | P20701 | D269N | 2922 | WNK1 | Q9H4A3 | L369F |
| 1460 | ITGAL | P20701 | G163S | 2923 | WNK1 | Q9H4A3 | R341Q |
| 1461 | ITGAL | P20701 | V155L | 2924 | WNK1 | Q9H4A3 | R341G |
| 1462 | ITGAL | P20701 | V155A | 2925 | WNK1 | Q9H4A3 | P344L |
| 1463 | ITGAL | P20701 | L259F |  |  |  |  |

**Table S8.** Summary of all features used in AlloDriver.

| Sequence features (1-8) | |
| --- | --- |
| 1-2: | Shannon entropy and position-specific scoring matrix |
| 3-4: | Blosum62 substitution score and Grantham score |
| 5-8: | Change in volume, delta G, hydropathy and hydrophobicity |
| Structural features (9-24) | |
| 9-10: | Disorder and secondary structure |
| 11-13: | Residue depth, relative accessible surface area and pocket residues |
| 14-23: | Torsion angles, NH-->O_relidx, NH-->O_energy, O-->NH_relidx, O-->NH_energy |
| 24: | Cα B factor |
| Interaction features (25-33) | |
| 25: | Number of degrees |
| 26-27: | The RAPDF and TAP energy |
| 28-33: | Hydrogen bond, ionic interaction, Van der Waals, π-π stacking, π-cation, unspecific interaction |
| Dynamic features (34-41) | |
| 34-35: | Residue fluctuation perturbed by the pocket and global structure from GNMs |
| 36-37: | Residue mechanical stiffness on the pocket and global structure from ANMs |
| 38-41: | Perturbation Response scanning analysis for the pocket and global structure from ANMs |
| Energy feature (42) | |
| 42: | Protein stability change on the point mutation |
